# Nonsymmetrically Substituted 1,1′-Biphenyl-Based Small Molecule Inhibitors of the PD-1/PD-L1 Interaction

**DOI:** 10.1021/acsmedchemlett.4c00042

**Published:** 2024-06-03

**Authors:** Aleksandra Hec-Gałązka, Urszula Tyrcha, Jan Barczyński, Przemyslaw Bielski, Michał Mikitiuk, Ganna P. Gudz, Radosław Kitel, Bogdan Musielak, Jacek Plewka, Tomasz Sitar, Tad A. Holak

**Affiliations:** † Jagiellonian University, Doctoral School of Exact and Natural Sciences, prof. S. Łojasiewicza 11, 30-348 Krakow, Poland; ‡ Department of Organic Chemistry, Faculty of Chemistry, Jagiellonian University, Gronostajowa 2, 30-387 Krakow, Poland; § Recepton Sp. z o.o., ul. Trzy Lipy 3, 80-172 Gdansk, Poland

**Keywords:** PD-1, PD-L1, Small Molecule, Immune Checkpoint Blockade

## Abstract

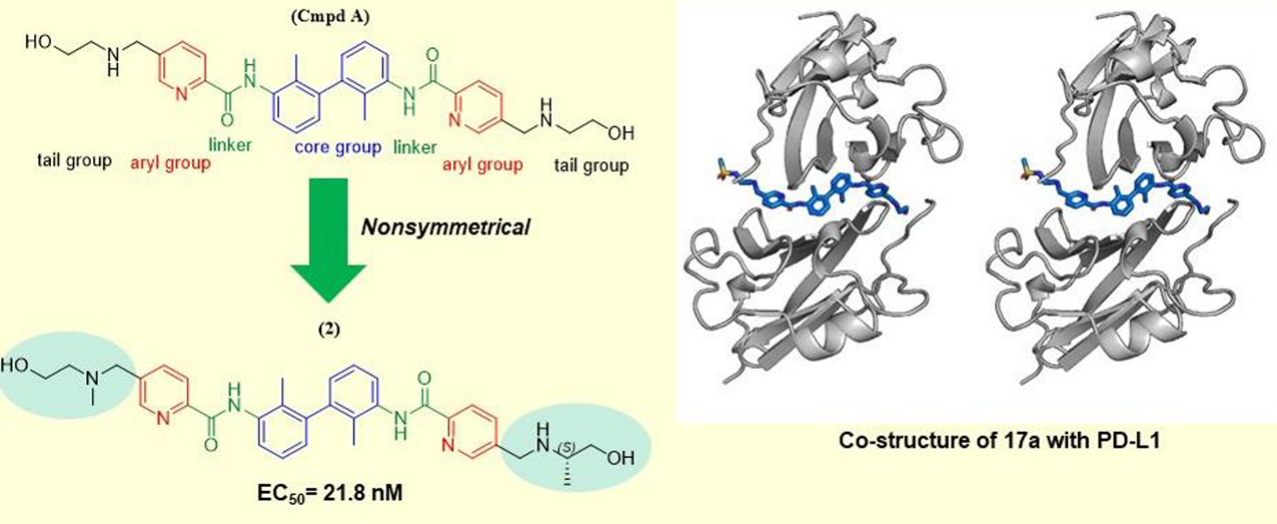

Therapeutic antibodies directed against either programmed cell death-1 protein (PD-1) or its ligand PD-L1 have demonstrated efficacy in the treatment of various cancers. In contrast with antibodies, small molecules have the potential for increased tissue penetration; better pharmacology; and therefore, improved antitumor activity. A series of nonsymmetric C2 inhibitors were synthesized and evaluated for PD-1/PD-L1 interaction inhibition. These compounds induced PD-L1 dimerization and effectively blocked PD-L1/PD-1 interaction in a homogeneous time-resolved fluorescence (HTRF) assay with most inhibitors exhibiting IC_50_ values in the single-digit nM range and below. Their high inhibitory potency was also demonstrated in a cell-based coculture PD-1 signaling assay where **2** exhibited an EC_50_ inhibitory activity of 21.8 nM, which approached that of the PD-L1 antibody durvalumab (EC_50_ = 0.3–1.8 nM). Structural insight into how these inhibitors interact with PD-L1 was gained by using NMR and X-ray cocrystal structure studies. These data support further preclinical evaluation of these compounds as antibody alternatives.

Immune checkpoint inhibitors (ICIs) have fundamentally changed the treatment regimen and prognosis for many cancers^1−3^ by providing long-term clinical responses and even cures in a subset of cancer patients.^4−9^ ICIs based on the antibodies against the PD-1/PD-L1 pathway are currently the cornerstone of this cancer immunotherapy.^1,9^

In spite of their medical and commercial success, immunotherapies based on mAbs have a number of drawbacks,^7,10−12^ including the high production costs, potential immunogenicity, immune-related adverse events (irAEs), and poor solid tumor penetration.^13−15^ In addition, higher rates of toxicity are expected when these drugs are combined with chemotherapy and other immunotherapeutic agents.^2,4^

Small molecule inhibitors are expected to overcome the problems associated with antibody-based therapeutics with advantages such as oral bioavailability, better tumor penetration, longer shelf life, and lower production costs.^16−18^ Despite these potential advantages, the development of small-molecule antagonists has lagged behind mAbs, largely because of the challenge of targeting large, flat interaction surfaces without visible binding pockets, such as those found in PD-1 and PD-L1.^19−23^ Nevertheless, small molecule checkpoint inhibitors have been actively pursued,^15,24−26^ and the ongoing clinical trials of orally bioavailable checkpoint inhibitors are the culmination of these efforts.^15,26,27^

Herein, we show a group of “nonsymmetric” compounds that bind to PD-L1 and are based on an amide linker attached to a biphenyl structure (Table 1). These compounds effectively block the PD-1/PD-L1 interaction in a number of in vitro and ex vivo assays.

**Table 1:**
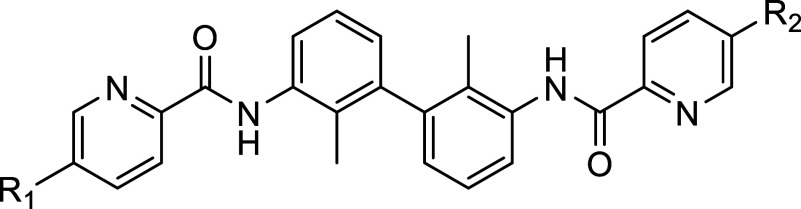

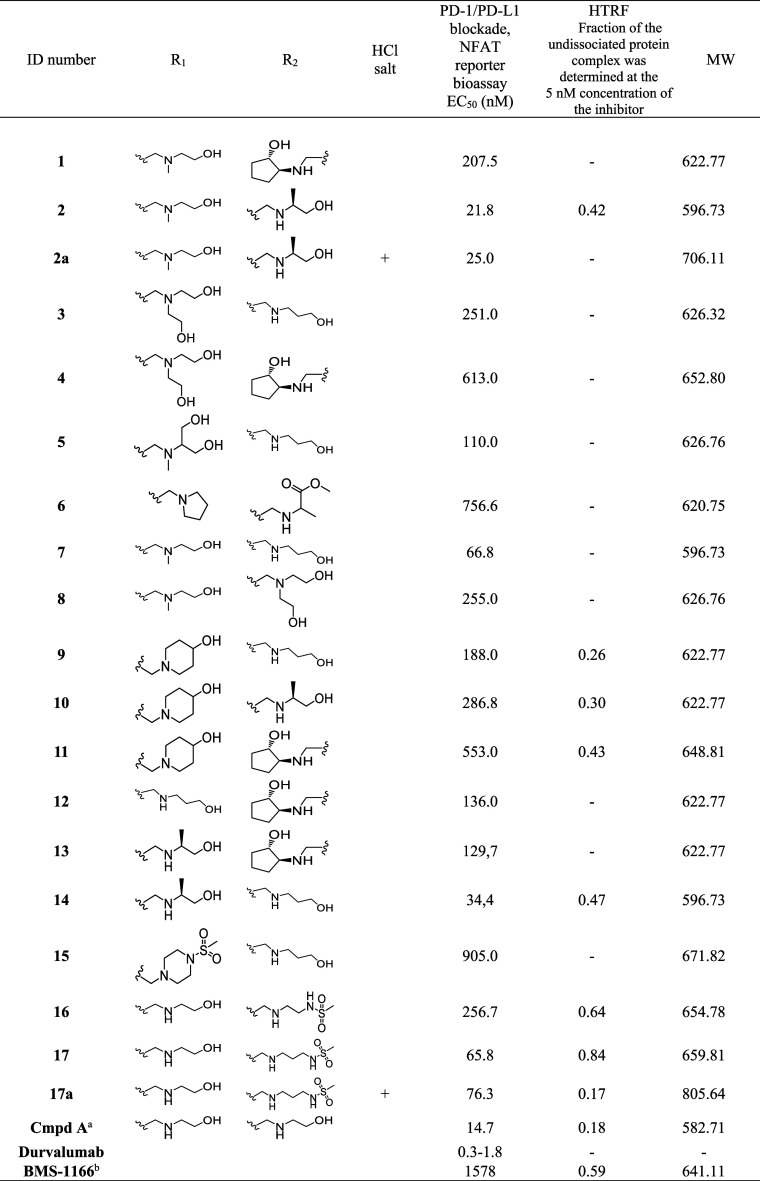
PD-1/PD-L1 Inhibitory Activity of the Compounds, wherein “Nonsymmetric” Means That R1 ≠ R2

a Compound A in Park et al.^25^ was used here as a reference compound. The EC_50_ of Compound A reported is 17 nM.^25^

b **BMS-1166** of BMS.^29,30^

A review of the literature suggests two distinct families of low-molecular-weight molecules that bind to PD-L1: (a) those derived from the biphenyl moiety (Figure S1),^23^ originally identified by researchers at Bristol-Myers Squibb (BMS) scientists,^28,29^ and (b) amino acid-based small molecules that mimic the PD-1/PD-L1 interface interaction.^15,24,30^ The detailed properties of the compounds based on biphenyl have been described in several publications.^22,23,31,32^ By analyzing these data together, we were able to divide the biphenyl core compounds into two pharmacophores. The first pharmacophore, termed “short,” consists of a core group, a linker, an aryl moiety, and a tail group (Figure S1A).^32,33^ The core is a biphenyl moiety that is located in a hydrophobic pocket composed of the amino acids Tyr56, Met115, and Ala121of PD-L1.^22,31^ The aryl group is typically a five- or six-membered aromatic ring or fused ring connected to the core group by a linker. The terminal tail is oriented toward the solvent region and could form interactions with nearby residues via hydrogen bonding. The second pharmacophore includes the compounds with the so-called C2 symmetry or pseudosymmetry, which contain polar groups at the termini (Figure S2).^34^ These compounds, referred to here as “long” or “C2,” have the same composition as the first pharmacophore but they have an additional linker, aryl, and tail group on the other side of the core moiety (Figure S2).^34^

NMR and X-ray cocrystal structure studies of small molecule/PD-L1 complexes showed that the C2 molecules, such as LH1307 (Figure S2B),^34^ form a more symmetric PD-L1 dimer than previously reported for the short inhibitors. The 2-(acetamido) ethylamine polar groups of BMS-202 incorporated into the C2 symmetric molecules of LH1307 are reported to extend from the hydrophobic cleft and occupy the solvent-exposed region sandwiched between the β strands of the PD-L1 dimer, thereby further enhancing binding to the two PD-L1 monomers.^34^ Another C2 compound induces a side chain flip of the Tyr56 protein residue to form a new cavity, which results in higher binding affinity to PD-L1 and higher PD-1/PD-L1 inhibitory activity under physiological conditions, as reported for Compound 4.^35^ Representative structures of compounds that have been disclosed by BMS, Incyte, Gilead, and others are shown in Figure S2B.

The C2-“long” compounds, themselves, can be symmetric or nonsymmetric depending on the linkers, aryl, and tail groups used. Almost all of these C2-long inhibitors found in the literature are symmetric.^15,23−25,34^ We decided to investigate the nonsymmetric C2-long inhibitors because the X-ray structures (PDB codes 5J89, 5J8O) and NMR studies suggest that a small-molecule molecule that binds to PD-L1 is located at the interface of the *asymmetric* PD-L1 dimer.^31,25,34^

The linkers of the compounds in Figure S1 and Figure S2 include the ether,^36^ alkene, and the amide moieties. Among these, the amide linker is a privileged linker given its druglike properties.^37,38^ The amide linker was first disclosed by Incyte and later on adopted by several other groups and companies (Figure S1 and Figure S2).^25^ In summary, our compounds are composed of a biphenyl in a core, an amide linker, and an arylpyridine group, and various tail solubilizing groups attached to the core (Table 1).

Three types of paths were used to obtain the final structures. These are described in Scheme 1, Scheme 2, and Scheme 3.

**Scheme 1:**
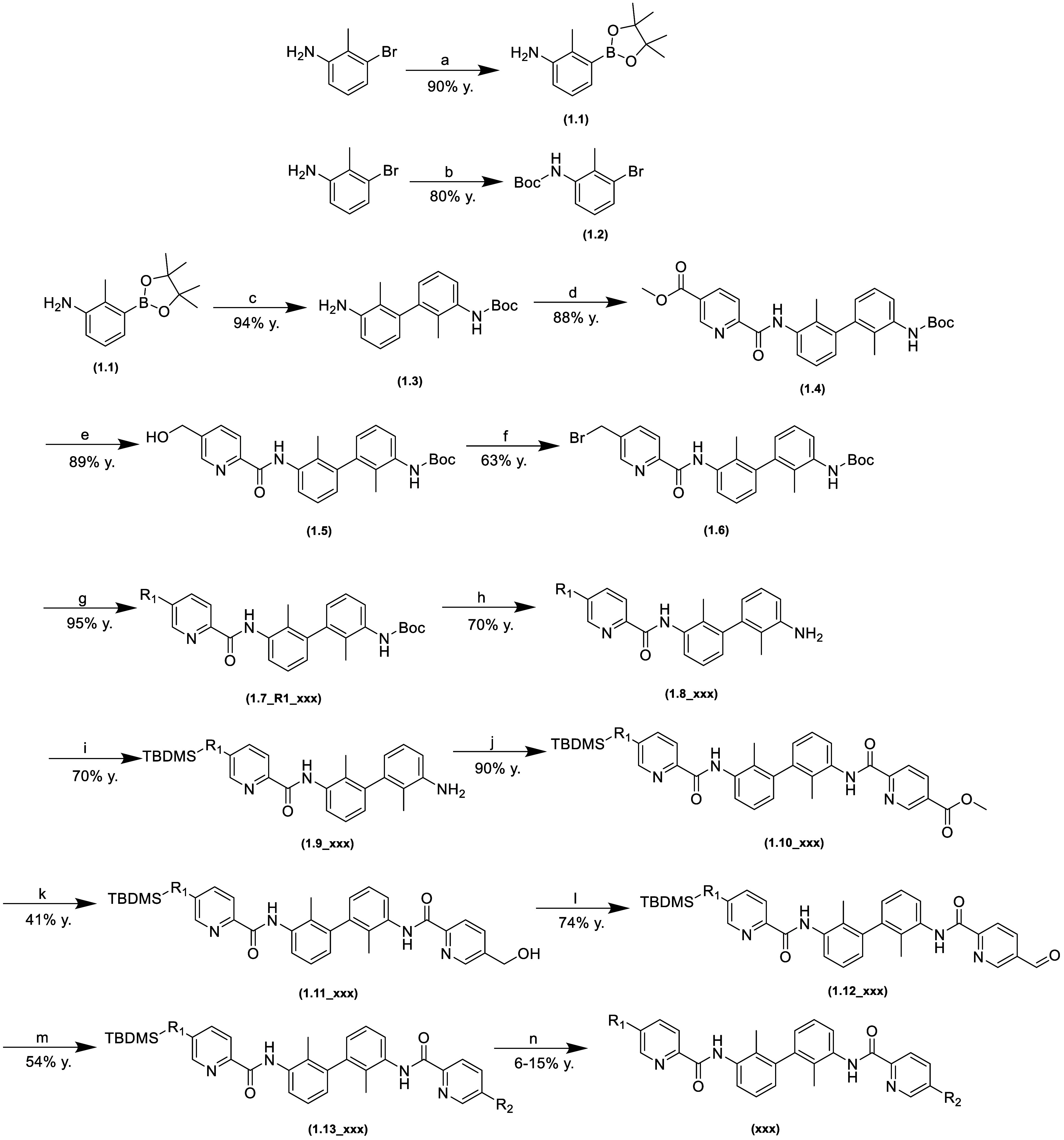
Synthesis of Nonsymmetric 1,1′-Biphenyl Derivatives (a) bis(pinacolato)diboron, Pd(dppf)Cl_2_·DCM, KOAc, 1,4-dioxane, 90 °C; (b) DIPEA, KI, Boc_2_O, ACN, 70 °C; (c) **1.2**, Pd(dppf)Cl_2_, K_2_CO_3_, 1,4-dioxane/H_2_O, 90 °C; (d) 5-(methoxycarbonyl)picolinic acid, HOBt, 1-ethyl-3-(3-dimethylaminopropyl)carbodiimide (EDC), DMF, 0 °C; (e) 4 M LiBH_4_ in THF, THF/MeOH, 0 °C; (f) *N*-Bromosuccinimide (NBS), PPh_3_, DCM; (g) K_2_CO_3_, KI, R_1_-amine, ACN, 50 °C; (h) 6 M HCl in i-PrOH, DCM; (i) imidazole, *tert*-butyldimethylsilyl chloride (TBDMSCl), DMF, 0 °C; (j) 5-(methoxycarbonyl)picolinic acid, HATU, DMF, 0 °C; (k) 4 M LiBH_4_, THF/MeOH, 0 °C; (l) Dess–Martin periodinane, DCM; (m) NaBH_3_CN, R_2_-amine, 1,2-dichloroethane (DCE)/MeOH; (n) 6 M HCl in i-PrOH, DCM.

**Scheme 2:**
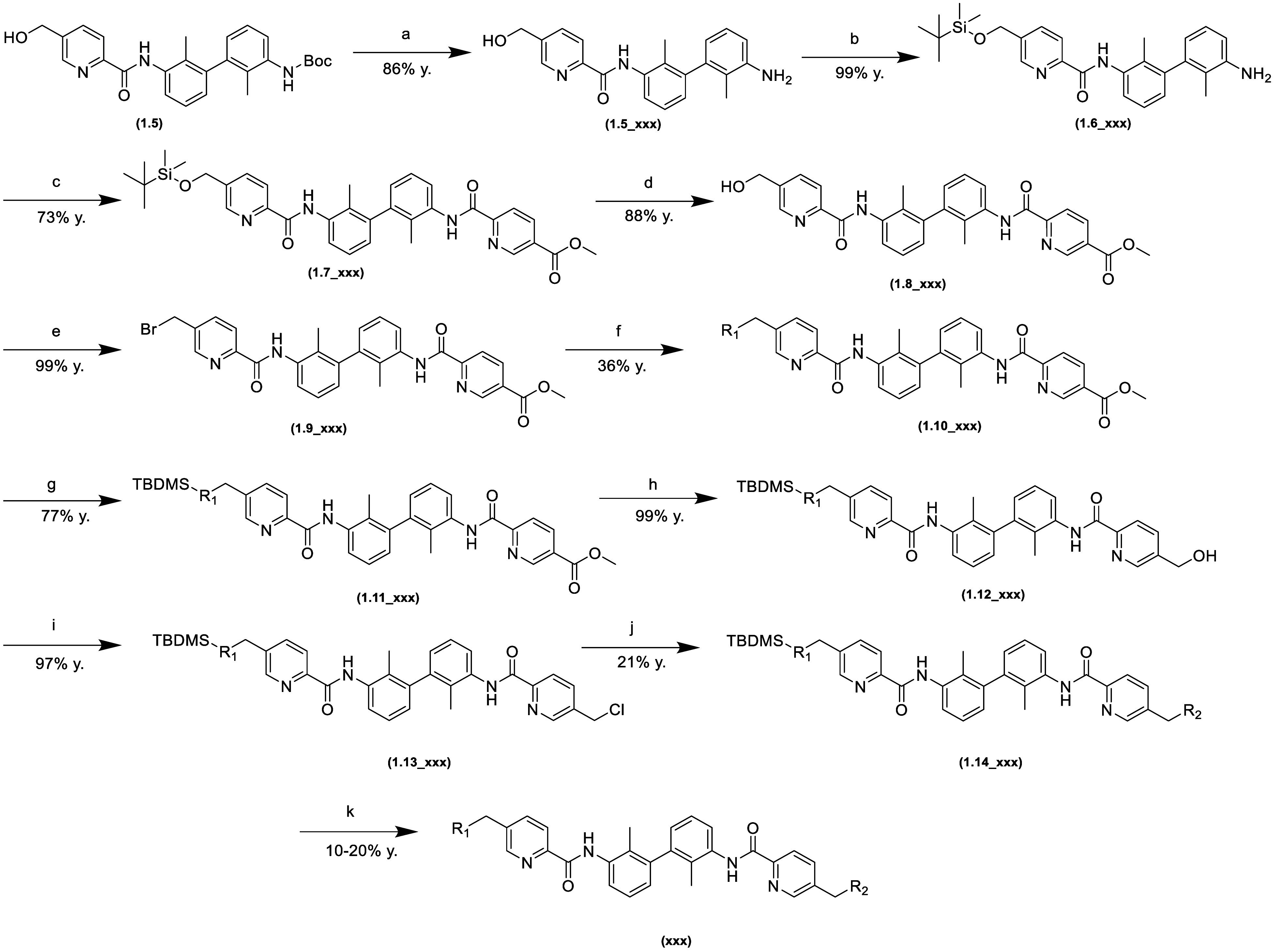
Synthesis Pathway of Nonsymmetric 1,1′-Biphenyl Derivatives (a) 6 M HCl in i-PrOH; (b) imidazole, TBDMSCl, DMF, 0 °C; (c) 5-(methoxycarbonyl)picolinic acid, HATU, TEA, DMF; (d) 6 M HCl in i-PrOH; (e) NBS, PPh_3_, DCM, 0 °C; (f) K_2_CO_3_, KI, R_1_-amine, ACN, 50 °C; (g) imidazole, TBDMSCl, DMF, 0 °C; (h) 4 M LiBH_4_ in THF, THF/MeOH, 0 °C; (i) SO_2_CH_3_Cl, TEA, DCM, 0 °C; (j) K_2_CO_3_, KI, R_2_-amine, ACN, 50 °C; (k) 6 M HCl in i-PrOH.

**Scheme 3:**
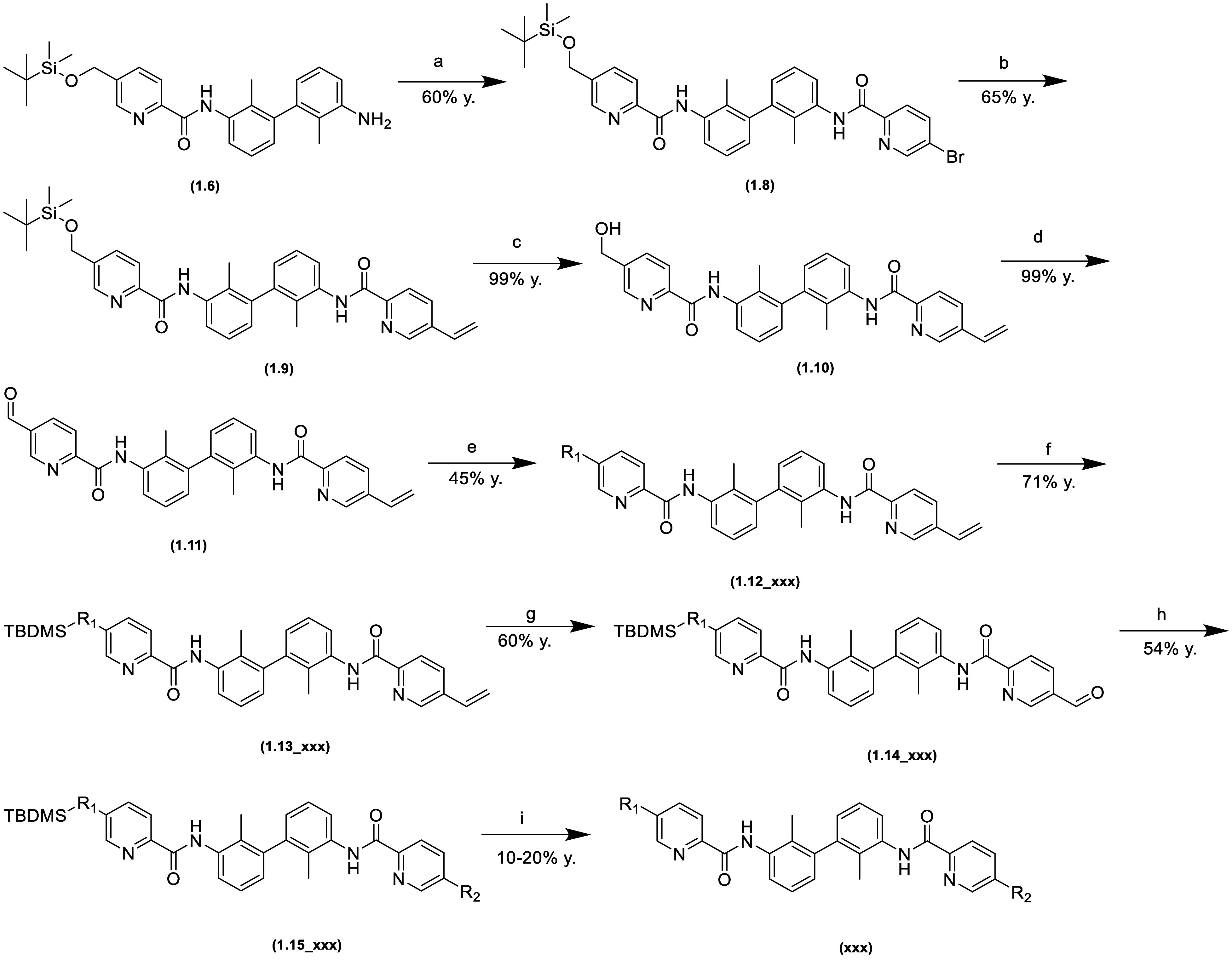
Synthesis Pathway of Nonsymmetric 1,1′-Biphenyl Derivatives (a) 5-(Methoxycarbonyl)picolinic acid, HATU, TEA, DMF; (b) CH_2_=CHBF_3_K, Pd(dppf)Cl_2_·DCM, K_2_CO_3_, 1,4-dioxane, H_2_O, 90 °C; (c) 6 M HCl in i-PrOH; (d) Dess–Martin, DCM; (e) K_2_CO_3_, KI, R_1_-amine, ACN, 50 °C; (f) imidazole, TBDMSCl, DMF, 0 °C; (g) Dess–Martin, DCM; (h) NaBH_3_CN, R_2_-amine, DCE, MeOH; (i) 6 M HCl in i-PrOH.

Schemes 1–3 present synthesis descriptions for a representative compound for each pathway. The term “**xxx**” refers to a specific final compound. The intermediates are additionally marked with the step number of each pathway.

In Scheme 1, in the first steps, we obtained the biphenyl core, which was synthesized in the Suzuki coupling of *tert*-butyl(3-bromo-2-methylphenyl)carbamate and 2-methyl-3-(4,4,5,5-tetramethyl-1,3,2-dioxaborolan-2-yl)aniline. Next, in the amide formation reaction with 5-(methoxycarbonyl)picolinic acid, we obtained an ester intermediate, which in further transformations gave a halide derivative. Then, in the S_N_2 reaction, we introduced the first solubilizer, R1, and then, after protection with *tert*-butyldimethylsilyl chloride and the second formation of the amide, we introduced the aldehyde, which in the reductive amidation reaction allows the entry of the second solubilizer—R2. The route described in Scheme 2 differs from the previous one in that both amide groups are introduced sequentially, distinguishing the sides of the molecule by protecting one hydroxyl group. The solubilizers were then introduced via S_N_2 reactions.

For the pathway shown in Scheme 3, another intermediate was used in the second amide formation reaction: 5-bromopicolinic acid. The bromine derivative was converted to vinyl by Suzuki–Miyaura cross-coupling reactions of potassium vinyl trifluoroborate. The vinyl was then converted to an aldehyde for reductive amidation. During the synthesis, modifications were made to the synthetic pathways to improve efficiency.

The synthesized inhibitors were evaluated by HTRF (homogeneous time-resolved fluorescence assay; Figure S3A), which allows for the determination of the range of inhibition of the compounds on the interaction of PD-1 with PD-L1 (Table 1). The compounds were further checked for their reactivation of PD-L1-blocked effector T cells using the cell-based immune checkpoint blockade (ICB) assay [EC_50_ (half-maximal effective concentration) values in Table 1, Figure S3B]. The results show that the type of solubilizer used influences the affinity of the target protein. Alkyl alcohol-solubilizing groups, e.g., derivatives of ethanolamine, generally perform better in the ICB cell-based assay than their cyclic counterparts, such as hydroxycyclopentyl.

**BMS-1166** was used as a reference compound in the HTRF measurements. As can be seen in Table 1, only two compounds showed lower activity in HTRF than reference **BMS-1166**. The rest of the synthesized compounds showed a lower amount of undissociated protein complex at 5 nM inhibitor concentration. A total of 83% of dissociation of the PD-1/PD L1 complex was achieved using 2-(ethylamino)ethan-1-ol and *N*-[3-(ethylamino)propyl]methanesulfonamide (**17a**) as a solubilizer. Interestingly, this compound is an HCl salt of **17** where we observed only 17% of the undissociated complex in the HTRF assay, which proves that the presence of the hydrochloric salt increases the solubility of the compound (Table S2). A similar trend was observed for **2** and its HCl salt **2a** (Table S2).

Good results can also be seen in the Promega cell assay. Compound A,^25,39^ a resynthesized compound from a patent with symmetric analogues,^39^ was used as a reference and showed activity in our cell assay with an EC_50_ of 14.7 nM.

The use of linear solubilizing groups such as (*S*)-2-(methylamino)propan-1-ol, 2-(dimethylamino)ethan-1-ol, or 3-(methylamino)propan-1-ol, resulted in EC_50_ values in the range of 100 nM and lower. The inhibitors with a more branched moiety, such as diethanolamine or 2-(methylamino)propane-1,3-diol, showed activity around 200 nM (**3** and **5**). However, for compound **4** with additional (1*S*,2*S*)-2-aminocyclopentan-1-ol, the EC_50_ was above 2000 nM. The compound with a less polar solubilizing group—pyrrolidine and methyl ethyl alanine (**7**)—has no activity in the assay. EC_50_ values above 180 nM were observed for compounds with *N*-methyl-4-piperidinol and 1-ethyl-4-(methylsulfonyl)piperazine as solubilizing tags (**9**, **10**, **11**, and **15**).

Representative molecules were evaluated for their interaction with PD-L1 by means of an ^1^H NMR titration experiment. NMR showed that the compounds induced oligomerization of the protein (Figure 1). Upon addition of compounds **14**, **17**, and **17a** to hPD-L1 (human PD-L1), the proton NMR lines of hPD-L1 broaden, thereby indicating oligomerization of hPD-L1, which we have previously observed with similar compounds.^31,40,41^

**Figure 1:**
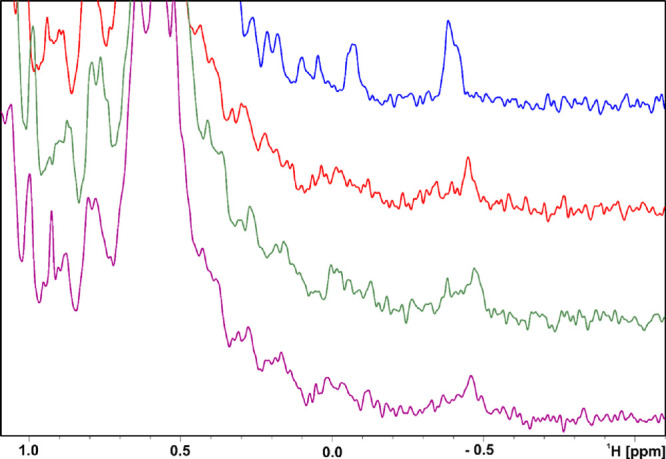
Aliphatic part of ^1^H NMR spectra of hPD-L1 (blue), hPD-L1 with **14** (red), **17** (green), and **17a** (purple) in the molar ratio protein to the compound 1:1, respectively.

In addition, we performed a PD-1/PD-L1 NMR-based antagonist-induced dissociation assay (AIDA).^42,43^ The AIDA assay was performed by adding a slight molar excess of PD-L1 to PD-1, which causes ^1^H NMR signals to broaden corresponding to complex formation (Figure 2). The addition of compounds that displace PD-1 from PD-L1 results in rescue of the PD-1 signal. There is a 100% rescue percentage for **17a**, which indicates that **17a** was able to completely displace PD-1.The spectra in Figure 2 (purple) and Figure 1 (purple) match those in the broader signals of PD-L1. However, in Figure 2 (purple), these are obscured by the sharper NMR signals of monomeric PD-1 released from the PD-L1/PD-1 complex. Hence, the similarity of the purple spectrum (bottom) is the upper blue spectrum in Figure 2.

**Figure 2:**
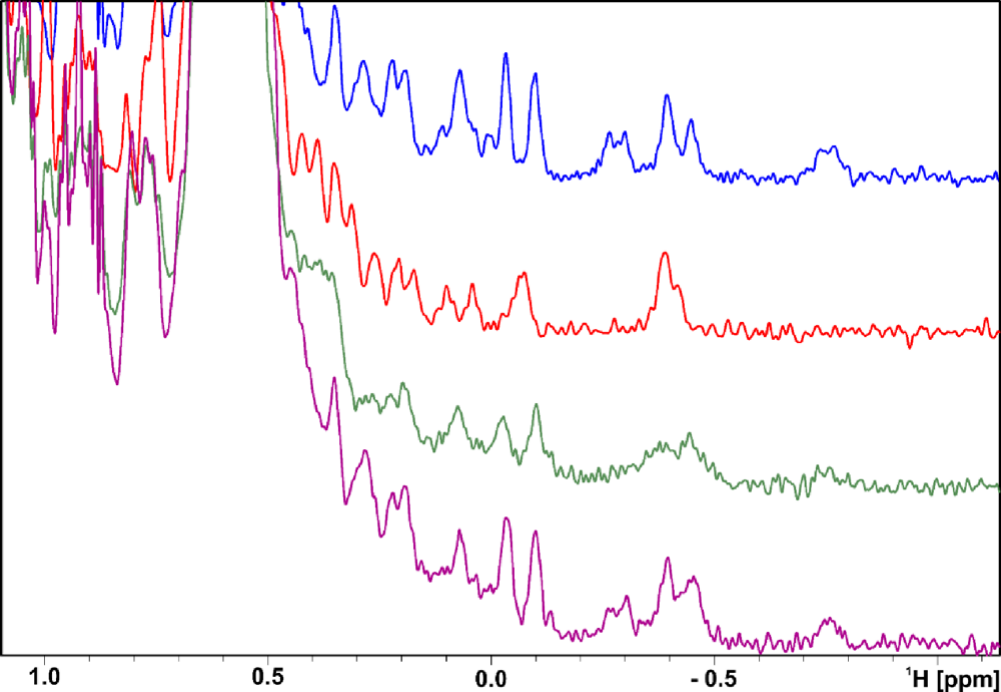
NMR-based AIDA assay showing that a compound displaces PD-1 from PD-L1. The aliphatic part of the ^1^H NMR spectra of hPD-1 (blue), hPD-L1 (red), the complex of hPD-1/hPD-L1 (green), and the PD-1 signals at −0.4 ppm (green) are broadened by the addition of PD-L1 (red) compared with PD-1 alone (blue). The complex of hPD-1/hPD-L1 with **17a** (purple) in a 1:1 molar ratio of protein to compound 1:1. Rescue of the signal intensity by addition of **17a** to the complex (purple) suggests that the compound displaces PD-1 from PD-L1.

## Structural Studies of the **17a** Interactions with PD-L1

**Figure 3:**
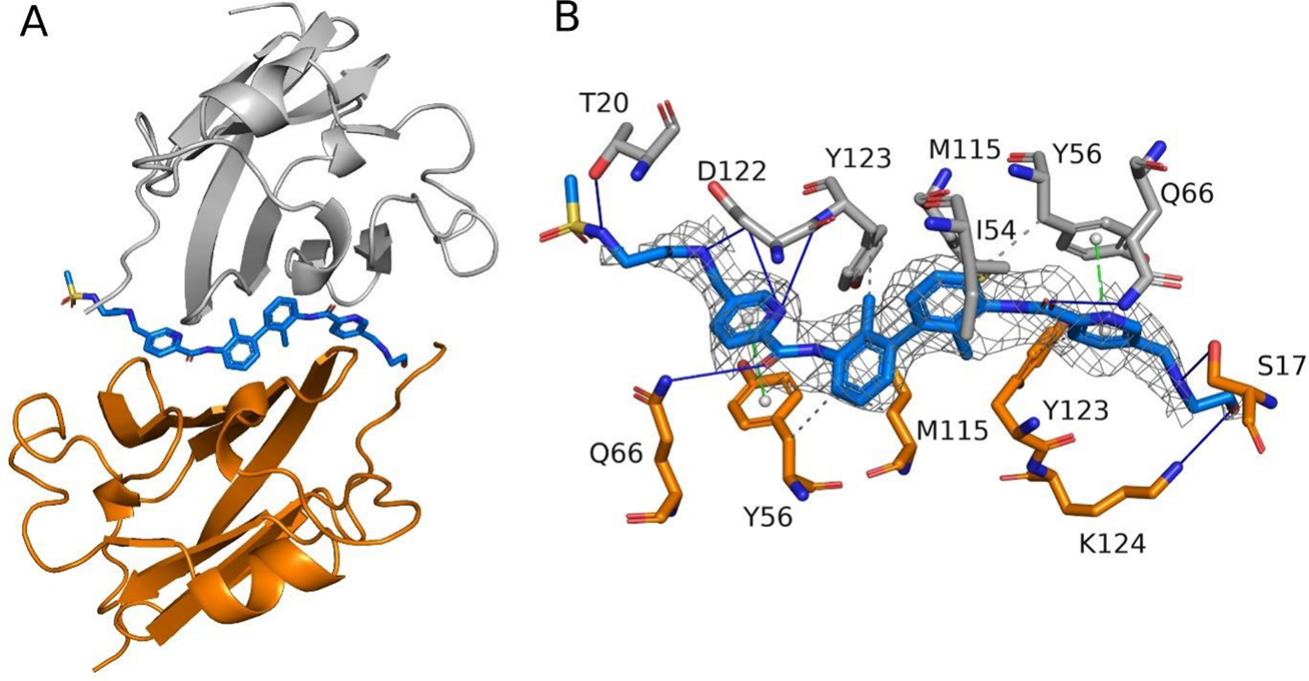
X-ray structure of **17a** in complex with hPD-L1 (PDB: 9EO0). (A) Arrangement of one of the three dimers in the asymmetric unit. Two PD-L1 molecules with chain A in gray and B in orange form an interface with **17a** represented as blue sticks. (B) Detailed interactions of **17a** with PD-L1 dimer. The inhibitor interacts with PD-L1 to induce its dimerization. Color coding is as in (A). The electron density of **17a** (2Fo-Fc map at 1 σ) represented as a gray isomesh.

We managed to cocrystallize PD-L1 with compound **17a** and solve the structure at the resolution of 2.5 Å (Figure 3 and Table S1). Attempts to crystallize **2** and **2a** failed to produce suitable diffracting crystals. The asymmetric unit of the PD-L1 costructure with **17a** with the *P*12_1_1 space group contains six chains arranged in three dimers bridged by the inhibitor found at the dimer interface. The **17a** electron density was well-defined at the PD-L1 dimer interface with the exception of sulfonamide solubilizer at the R2 position (Figure 3B). The inhibitor pose on PD-L1 dimer interface resembles those reported for C_2_-symmetric inhibitors with highly symmetrical hydrophobic interactions, including π–π stacking between pyridine rings and _A,B_Tyr56, as well as π–alkyl interactions with _A,B_Ile54, _A,B_Met115, and _A,B_Tyr123 (comparison between **17a** pose and C2-symmetric anti-PD-L1 compound PDB ID: 6RPG in Supplementary Figure S4).^34^ However, the positioning of distal phenyl rings are different, likely because of different linkers between biphenyl core and more branched substitution in the case of PDB ID: 6PRG. Moreover, numerous symmetrical hydrogen bonds are also reported, such as between amide carboxylate linkers and _A,B_Met115, or nonsymmetric with _A_Lys124 or _B_Asp122. Sulfonamide polar group, although smilingly stabilized by hydrogen interactions with _B_Thr20, is not resolved well in the electron density and is mainly water-exposed outside of the “hydrophobic tunnel” between PD-L1 monomers, likely contributing to the solubility of the **17a**.

Our work provides the characterization of low-molecular-weight C2 nonsymmetric binders to PD-L1 obtained by providing the central biphenyl moiety on either side with the amide linkers attached to an aryl ring and a tail group that are different on these two sides (Table 1). Similar C2-long compounds have been reported in the scientific and patent literature. However, the first group is based on an ether linker (i.e., different from the amide linker), and these compounds are rather weak inhibitors.^34,35^ For the C2 compounds with the amide linker, the solubilizing groups were identical at both ends of the molecules,^25,37^ and the majority of the reports included only the in vitro HTRF binding data and did not provide the activity in an ex vivo cellular environment.^37,39^ HTRF assay measures compound activity in PD-1/PD-L1 complex dissociation. Many potent compounds in the HTRF assay have little or even no activity in the cellular context; see, for example, Konieczny et al.^44^ where the “best” compound there for PD-L1 had an excellent HTRF IC_50_ of 14.9 nM but a poor EC_50_ activity in the PD-1/PD-L1 cells of only 6632 nM.

In conclusion, the compounds described in this study are potent antagonists of PD-1/PD-L1 complexation, as seen in conventional in vitro assays, such as NMR and HTRF and, importantly, the NFAT assay. Activation of Jurat cells by our “best” compounds was at EC_50_ levels approaching those of the control antibodies despite the fact that they were significantly smaller in size. Like other PD-L1 binders described in the literature,^31^ our compounds are dimerizers of human PD-L1. Their specificity is to hPD-L1. In the NFAT assay, the compounds had an EC_50_ (half-maximal effective concentration) below 1000 nM, with 79% of the compounds having an EC_50_ cutoff below 300 nM (Table 1). Such data are not found in publications.^35−37^ The PD-1/PD-L1 inhibitors with an EC_50_ above this cutoff are not considered potent enough to stimulate T cells to clinically relevant anticancer activity. Our most potent compound, **2**, features exceptionally high target affinity and demonstrated potency in cell-based assays with an EC_50_ of 21.8 nM. This identifies **2** as an excellent candidate for further preclinical and clinical studies in anti-PD-L1 cancer therapy. The X-ray structure presented helps to explain the enhanced inhibitory activity of these inhibitors.

## Experimental Section

### General Chemistry

Syntheses were carried out following the procedures summarized in Scheme 1, Scheme 2, and Scheme 3. Additional information can be found in the Supporting Information.

UHPLC-MS/MS analyses were performed using a Waters ACQUITY UPLC instrument (Waters Corporation, Milford, MA, USA) coupled to a Waters TQD mass spectrometer. Chromatographic separation was performed using a 2.1 × 100 mm Acquity UPLC BEH C18 column with a particle size of 1.7 μm and equipped with a VanGuard Acquity UPLC BEH C18 column with a size of 2.1 × 5 mm and a particle size of 1.7 μm. The column was maintained at 40 °C and eluted with a gradient from 95% to 0% eluent A over 10 min and then isocratically using 100% eluent B over 2 min at a flow rate of 0.3 mL min^–1^. Eluent A was water/formic acid (0.1%, v/v); eluent B was acetonitrile/formic acid (0.1%, v/v). Diode array detector (DAD) chromatograms were recorded by using a Waters eλ PDA detector. The spectra were analyzed in the range of 200–700 nm with a resolution of 1.2 nm and a sampling rate of 20 points s^–1^. The MS analysis parameters were as follows: source temperature 150 °C, desolvation temperature 350 °C, desolvation gas flow 600 L h^–1^, shield gas flow 100 L h^–1^, capillary potential 3.00 kV, and cone potential 30 V. Gas nitrogen was used for nebulization and desolvation. Data was collected in the range from 50 to 1000 *m*/*z* in intervals of 0.5 s.

### Homogeneous Time-Resolved Fluorescence

The certified CisBio kit was used to conduct the HTRF assay, adhering to the manufacturer’s guidelines. The assay setup comprised hPD-1, hPD-L1, anti-Tag1 tagged with Europium cryptate (HTRF donor), and anti-Tag2 tagged with XL665 (HTRF acceptor). The experiments utilized final concentrations of 5 nM hPD-L1 and 50 nM hPD-1 in a 20 μL total volume performed in triplicate. Per the CisBio instructions, all components were combined, the plate was incubated at ambient temperature for 2 h, and the TR-FRET (time-resolved fluorescence energy transfer) was carried out using the Tecan Spark 20M. The negatives in the acquired data were subtracted for background, the positives were standardized, and then the data were averaged.

### PD-1/PD-L1 Blockade Bioassay

The PD-1/PD-L1 immune checkpoint bioassay (PD-1/PD-L1 Bioassay, Promega) was conducted according to the manufacturer’s protocol. PD-L1+ aAPC/CHO-K1 cells were seeded at a density of 40 × 10^4^ cells in 100 μL of medium [Ham’s F12, 10% fetal bovine serum (FBS)] in 96-well white flat-bottomed plates and incubated overnight at 37 °C in the presence of 5% CO_2_. The next day, the medium was removed, and serial dilutions of small molecule inhibitors were added at 40 μL per well in assay buffer (RPMI1640 + 1% FBS + 1% DMSO). PD-1 effector Jurkat cells resuspended in assay buffer (RPMI 1640 + 1% FBS) at 1.25 × 10^6^ cells/mL were then added at 40 μL per well (total of 50 × 10^4^ cells). The cells were cocultured for 6 h at 37 °C with 5% CO_2_ followed by a 5 min equilibration at room temperature. Bio-Glo Reagent (Promega) was prepared and added at 80 μL per well. After a 15 min incubation at room temperature, luminescence was measured using a Tecan Spark microplate reader. Data are presented as fold induction of luminescence relative to DMSO-treated cells. EC_50_ values were calculated using the log(inhibitor) vs response variable slope (four-parameter) analysis model with GraphPad Prism software.

### Protein Expression and Purification

The human PD-L1 IgV domain (residues 18–134, C-terminal His-tag) was expressed and purified following previously described methods.^21,22^ Briefly, hPD-L1 was produced in *Escherichia coli* BL21 as inclusion bodies, which were collected, washed, and dissolved in a guanidine buffer (6 M guanidine·HCl, 50 mM Tris pH 8, 200 mM NaCl, 2 mM EDTA, and 10 mM β-mercaptoethanol). Refolding of PD-L1 was achieved by dropwise dilution into a refolding buffer (0.1 M Tris, pH 8.0, 1 M l-Arg hydrochloride, 2 mM EDTA, 0.25 mM oxidized glutathione, and 0.25 mM reduced glutathione). The refolded PD-L1 was dialyzed three times against 10 mM Tris, pH 8.0, with 20 mM NaCl and then concentrated and purified using size exclusion chromatography on a Superdex 75 column (GE Healthcare) in 10 mM Tris buffer, pH 8.0, with 20 mM NaCl.

## Supporting Information Available

Comparison of the biphenyl-based inhibitor structures. Experimental procedures for substrate preparation; results of the PD-1/PD-L1 blockade bioassay assay; X-ray data collection and refinement statistics (molecular replacement) for the PD-L1 cocrystal structure with **17a**; results of the ICB assay; compound purity and NMR data (PDF)
Strings of the molecular formula (CSV)

## Accession Codes

The structure factors and final models of PD-L1 complexes with inhibitor **17a** have been deposited in the Protein Data Bank under accession number 9EO0.

## References

[ref1] Hoos A. Development of Immuno-Oncology Drugs-from CTLA4 to PD1 to the next Generations. Nat. Rev. Drug Discovery 2016, 15, 235–247. 10.1038/nrd.2015.35. 26965203

[ref2] Khalil D. N.; Smith E.; Brentjens R.; Wolchok J. D. The Future of Cancer Treatment: Immunomodulation, CARs and Combination Immunotherapy. Nat. Rev. Clin. Oncol. 2016, 13, 273–290. 10.1038/nrclinonc.2016.25. 26977780 PMC5551685

[ref3] Domling A.; Holak T. A. Programmed death-1: therapeutic success after more than 100 years of cancer immunotherapy. Angew. Chem., Int. Ed. Engl. 2014, 53, 2286–2288. 10.1002/anie.201307906. 24474145 PMC4104593

[ref4] Mahoney K. M.; Rennert P. D.; Freeman G. J. Combination Cancer Immunotherapy and New Immunomodulatory Targets. Nat. Rev. Drug Discovery 2015, 14, 561–584. 10.1038/nrd4591. 26228759

[ref5] Ribas A.; Wolchok J. D. Cancer Immunotherapy Using Checkpoint Blockade. Science 2018, 359, 1350–1355. 10.1126/science.aar4060. 29567705 PMC7391259

[ref6] Sharma P.; Allison J. P. The Future of Immune Checkpoint Therapy. Science 2015, 348, 56–61. 10.1126/science.aaa8172. 25838373

[ref7] Sharma P.; Allison J. P. Dissecting the Mechanisms of Immune Checkpoint Therapy. Nat. Rev. Immunol. 2020, 20, 75–76. 10.1038/s41577-020-0275-8. 31925406

[ref8] Chow A.; Perica K.; Klebanoff C. A.; Wolchok J. D. Clinical implications of T cell exhaustion for cancer immunotherapy. Nat. Rev. Clin. Oncol. 2022, 19, 775–790. 10.1038/s41571-022-00689-z. 36216928 PMC10984554

[ref9] Sharma P.; Siddiqui B. A.; Anandhan S.; Yadav S. S.; Subudhi S. K.; Gao J.; Goswami S.; Allison J. P. The Next Decade of Immune Checkpoint Therapy. Cancer, Discovery 2021, 11, 838–857. 10.1158/2159-8290.CD-20-1680. 33811120

[ref10] Chamoto K.; Hatae R.; Honjo T. Current Issues and Perspectives in PD-1 Blockade Cancer Immunotherapy. Int. J. Clin. Oncol. 2020, 25, 790–800. 10.1007/s10147-019-01588-7. 31900651 PMC7192862

[ref11] Chamoto K.; Yaguchi T.; Tajima M.; Honjo T. Insights from a 30-year journey: function, regulation and therapeutic modulation of PD1. Nat. Rev. Immunol. 2023, 23, 682–695. 10.1038/s41577-023-00867-9. 37185300

[ref12] Pauken K. E.; Torchia J. A.; Chaudhri A.; Sharpe A. H.; Freeman G. J. Emerging concepts in PD-1 checkpoint biology. Semin Immunol. 2021, 52, 101480 10.1016/j.smim.2021.101480. 34006473 PMC8545711

[ref13] Baldo B. Adverse Events to Monoclonal Antibodies Used for Cancer Therapy: Focus on Hypersensitivity Responses. Oncoimmunol. 2013, 2, e26333 10.4161/onci.26333. PMC3827071 24251081

[ref14] Farid S. S. Process Economics of Industrial Monoclonal Antibody Manufacture. J. Chromatogr. B 2007, 848, 8–18. 10.1016/j.jchromb.2006.07.037. 16899415

[ref15] Sasikumar P. G.; Ramachandra M. Small Molecule AgentsTargeting PD-1 Checkpoint Pathway for Cancer Immunotherapy: Mechanisms of Action and Other Considerations for Their Advanced Development. Front. Immunol. 2022, 13, 752065 10.3389/fimmu.2022.752065. 35585982 PMC9108255

[ref16] Imai K.; Takaoka A. Comparing antibody and small-molecule therapies for cancer. Nat. Rev. Cancer. 2006, 6, 714–27. 10.1038/nrc1913. 16929325

[ref17] Adams J. L.; Smothers J.; Srinivasan R.; Hoos A. Big Opportunities for Small Molecules in Immuno-Oncology. Nat. Rev. Drug Discovery 2015, 14, 603–622. 10.1038/nrd4596. 26228631

[ref18] Huck B. R.; Kötzner L.; Urbahns K. Small Molecules Drive Big Improvements in Immuno-Oncology Therapies. Angew. Chemie Int. Ed. 2018, 57, 4412–4428. 10.1002/anie.201707816. PMC5900885 28971564

[ref19] Scott D. E.; Bayly A. R.; Abell C.; Skidmore J. Small Molecules, Big Targets: Drug Discovery Faces the Protein–Protein Interaction Challenge. Nat. Rev. Drug Disc. 2016, 15, 533–550. 10.1038/nrd.2016.29. 27050677

[ref20] Wells J. A.; McClendon C. L. Reaching for High-Hanging Fruit in Drug Discovery at Protein-Protein Interfaces. Nature 2007, 450, 1001–1009. 10.1038/nature06526. 18075579

[ref21] Zak K. M.; Kitel R.; Przetocka S.; Golik P.; Guzik K.; Musielak B.; Dömling A.; Dubin G.; Holak T. A. Structure of the Complex of Human Programmed Death 1, PD-1, and Its Ligand PD-L1. Structure 2015, 23, 2341–2348. 10.1016/j.str.2015.09.010. 26602187 PMC4752817

[ref22] Zak K. M.; Grudnik P.; Guzik K.; Zieba B. J.; Musielak B.; Dömling A.; Dubin G.; Holak T. A. Structural Basis for Small Molecule Targeting of the Programmed Death Ligand 1 (PD-L1). Oncotarget 2016, 7, 30323–30335. 10.18632/oncotarget.8730. 27083005 PMC5058683

[ref23] Kitel R.; Rodríguez I.; del Corte X.; Atmaj J.; Żarnik M.; Surmiak E.; Muszak D.; Magiera-Mularz K.; Popowicz G. M.; Holak T. A.; Musielak B. Exploring the Surface of the Ectodomain of the PD-L1 Immune Checkpoint with Small-Molecule Fragments. ACS Chem. Biol. 2022, 17, 2655–2663. 10.1021/acschembio.2c00583. 36073782 PMC9486809

[ref24] Guzik K.; Tomala M.; Muszak D.; Konieczny M.; Hec A.; Błaszkiewicz U.; Pustuła M.; Butera R.; Dömling A.; Holak T. A. Development of the Inhibitors That Target the PD-1/PD-L1 Interaction—a Brief Look at Progress on Small Molecules, Peptides and Macrocycles. Molecules 2019, 24, 2071 10.3390/molecules24112071. 31151293 PMC6600339

[ref25] Park J.-J.; Thi E. P.; Carpio V. H.; Bi Y.; Cole A. G.; Dorsey B. D.; Fan K.; Harasym T.; Iott C. L.; Kadhim S.; Kim J. H.; Lee A. C. H.; Nguyen D.; Paratala B. S.; Qiu R.; White A.; Lakshminarasimhan D.; Leo C.; Suto R. K.; Rijnbrand R.; Tang S.; Sofia M. J.; Moore C. B. Checkpoint Inhibition through Small Molecule-Induced Internalization of Programmed Death-Ligand 1. Nat. Commun. 2021, 12, 1222 10.1038/s41467-021-21410-1. 33619272 PMC7900207

[ref26] Koblish H. K.; Wu L.; Wang L. S.; Liu P. C. C.; Wynn R.; Rios-Doria J.; Spitz S.; Liu H.; Volgina A.; Zolotarjova N.; Kapilashrami K.; Behshad E.; Covington M.; Yang Y. O.; Li J.; Diamond S.; Soloviev M.; O’Hayer K.; Rubin S.; Kanellopoulou C.; Yang G.; Rupar M.; DiMatteo D.; Lin L.; Stevens C.; Zhang Y.; Thekkat P.; Geschwindt R.; Marando C.; Yeleswaram S.; Jackson J.; Scherle P.; Huber R.; Yao W.; Hollis G. Characterization of INCB086550: A potent and novel smallmolecule PD-L1 inhibitor. Cancer Discovery 2022, 12 (6), 1482–1499. 10.1158/2159-8290.CD-21-1156. 35254416 PMC9394386

[ref27] Musielak B.; Kocik J.; Skalniak L.; Magiera-Mularz K.; Sala D.; Czub M.; Stec M.; Siedlar M.; Holak T. A.; Plewka J. CA-170 - A Potent Small-Molecule PD-L1 Inhibitor or Not?. Molecules 2019, 24, 2804 10.3390/molecules24152804. 31374878 PMC6695792

[ref28] Chupak L.; Ding M.; Martin S.; Zheng X.; Hewawasam P.; Connoly T.; Xu N.; Yeung K.; Zhu J.; Langley D.; Tenney D.; Scola P. Compounds Useful as Immunomodulators. WO 2015/160641 A2, 2015.

[ref29] Chupak L. S.; Zheng X. Compounds Useful as Immunomodulators. WO 2015/034820 A1, 2015.

[ref30] Surmiak E.; Magiera-Mularz K.; Musielak B.; Muszak D.; Kocik-Krol J.; Kitel R.; Plewka J.; Holak T. A.; Skalniak L. PD-L1 Inhibitors: Different Classes, Activities, and Mechanisms of Action. Int. J. Mol. Sci. 2021, 22, 11797 10.3390/ijms222111797. 34769226 PMC8583776

[ref31] Guzik K.; Zak K. M.; Grudnik P.; Magiera K.; Musielak B.; Törner R.; Skalniak L.; Dömling A.; Dubin G.; Holak T. A. Small- Molecule Inhibitors of the Programmed Cell Death-1/Programmed Death-Ligand 1 (PD-1/PD-L1) Interaction via Transiently Induced Protein States and Dimerization of PD-L1. J. Med. Chem. 2017, 60, 5857–5867. 10.1021/acs.jmedchem.7b00293. 28613862

[ref32] Deng J.; Cheng Z.; Long J.; Dömling A.; Tortorella M.; Wang Y. Small Molecule Inhibitors of Programmed Cell Death Ligand 1 (PD-L1): A Patent Review (2019–2021). Expert. Opin. Ther. Pat. 2022, 32, 575–589. 10.1080/13543776.2022.2045276. 35272536

[ref33] Yang J.; Basu S.; Hu L. Design, synthesis, and structure–activity relationships of 1,2,3,4-tetrahydroisoquinoline-3-carboxylic acid derivatives as inhibitors of the programmed cell death-1 (PD-1)/programmed cell death-ligand 1 (PD-L1) immune checkpoint pathway. Med. Chem. Res. 2022, 31, 1716–1739. 10.1007/s00044-022-02926-7.

[ref34] Basu S.; Yang J.; Xu B.; Magiera-Mularz K.; Skalniak L.; Musielak B.; Kholodovych V.; Holak T. A.; Hu L. Design, Synthesis, Evaluation, and Structural Studies of C2-Symmetric Small Molecule Inhibitors of Programmed Cell Death-1/Programmed Death-Ligand 1 Protein–Protein Interaction. J. Med. Chem. 2019, 62 (15), 7250–635. 10.1021/acs.jmedchem.9b00795. 31298541

[ref35] Kawashita S.; Aoyagi K.; Yamanaka H.; Hantani R.; Naruoka S.; Tanimoto A.; Hori Y.; Toyonaga Y.; Fukushima K.; Miyazaki S.; Hantani Y. Symmetry-Based Ligand Design and Evaluation of Small Molecule Inhibitors of Programmed Cell Death-1/Programmed Death-Ligand 1 Interaction. Bioorg. Med. Chem. Lett. 2019, 29 (17), 2464–2467. 10.1016/j.bmcl.2019.07.027. 31351692

[ref36] Wang M. Symmetric or Semi-symmetric Compounds Useful as Immunomodulators. WO2018/026971 A1, 2018.

[ref37] Wu L.; Yu Z.; Zhang F.; Yao W. Pridine derivatives as immunomodulators. WO 2018119221 Al, 2018.

[ref38] Yu Z.; Wu L.; Yao W. Heterocyclic compounds as immunomodulators. US20200283423A1, 2020.

[ref39] Bi Y.; Dorsey B. D.; Fan Y.; Brooks Moore C.; Nguyen D. Substituted 1,1′-Biphenyl Compounds, Analogues Thereof, and Methods Using Same. WO2019191624A1, 2019.

[ref40] Muszak D.; Surmiak E.; Plewka J.; Magiera-Mularz K.; Kocik-Krol J.; Musielak B.; Sala D.; Kitel R.; Stec M.; Weglarczyk K.; Siedlar M.; Domling A.; Skalniak L.; Holak T. A. Terphenyl-Based Small-Molecule Inhibitors of Programmed Cell Death-1/Programmed Death-Ligand 1 Protein–Protein Interaction. J. Med. Chem. 2021, 64, 11614–11636. 10.1021/acs.jmedchem.1c00957. 34313116 PMC8365601

[ref41] Skalniak L.; Zak K. M.; Guzik K.; Magiera K.; Musielak B.; Pachota M.; Szelazek B.; Kocik J.; Grudnik P.; Tomala M.; Krzanik S.; Pyrc K.; Dömling A.; Dubin G.; Holak T. A. Small-Molecule Inhibitors of PD-1/PD-L1 Immune Checkpoint Alleviate the PD-L1- Induced Exhaustion of T-Cells. Oncotarget 2017, 8, 72167–72181. 10.18632/oncotarget.20050. 29069777 PMC5641120

[ref42] D’Silva L.; Ozdowy P.; Krajewski M.; Rothweiler U.; Singh M.; Holak T. A. Monitoring the effects of antagonists on protein-protein interactions with NMR spectroscopy. J. Am. Chem. Soc. 2005, 127, 13220–13226. 10.1021/ja052143x. 16173750

[ref43] Krajewski M.; Rothweiler U.; D’Silva L.; Majumdar S.; Klein C.; Holak T. A. An NMR-based antagonist induced dissociation assay for targeting the ligand-protein and protein-protein interactions in competition binding experiments. J. Med. Chem. 2007, 50, 4382–4387. 10.1021/jm070365v. 17696513

[ref44] Konieczny M.; Musielak B.; Kocik J.; Skalniak L.; Sala D.; Czub M.; Magiera-Mularz K.; Rodriguez I.; Myrcha M.; Stec M.; Siedlar M.; Holak T. A.; Plewka J. Di-bromo-Based Small-Molecule Inhibitors of the PD-1/PD-L1 Immune Checkpoint. J. Med. Chem. 2020, 63, 11271–11285. 10.1021/acs.jmedchem.0c01260. 32936638 PMC7584369

