# Supplementary material for: Nonsymmetrically Substituted 1,1′-Biphenyl-Based Small Molecule Inhibitors of the PD-1/PD-L1 Interaction
Source: ACS Med Chem Lett. 2024 Jun 3;15(6):828–36. doi: 10.1021/acsmedchemlett.4c00042 (PMC11181486; doi:10.1021/acsmedchemlett.4c00042)
Supplement: Supplementary file 1 — ml4c00042_si_004.pdf [file ml4c00042_si_004.pdf]

# Supporting information

## Nonsymmetrically Substituted 1,1'-Biphenyl-Based Small-Molecule Inhibitors of the PD-1/PD-L1 Interaction

Aleksandra Hec-Gałązka,[1,2,3] Urszula Tyrcha,[3] Jan Barczyński,[3] Przemyslaw Bielski,[1,2,3] Michał Mikitiuk,[3] Ganna P. Gudz,[2] Radosław Kitel,[2] Bogdan Musielak,[2] Jacek Plewka,[2] Tomasz Sitar,[3*] Tad A. Holak.[3*]

[1]Jagiellonian University, Doctoral School of Exact and Natural Sciences, prof. S. Łojasiewicza 11, 30-348 Krakow, Poland

[2]Department of Organic Chemistry, Faculty of Chemistry, Jagiellonian University, Gronostajowa 2, 30-387 Krakow, Poland

[3]Recepton Sp. z o.o., ul. Trzy Lipy 3, 80-172 Gdansk, Poland

[*]Corresponding Authors:
Tad A. Holak - tad.holak@receptonbiotech.com
Tomasz Sitar - tomasz.sitar@receptonbiotech.com

## LIST OF CONTENTS

Figure S1. ........ S3

References for Figure S1 ........ S4

Figure S2A.. ........ S5

Figure S2B.. ........ S7

References for Figure S2B ........ S8

Figure S3A. ........ S10

Figure S3B.. ........ S11

Figure S4.. ........ S12

Table S1. ........ S13

Crystallization of the 17a/PD-L1 Complex. ........................................................................................................S14

Crystal Structure Determination and Refinement.........................................................................................S14

References.....................................................................................................................................................S14

General procedure for preparation final compounds ...................................................................................S16

Solubility of the compounds ........................................................................................................................S76

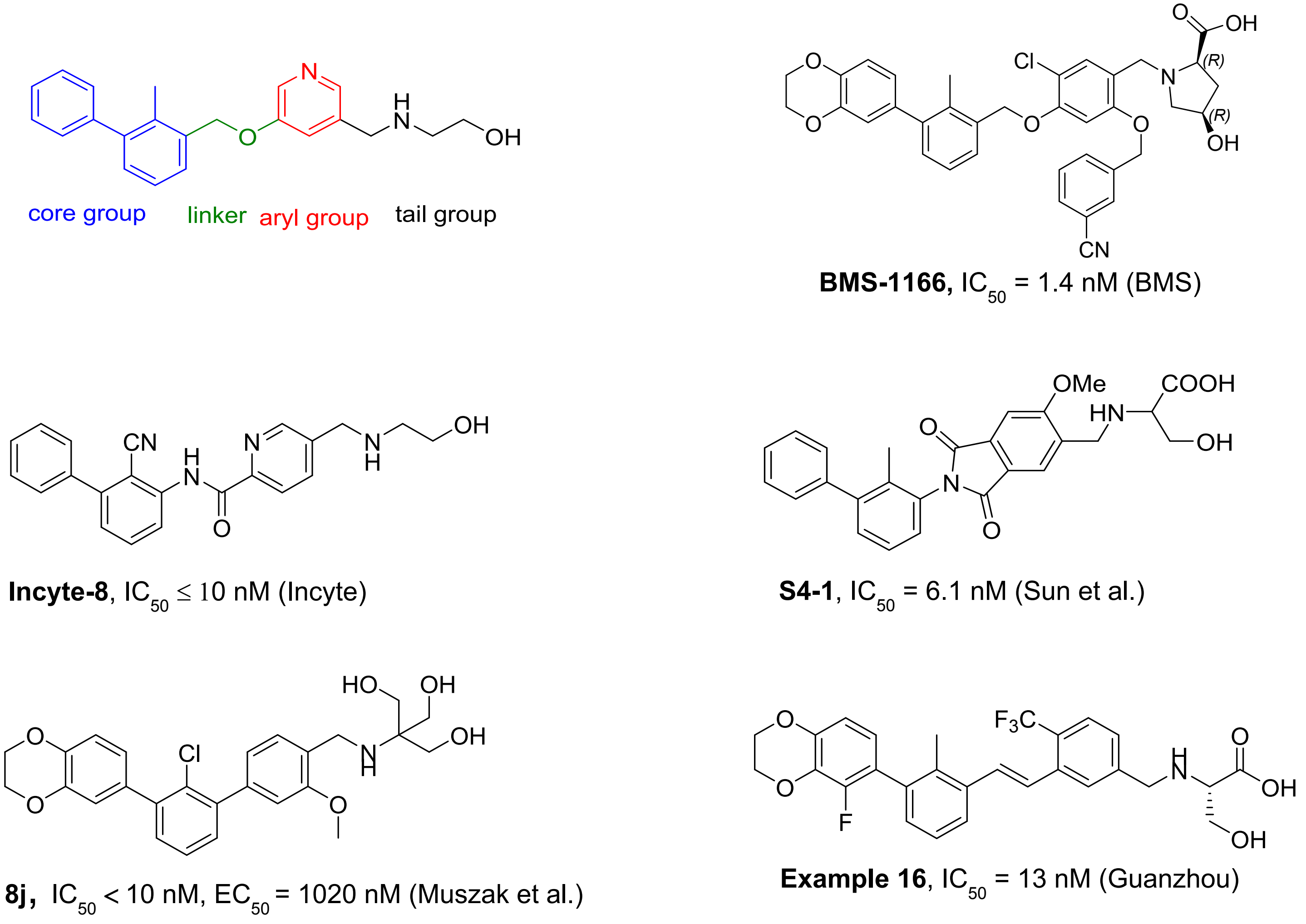


FIGURE S1. **A "short" pharmacophore of small-molecule PD-L1 inhibitors (top left) and representative structures for this pharmacophore.**

## REFERENCES FOR FIGURE S1

BMS, Chupak, L. S.; Ding, M.; Martin, S. W.; Zheng, X.; Hewawasam, P.; Connolly, T. P.; Xu, N.; Yeung, K.-S.; Zhu, J.; Langley, D. R.; Tenney, D. J.; Scola, P. M.; Mingo, P. A. Bristol-Myers Squibb Company, assignee. Compounds Useful as Immunomodulators. U.S. Patent, WO2015160641A2, 2015.

Guanzhou Maxinovel Pharmaceuticals Co Ltd.; Wang, Y.; Zhang, N.; Wu, T.; He, M. Aromatic vinyl or aromatic ethyl derivative, preparation method therefor, intermediate, pharmaceutical composition and application. CN Patent, WO2019128918 A1, 2019.

Incyte Corporation, Wu, L.; Yu, Z.; Zhang, F.; Yao, W. N-phenyl-pyridine-2-carboxamide derivatives and their use as PD-1/PD-L1 protein/protein interaction modulators. U.S. Patent, WO2017106634Al, 2017.

Muszak, D.; Surmiak, E.; Plewka, J.; Magiera-Mularz, K.; Kocik-Krol, J.; Musielak, B.; Sala, D.; Kitel, R.; Stec, M.; Weglarczyk, K.; Siedlar, M.; Domling, A.; Skalniak, L.; Holak, T. A. Terphenyl-Based Small-Molecule Inhibitors of Programmed Cell Death-1/Programmed Death-Ligand 1 Protein−Protein Interaction *J. Med. Chem.* **2021**, *23*, 11614-11636.

Sun, C.L.; Yin, M.X.; Cheng, Y; Kuang, Z.; Liu, X.J.; Wang, G.F.; Wang, X.; Yuan, K.; Min, W.J.; Dong, J.W.; Hou, Y.; Hu, L.R.; Zhang, G.Y.; Pei, W.L.; Wang, L.P.; Sun, Y.Z.; Yu, X.M.; Xiao, Y.B.; Deng, H.B.; Yang, P. Novel Small-Molecule PD-L1 Inhibitor Induces PD L1 Internalization and Optimizes the Immune Microenvironment. *J. Med. Chem*. **2013**, 66, 2064–2083.

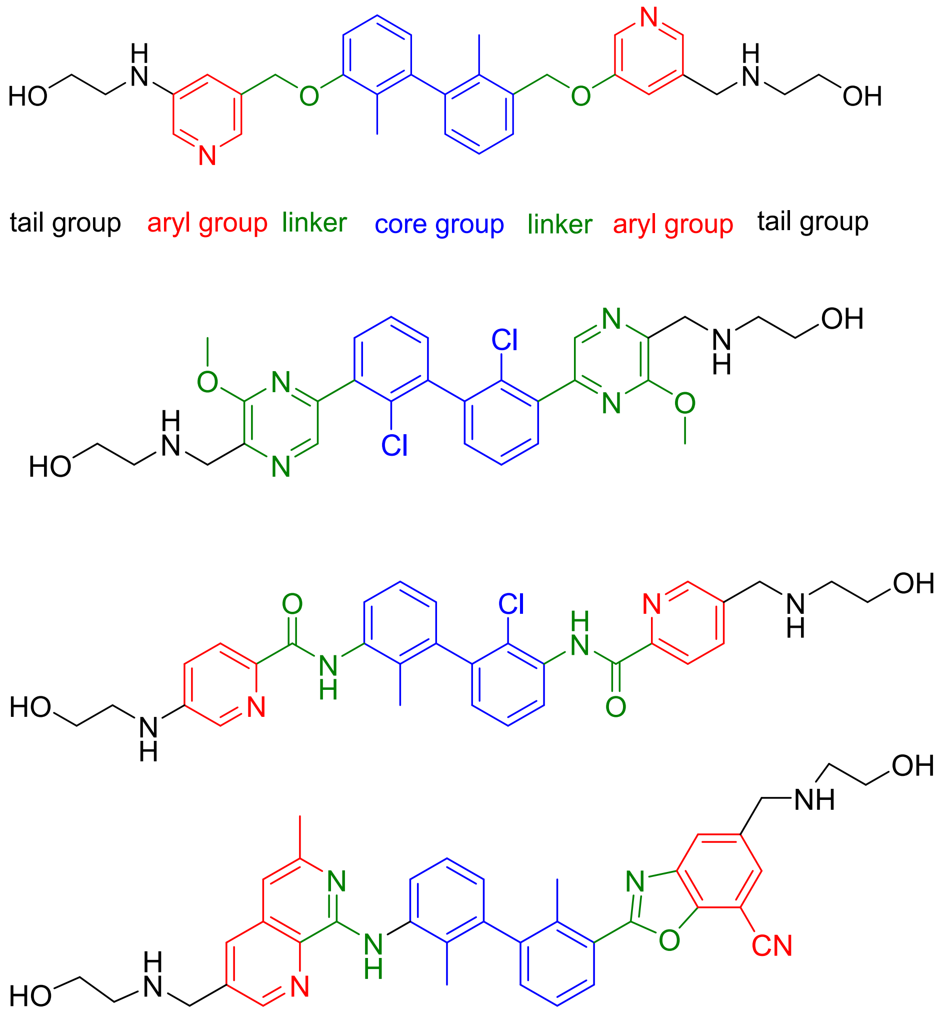


**FIGURE S2A.** THE "LONG" PHARMACOPHORE OF PD-L1 SMALL-MOLECULE INHIBITORS.

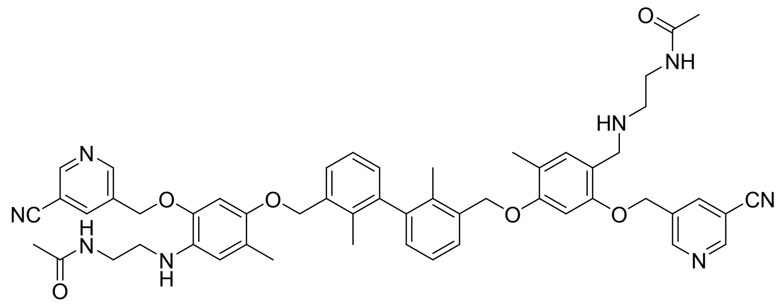


**2b** (**LH1307**), $IC_{50}$ = 3.0 nM, $EC_{50}$ = 763 nM (Basu et al.)

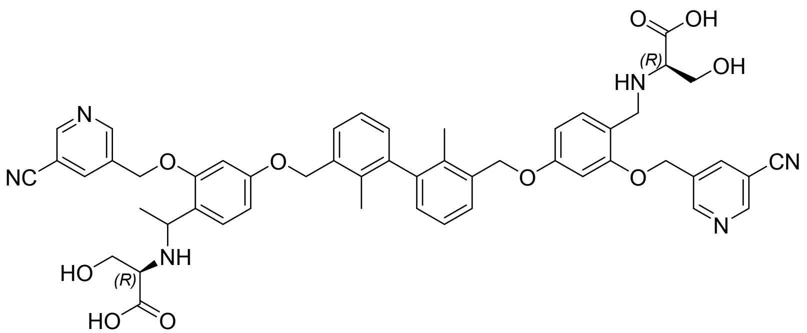


**4**, $K_D$ = 0.019 nM, $EC_{50}$ = 1000 nM (Kawashita et al.)

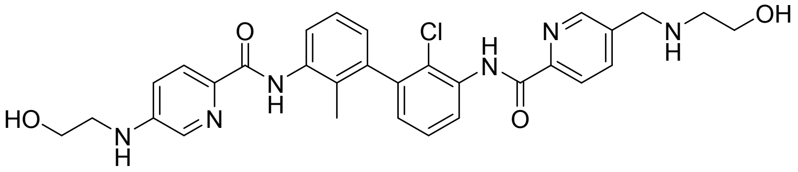


**Incyte-1**, $IC_{50}$ ≤ 10 nM (Incyte, 2018)

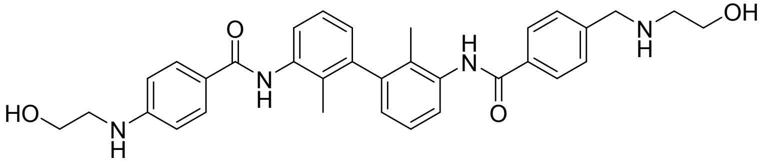


**Cpd A** (**ARB-272572**), $IC_{50}$ = 0.4 nM, $EC_{50}$ 17 nM (Park et al.)

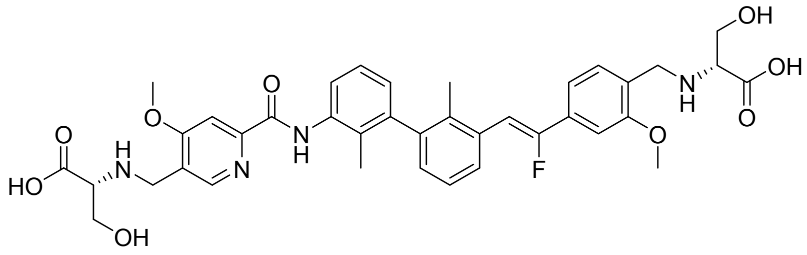


**Example 8**, $IC_{50}$ = 119.0 nM, $EC_{50}$ = 27.63 nM (Abbisko)

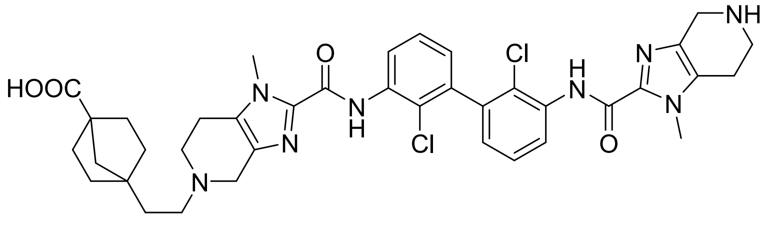


**Example 1**, $IC_{50}$ ≤ 5 nM, $EC_{50}$ > to ≤ 100 nM (Incyte, 2019)

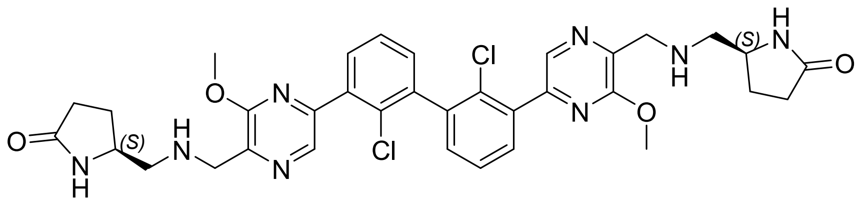

**Example 139**, $IC_{50}$ = 0.213 nM, $EC_{50}$ = 119 nM (Gilead 2019)

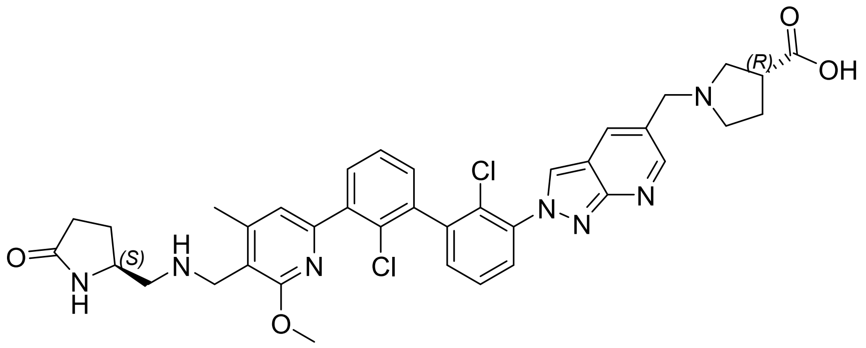

**Example 3**, $IC_{50}$ < 10 nM, $EC_{50}$ < 100 nM

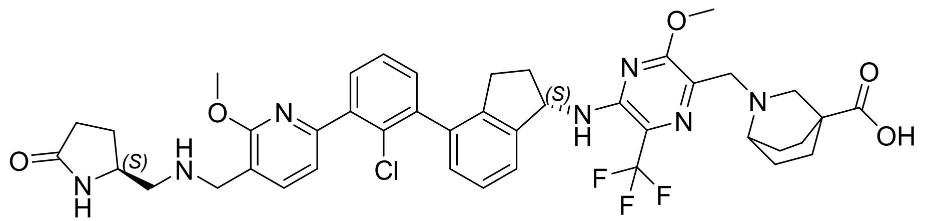

**Example A-18**, $IC_{50}$ < 0.064 nM, $EC_{50}$ 9 nM (Gilead 2021)

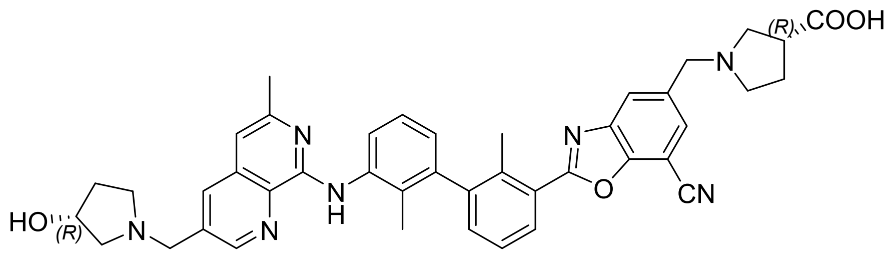

**INCBO86550**, $IC_{50}$ = 3.1 nM, $EC_{50}$ 21.4 nM (Koblish **et al.)**

**Figure S2B.** REPRESENTATIVE STRUCTURES OF THE SECOND GENERATION "C2-SYMMETRIC LONG" COMPOUNDS TARGETING PD-L1.

## REFERENCES FOR FIGURE S2B

Abbisko Therapeutics. Immunosuppressive agent, preparation method therefor, and pharmarceutical use thereof. CN Patent, January 16, 2020, WO2020011209 A1.

Basu, S.; Yang, J.; Xu, B.; Magiera-Mularz, K.; Skalniak, L.; Musielak, B.; Kholodovych, V.; Holak, T. A.; Hu L. Design, Synthesis, Evaluation, and Structural Studies of C2-Symmetric Small Molecule Inhibitors of Programmed Cell Death-1/Programmed Death-Ligand 1 Protein–Protein Interaction. *J. Med. Chem*. **2019**, 62(15):7250–635.

Gilead Sciences Inc. PD-1/PD-L1 inhibitors. U.S. Patent, August 22, 2019, WO2019160882 A1.

Gilead Sciences Inc. PD-1/PD-L1 inhibitors. U.S. Patent, January 28, 2021, US20210024494 A1.

Incyte Corporation; Wu, L.; Yu, Z.; Zhang, F.; Yao, W.; Pyridine derivatives as immunomodulators. U.S. Patent, June 28, 2018, WO 2018119221 A1.

Incyte Corporation; Tetrahydro-imidazo[4,5-c]pyridine derivatives as PD-L1 immunomodulators. U.S. Patent, November 14, 2019, WO2019217821 A1.

Kawashita, S.; Aoyagi, K.; Yamanaka, H.; Hantani, R.; Naruoka, S.; Tanimoto, A.; Horib, Y.; Toyonagab, Y.; Fukushimaa, K.; Miyazakia, S.; Hantani, Y. Symmetry-Based Ligand Design and Evaluation of Small Molecule Inhibitors of Programmed Cell Death-1/Programmed Death-Ligand 1 Interaction. *Bioorg. Med. Chem. Lett.* **2019**, 29:2464–67.

Koblish, H. K.; Wu, L.; Wang, L. S.; Liu, P. C. C.; Wynn, R.; Rios-Doria, J.; Spitz, S.; Liu, H.; Volgina, A.; Zolotarjova, N.; Kapilashrami, K.; Behshad, E.; Covington, M.; Yang, YO.; Li, J.; Diamond, S.; Soloviev, M.; O'Hayer, K.; Rubin, S.; Kanellopoulou, C.; Yang, G.; Rupar, M.; DiMatteo, D.; Lin, L.; Stevens, C.; Zhang, Y.; Thekkat, P.; Geschwindt, R.; Marando, C.; Yeleswaram, S.; Jackson, J.; Scherle,

P.; Huber, R.; Yao, W.; Hollis, G. Characterization of INCB086550: A potent and novel smallmolecule PD-L1 inhibitor. *Cancer Discov*. **2022**, 12(6): 1482-1499.

Park, J. J.; Thi, E. P.; Carpio, V. H.; Bi, Y.; Cole, A. G.; Dorsey, B. D.; Fan, K.; Harasym, T.; Iott, C. L.; Kadhim, S.; Kim, J. H.; Lee, A. C. H.; Nguyen, D.; Paratala, B. S.; Qiu, R.; White, A.; Lakshminarasimhan, D.; Leo, C.; Suto, R. K.; Rijnbrand, R.; Tang, S.; Sofia, M. J.; Moore, C. B. Checkpoint Inhibition through Small Molecule-Induced Internalization of Programmed Death-Ligand 1. *Nat. Commun*. **2021**, 12, 1222.

Shanghai Ennovabio Pharmaceutical Co Ltd. Preparation and application of class of N-containing heterocyclic compounds with immunomodulatory function. CN Patent, WO2021121282 A1, 2021.

A) B)

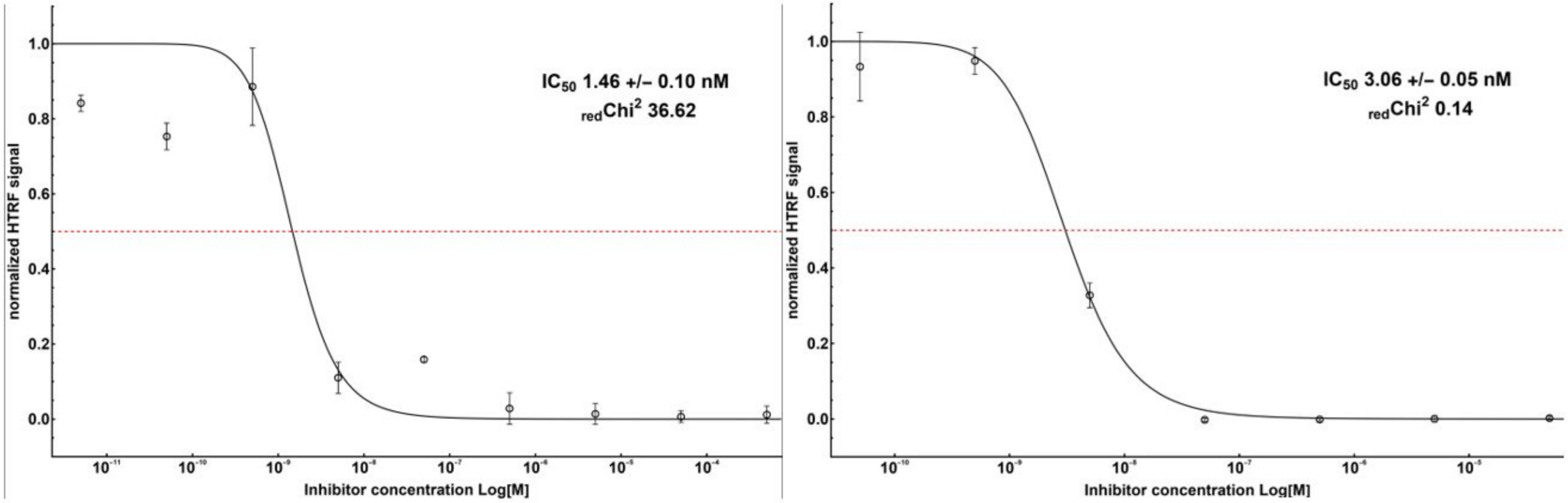


**Figure S3A.** HTRF RESULTS FOR REPRESENTATIVE SMALL MOLECULES. (A) 2, THE ESTIMATED $IC_{50}$ VALUE: 1.46 NM; (B) 17A, THE ESTIMATED $IC_{50}$ VALUE: 3.06 NM.

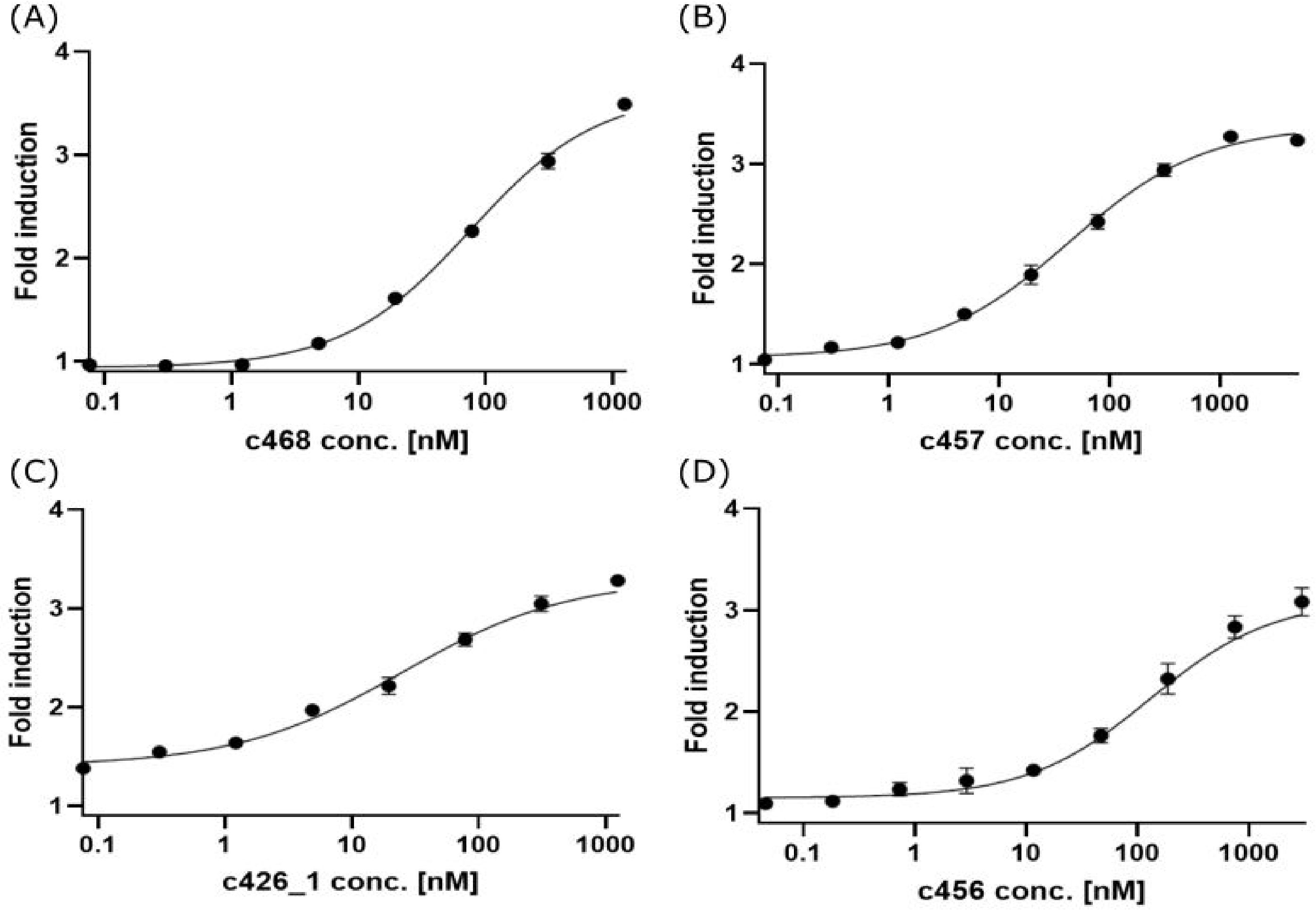


**Figure S3B.** PD-1/PD-L1 BLOCKADE BIOASSAY RESULTS FOR REPRESENTATIVE SMALL MOLECULES. (A) 17A, THE ESTIMATED $EC_{50}$ VALUE: 76,3 NM; (B) 14, THE ESTIMATED $EC_{50}$ VALUE: 34,4 NM; (C) 2A, THE ESTIMATED $EC_{50}$ VALUE: 25 NM; (D) 13, THE ESTIMATED EC50 VALUE: 129,7 NM.

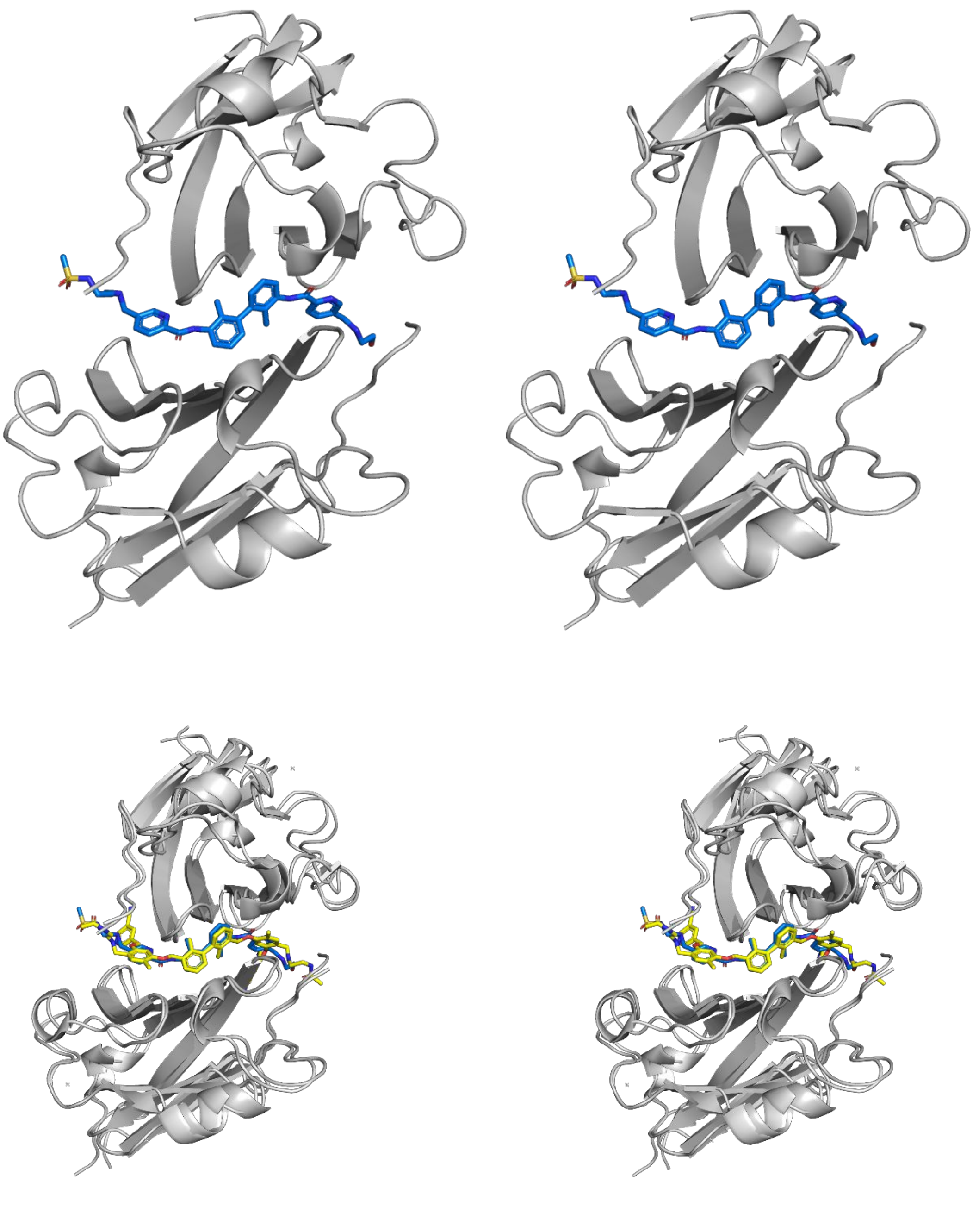

**Figure S4.** STEREO VIEW OF THE 17A CO-STRUCTURE WITH PD-L1 (BLUE, TOP PANEL), AND C2-SYMMEMTRIC ANTI-PD-L1 COMPOUND WITH PD-L1 (PDB ID: 6RPG, YELLOW, BOTTOM PANEL).

**TABLE S1.** DATA COLLECTION AND REFINEMENT STATISTICS (MOLECULAR REPLACEMENT)

| **Data collection** | |
|---|---|
| Wavelength (Å) | |
| Space group | P 1 21 1 |
| Cell dimensions | |
| a, b, c (Å) | 70.75 75.40 75.92 |
| α, β, γ (o) | 90.00 97.25 90.00 |
| Resolution range (Å) | 2.50 - 48.39 |
| Rmerge | 0.148 (2.112) |
| Rmeas | 0.17 (2.48) |
| Rpim | 0.06 (0.91) |
| I/σI | 6.9 (0.9) |
| Completeness (%) | 99.2 (99.8) |
| Redundancy | 7.1 (7.3) |
| Total reflections | 193278 (22790) |
| CC1/2 | 1.00 (0.69) |
| **Refinement statistics** | |
| Unique reflections | 27366 (3124) |
| Rwork/Rfree | 0.25/0.28 |
| Wilson B-factor | 61.35 |
| Ramachandran favoured (%) | 93.6 |
| Ramachandran allowed (%) | 6.0 |
| Ramachandran outliers (%) | 0.4 |

*High resolution shell in parentheses.

## CRYSTALLIZATION OF THE 17A/PD-L1 COMPLEX.

PD-L1 at 5 mg/mL was mixed with 17a in a 1:3 molar ratio (protein–compound). The crystallization screening was carried out using commercially available buffer sets. Initially obtained crystals were optimized. Diffraction-quality crystals were obtained at room temperature from the solution containing 0.1 M Sodium HEPES 7.5 25 % w/v PEG 3350. The crystal was flash cooled in liquid nitrogen without cryoprotection.

## CRYSTAL STRUCTURE DETERMINATION AND REFINEMENT.

The X-ray diffraction data were collected at the P11 beamline at DESY.[1,2] The data were indexed, integrated, and scaled using XDS,[3] And scaled using Aimless.[4]. Data was then process in CCP4 Cloud.[5] The initial phases were obtained by molecular replacement using Phaser,[6] and PDB ID: 5IUS as a search model. The model building was performed using Coot,[7] and refinement was performed in Refmac,[8] and PDB-REDO server.[9] Water molecules were added automatically and inspected manually.

## REFERENCES:

(1) Burkhardt, A.; Pakendorf, T.; Reime, B.; Meyer, J.; Fischer, P.; Stübe, N.; Panneerselvam, S.; Lorbeer, O.; Stachnik, K.; Warmer, M.; Rödig, P.; Göries, D.; Meents, A. Status of the Crystallography Beamlines at PETRA III. *Eur. Phys. J. Plus* **2016**, *131* (3), 56. https://doi.org/10.1140/epjp/i2016-16056-0.

(2) Meents, A.; Reime, B.; Stuebe, N.; Fischer, P.; Warmer, M.; Goeries, D.; Roever, J.; Meyer, J.; Fischer, J.; Burkhardt, A.; Vartiainen, I.; Karvinen, P.; David, C. Development of an In-Vacuum x-Ray Microscope with Cryogenic Sample Cooling for Beamline P11 at PETRA III; Lai, B., Ed.; San Diego, California, United States, 2013; p 88510K. https://doi.org/10.1117/12.2027303.

(3) Kabsch, W. *XDS. Acta Crystallogr D Biol Crystallogr* **2010**, *66* (2), 125–132. https://doi.org/10.1107/S0907444909047337.

(4) Evans, P. R.; Murshudov, G. N. How Good Are My Data and What Is the Resolution? *Acta Crystallogr D Biol Crystallogr* **2013**, *69* (7), 1204–1214. https://doi.org/10.1107/S0907444913000061.

(5) Krissinel, E.; Lebedev, A. A.; Uski, V.; Ballard, C. B.; Keegan, R. M.; Kovalevskiy, O.; Nicholls, R. A.; Pannu, N. S.; Skubák, P.; Berrisford, J.; Fando, M.; Lohkamp, B.; Wojdyr, M.; Simpkin, A. J.; Thomas, J. M. H.; Oliver, C.; Vonrhein, C.; Chojnowski, G.;

Basle, A.; Purkiss, A.; Isupov, M. N.; McNicholas, S.; Lowe, E.; Triviño, J.; Cowtan, K.; Agirre, J.; Rigden, D. J.; Uson, I.; Lamzin, V.; Tews, I.; Bricogne, G.; Leslie, A. G. W.; Brown, D. G. *CCP* 4 Cloud for Structure Determination and Project Management in Macromolecular Crystallography. *Acta Crystallogr D Struct Biol* **2022**, *78* (9), 1079–1089. https://doi.org/10.1107/S2059798322007987.

(6) McCoy, A. J.; Grosse-Kunstleve, R. W.; Adams, P. D.; Winn, M. D.; Storoni, L. C.; Read, R. J. *Phaser* Crystallographic Software. *J Appl Crystallogr* **2007**, *40* (4), 658–674. https://doi.org/10.1107/S0021889807021206.

(7) Emsley, P.; Lohkamp, B.; Scott, W. G.; Cowtan, K. Features and Development of *Coot*. *Acta Crystallogr D Biol Crystallogr* **2010**, *66* (4), 486–501. https://doi.org/10.1107/S0907444910007493.

(8) Murshudov, G. N.; Skubák, P.; Lebedev, A. A.; Pannu, N. S.; Steiner, R. A.; Nicholls, R. A.; Winn, M. D.; Long, F.; Vagin, A. A. *REFMAC* 5 for the Refinement of Macromolecular Crystal Structures. *Acta Crystallogr D Biol Crystallogr* **2011**, *67* (4), 355–367. https://doi.org/10.1107/S0907444911001314.

(9) Joosten, R. P.; Long, F.; Murshudov, G. N.; Perrakis, A. The *PDB_REDO* Server for Macromolecular Structure Model Optimization. *IUCrJ* **2014**, *1* (4), 213–220. https://doi.org/10.1107/S2052252514009324.

## GENERAL PROCEDURE FOR PREPARATION FINAL COMPOUNDS

**Declaration:**

Purity of all compound were above 90%. Purity of final compound were above 95% according to LC-MS and HRMS.

**Compound (2)**

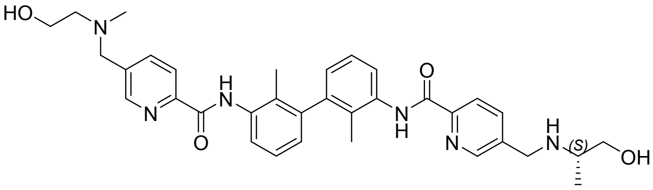


**(S)-5-(((2-hydroxyethyl)(methyl)amino)methyl)-N-(3'-(5-(((1-hydroxypropan-2-yl)amino)methyl)picolinamido)-2,2'-dimethyl-[1,1'-biphenyl]-3-yl)picolinamide**

*2-methyl-3-(4,4,5,5-tetramethyl-1,3,2-dioxaborolan-2-yl)aniline* ***(1.1)***

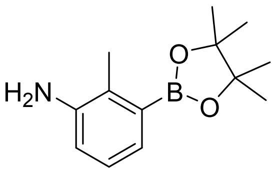


**(1.1)**

A suspension of 3-Bromo-2-methylaniline (2 g, 10.75 mmol, 1 equiv.), bis(pinacolo)diboron (4.095 g, 16.12 mmol, 1.5 equiv.) and potassium acetate (3.165 g, 32.25 mmol, 3 equiv.) in 1,4-dioxane (40 ml) was purged with argon for 15 min. Then, Pd(dppf)$Cl_2$*DCM (0.878 g, 1.07 mmol, 0.1 equiv.) was added and the mixture was stirred at 90°C under argon for 4 days. After cooling to room temperature, the mixture was diluted with EtOAc and extracted. The organic layers were combined, dried over $Na_2SO_4$, filtered, and concentrated under a reduced pressure. Crude was purified by flash chromatography using Hex:EtOAc (0-100%) to give **(1.1)** (3.32 g) as yellowish solid. **$^1$H NMR** (600 MHz, DMSO) δ 6.87 – 6.83 (m, 2H), 6.72 – 6.69 (m, 1H), 4.72 (s, 2H), 2.20 (s, 3H), 1.28 (s, 12H).

*tert-butyl (3-bromo-2-methylphenyl)carbamate* ***(1.2)***

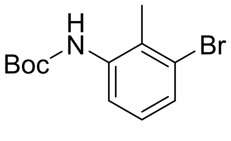


**(1.2)**

3-Bromo-2-methylaniline (8 g, 43 mmol, 1 equiv.) was dissolved in ACN (160 ml). Then DIPEA (22.469 ml, 129 mmol, 3 equiv.) and Di-tert-butyl dicarbonate (12.2 g, 55.9 mmol, 1.3 equiv.) and KI (7.138 g, 43 mmol, 1 equiv.) were added and reaction was left for stirring at 70°C for 2 days. The reaction was subsequently diluted with DCM and the resulting organic solution was washed with saturated aqueous $NaHCO_3$, then brine. The organic layer was dried under sodium sulfate, concentrated, and the crude mixture was purified by silica column chromatography, eluting with

10-90% EtOAc gradient in hexane to afford **(1.2)** (10 g, 81% yield) as white solid. **$^1$H NMR** (300 MHz, DMSO) δ 8.80 (s, 1H), 7.43 – 7.36 (m, 1H), 7.32 – 7.22 (m, 1H), 7.13 – 7.04 (m, 1H), 2.24 (d, *J* = 2.7 Hz, 3H), 1.45 (d, *J* = 2.8 Hz, 9H).

*tert-butyl (3'-amino-2,2'-dimethyl-[1,1'-biphenyl]-3-yl)carbamate* ***(1.3)***

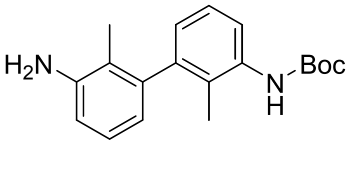


**(1.3)**

Into a flask, **(1.1)** (2.75 g, 11.8 mmol, 1 equiv.), **(1.2)** (3.376 g, 11.80 mmol, 1 equiv.), $K_2CO_3$ (4.891 g, 35.39 mmol, 3 equiv.) were dissolved in water (27.5 ml) and 1,4-dioxane (50 ml) and a solution was degassed by argon bubbling for 15 min. Next, [1,1′Bis(diphenylphosphino)ferrocene]dichloropalladium(II) catalyst (0.863 g, 1.18 mmol, 0.1 equiv.) was added and the reaction was heated at 90°C overnight. After that time, water was added, and reaction was extracted with EtOAc. The organic layer was washed with brine, dried over $Na_2SO_4$ and evaporated. Crude was purified by flash chromatography using Hex:EtOAc (0-100%) to give **(1.3)** (3.48 g, 94% yield) as yellowish oil. **$^1$H NMR** δ 8.54 (s, 1H), 7.29 – 7.23 (m, 1H), 7.14 (t, *J* = 7.7 Hz, 1H), 6.91 (t, *J* = 7.7 Hz, 1H), 6.83 (dd, *J* = 7.5, 1.4 Hz, 1H), 6.63 (dd, *J* = 8.0, 1.3 Hz, 1H), 6.26 (dd, *J* = 7.4, 1.3 Hz, 1H), 4.87 (s, 2H), 1.86 (s, 3H), 1.71 (s, 3H), 1.45 (s, 9H). **$^{13}$C NMR** (101 MHz, $CDCl_3$) δ 165.11, 161.09, 153.38, 153.09, 149.45, 142.11, 139.18, 136.58, 135.77, 128.46, 126.85, 126.52, 126.38, 126.25, 125.30, 122.30, 120.85, 80.67, 77.48, 77.16, 76.84, 68.11, 52.89, 28.48, 28.46, 25.73, 14.51, 14.48, 14.46, 14.33. **LC-MS** (DAD/ESI) (12 min): $t_R$= 6.20 min, Calcd for $C_{19}H_{24}N_2O_2$ (m/z): $[M+H]^+$ 313.19; found, $[M+H]^+$ 313.31, purity: 97%.

*methyl 6-((3'-((tert-butoxycarbonyl)amino)-2,2'-dimethyl-[1,1'-biphenyl]-3-yl)carbamoyl) nicotinate* ***(1.4)***

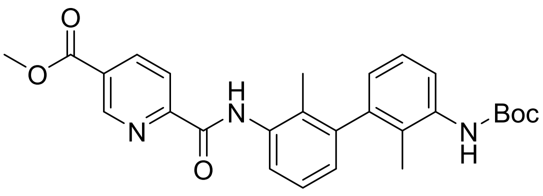


**(1.4)**

To a solution of 5-(Methoxycarbonyl)picolinic acid (2.319 g, 12.80 mmol, 1 equiv.) in DMF (40 ml) was added DIPEA (6.69 ml, 25.61 mmol, 2 equiv.). The mixture was cooled to 0$^o$C and treated with EDC-HCl (4.909 g, 25.61 mmol, 2 equiv.), HOBt (3.922 g, 25.61 mmol, 2 equiv.) and the **(1.3)** (4 g, 12.8 mmol, 1 equiv.). The reaction was stirred at RT overnight. Then water was added, and the reaction was extracted with DCM. The organic layer was washed with brine, dried over $Na_2SO_4$ and evaporated. Crude was purified by flash chromatography using DCM:MeOH (0-8%) to give **(1.4)** (5.33 g, 88% yield)

as yellowish solid. **$^{1}$H NMR** (400 MHz, $CDCl_3$) δ 10.15 (s, 1H), 9.22 (dd, *J* = 2.0, 0.8 Hz, 1H), 8.52 (dd, *J* = 8.1, 2.0 Hz, 1H), 8.41 (dd, *J* = 8.1, 0.9 Hz, 1H), 8.25 (dd, *J* = 8.1, 1.3 Hz, 1H), 7.83 (d, *J* = 8.0 Hz, 1H), 7.31 (t, *J* = 7.9 Hz, 1H), 7.23 (d, *J* = 7.9 Hz, 1H), 6.97 (dd, *J* = 7.6, 1.3 Hz, 1H), 6.90 (dd, *J* = 7.6, 1.3 Hz, 1H), 6.37 (s, 1H), 4.00 (s, 3H), 2.10 (s, 3H), 1.96 (s, 3H), 1.53 (s, 9H). **$^{13}$C NMR** (101 MHz, $CDCl_3$) δ 165.11, 161.09, 153.38, 153.09, 149.45, 142.11, 139.18, 136.58, 135.77, 128.46, 126.85, 126.52, 126.38, 126.25, 125.30, 122.30, 120.85, 80.67, 77.48, 77.16, 76.84, 68.11, 52.89, 28.48, 28.46, 25.73, 14.51, 14.48, 14.46, 14.33.

*tert-butyl (3'-(5-(hydroxymethyl)picolinamido)-2,2'-dimethyl-[1,1'-biphenyl]-3-yl)carbamate **(1.5)***

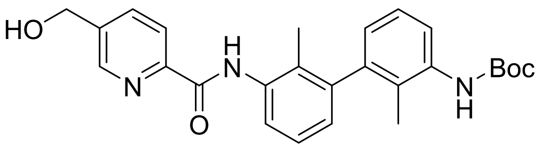


**(1.5)**

**(1.4)** (5.33 g, 11.21 mmol. 1 equiv.) was dissolved in THF/MeOH (2:1) (79.75 ml) and cooled to 0°C. Then a solution of 4M $LiBH_4$ in THF (8.406 ml, 33.62 mmol, 3 equiv.) was added dropwise and reaction was stirred at this temperature for 15 min and then left to warm to RT. Reaction was monitored by TLC. After full consumption of SM (15 min) water was added and solvent was evaporated. To the residue DCM was added and reaction was extracted with DCM/water. The organic layer was washed with brine, dried over $Na_2SO_4$ and evaporated to give **(1.5)** (4.46 g, 89% yield) as yellowish solid. **$^{1}$H NMR** (400 MHz, DMSO-$D_6$) δ 10.33 (s, 1H), 8.66 (dd, *J* = 2.1, 0.9 Hz, 1H), 8.61 (s, 1H), 8.15 (dd, *J* = 7.9, 0.8 Hz, 1H), 7.99 (ddt, *J* = 8.0, 1.8, 0.8 Hz, 1H), 7.85 (dd, *J* = 8.1, 1.3 Hz, 1H), 7.37 – 7.26 (m, 2H), 7.21 (t, *J* = 7.8 Hz, 1H), 6.92 (ddd, *J* = 13.8, 7.6, 1.4 Hz, 2H), 5.54 (t, *J* = 5.6 Hz, 1H), 4.66 (d, *J* = 4.7 Hz, 2H), 1.98 (d, *J* = 2.7 Hz, 6H), 1.46 (s, 9H). **$^{13}$C NMR** (101 MHz, DMSO-$D_6$) δ 162.15, 153.70, 148.36, 146.88, 142.07, 141.77, 141.42, 136.95, 136.22, 135.97, 130.17, 128.62, 126.03, 125.76, 125.71, 125.47, 124.15, 124.13, 121.88, 78.71, 60.38, 40.15, 39.99, 39.94, 39.78, 39.73, 39.52, 39.31, 39.10, 38.89, 30.43, 28.17, 20.78, 14.86, 14.10.

*tert-butyl (3'-(5-(bromomethyl)picolinamido)-2,2'-dimethyl-[1,1'-biphenyl]-3-yl)carbamate **(1.6)***

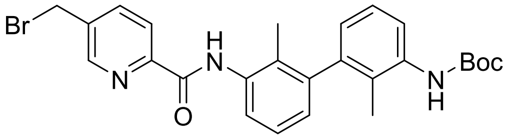

**(1.6)**

**(1.5)** (2.5 g, 5.59 mmol, 1 equiv.) was dissolved in DCM (25 ml), then NBS (1.491 g, 8.38 mmol, 1.5 equiv.) and $PPh_3$ (2,198 g, 8.38 mmol, 1.5 equiv.) were added. Reaction was left for stirring for 30 min (no SM). Then water was added, and reaction was extracted with DCM. The organic layer was washed with brine, dried over $Na_2SO_4$ and evaporated. Crude was purified by flash chromatography using Hex:EtOAc (0-50%) to give **(1.6)** (1.8 g, 63% yield) as white foam. **$^1$H NMR** (300 MHz, DMSO) δ 10.32 (s, 1H), 8.82 – 8.75 (m, 1H), 8.58 (s, 1H), 8.18 – 8.08 (m, 1H), 7.80 (d, *J* = 8.2 Hz, 1H), 7.33 (t, *J* = 7.3 Hz, 2H), 7.21 (t, *J* = 7.7 Hz, 1H), 6.96 – 6.85 (m, 1H), 4.88 – 4.80 (m, 2H), 2.01 – 1.94 (m, 3H), 1.89 (d, *J* = 4.6 Hz, 3H), 1.50 – 1.40 (m, 9H).

*tert-butyl (3'-(5-(((2-hydroxyethyl)(methyl)amino)methyl)picolinamido)-2,2'-dimethyl-[1,1'-biphenyl]-3-yl) carbamate **(1.7_2)***

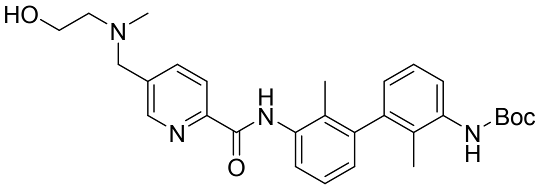

**(1.7_2)**

**(1.6)** (0.8 g, 1.57 mmol, 1 equiv.) was dissolved in ACN (16 ml), then $K_2CO_3$ (0.65 g, 4.7 mmol, 3 equiv.), KI (0.26 g, 1.57 mmol, 1 equiv.) and 2-(Methylamino)ethanol (0.252 ml, 3.13 mmol, 2 equiv.) was added. The mixture was left for stirring at 50°C overnight. Next, water was added, and the reaction was extracted with DCM. The organic layer was washed with brine, dried over $Na_2SO_4$ and evaporated to give **(1.7_2)** (0.87 g of crude) as yellowish oil. **$^1$H NMR** (300 MHz, DMSO) δ 10.32 (s, 1H), 8.68 – 8.63 (m, 1H), 8.58 (s, 1H), 8.14 (d, *J* = 8.0 Hz, 1H), 8.03 – 7.98 (m, 1H), 7.85 (d, *J* = 8.0 Hz, 1H), 7.31 (q, *J* = 7.6 Hz, 2H), 7.21 (t, *J* = 7.7 Hz, 1H), 6.92 (dd, *J* = 10.0, 7.7 Hz, 2H), 4.42 (d, *J* = 5.4 Hz, 1H), 3.66 (s, 2H), 3.53 (q, *J* = 6.0 Hz, 2H), 2.46 (d, *J* = 6.3 Hz, 2H), 1.98 (d, *J* = 2.8 Hz, 3H), 1.89 (s, 3H), 1.46 (s, 9H).

*N-(3'-amino-2,2'-dimethyl-[1,1'-biphenyl]-3-yl)-5-(((2-hydroxyethyl)(methyl) amino)methyl)picolinamide **(1.8_2)***

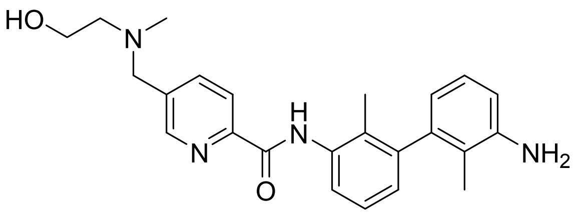

**(1.8_2)**

**(1.7_2)** (0.87 g, 1.72 mmol, 1 equiv.) was dissolved in DCM (26.1 ml) then 6 M HCl in i-PrOH (2.873 ml, 17.24 mmol, 10 equiv.) was added and reaction was left for stirring at RT overnight. Then, 2 M NaOH was added to obtain pH~8 and reaction was extracted with DCM and water. The organic layer was washed with brine, dried over $Na_2SO_4$ and evaporated

to give **(1.8_2)** (0.49 g, 70% yield) as colorless oil. **$^1$H NMR** (300 MHz, DMSO) δ 10.28 (d, *J* = 3.3 Hz, 1H), 8.65 (t, *J* = 2.6 Hz, 1H), 8.14 (dd, *J* = 7.9, 0.9 Hz, 1H), 8.00 (dd, *J* = 7.9, 2.2 Hz, 1H), 7.86 – 7.80 (m, 1H), 7.31 – 7.22 (m, 1H), 6.98 – 6.89 (m, 2H), 6.65 (dt, *J* = 8.0, 2.3 Hz, 1H), 6.31 (dt, *J* = 7.4, 2.0 Hz, 1H), 4.89 (s, 1H), 4.49 – 4.40 (m, 2H), 3.65 (d, *J* = 3.4 Hz, 2H), 3.53 (q, *J* = 6.0 Hz, 2H), 3.29 (d, *J* = 1.8 Hz, 2H), 2.19 (d, *J* = 3.5 Hz, 3H), 1.98 (dd, *J* = 3.7, 2.0 Hz, 3H), 1.74 (d, *J* = 3.3 Hz, 3H).

*N-(3'-amino-2,2'-dimethyl-[1,1'-biphenyl]-3-yl)-5-(((2-((tert-butyldimethylsilyl) oxy)ethyl)(methyl)amino)methyl)picolinamide* ***(1.9_2)***

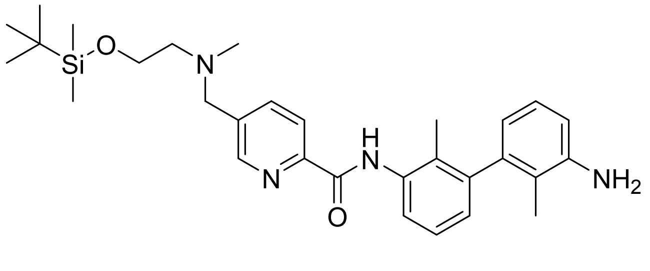

**(1.9_2)**

**(1.8_2)** (0.49 g, 1.21 mmol, 1 equiv.) was dissolved in DMF (7.35 ml) and cooled on an ice bath. Imidazole (0.206 g, 3.03 mmol, 2.5 equiv.) was then added slowly (over ˜10 minutes). A solution of TBDMS-Cl (0.228 g, 1.51 mmol, 1.25 equiv.) in DMF (1.14 ml, 5 equiv.) was added (over ˜2 minutes). The ice bath was removed and the solution was stirred at room temperature for 1 h (no SM). Then water was added and reaction was extracted with EtOAc. The organic layer was washed with brine, dried over $Na_2SO_4$ and evaporated to give **(1.9_2)** (0.44 g of crude, 70% yield) as yellowish oil. **$^1$H NMR** (300 MHz, DMSO) δ 10.28 (d, *J* = 2.8 Hz, 1H), 8.64 (d, *J* = 2.1 Hz, 1H), 8.13 (dd, *J* = 8.1, 2.5 Hz, 1H), 7.98 (dd, *J* = 8.0, 2.3 Hz, 1H), 7.84 (d, *J* = 8.0 Hz, 1H), 7.30 – 7.20 (m, 1H), 6.97 – 6.88 (m, 2H), 6.72 – 6.62 (m, 1H), 6.30 (dd, *J* = 7.6, 2.8 Hz, 1H), 4.89 (s, 2H), 3.76 – 3.67 (m, 4H), 2.22 (d, *J* = 2.6 Hz, 3H), 1.98 (dt, *J* = 3.6, 1.3 Hz, 3H), 1.74 (d, *J* = 2.5 Hz, 3H), 0.88 – 0.83 (m, 9H), 0.08 – 0.00 (m, 6H).

*methyl 6-((3'-(5-(((2-((tert-butyldimethylsilyl)oxy)ethyl)(methyl)amino) methyl)picolinamido)-2,2'-dimethyl-[1,1'-biphenyl]-3-yl)carbamoyl)nicotinate* ***(1.10_2)***

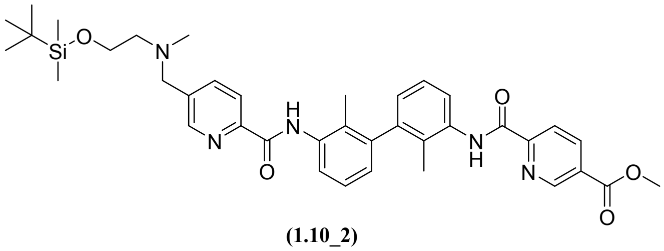

**(1.10_2)**

To a solution of 5-(Methoxy-carbonyl)picolinic acid (0.154 g, 0.85 mmol, 1 equiv.) in DMF (13.20 ml) was added TEA (0.236 ml, 1.7 mmol, 2 equiv.). The mixture was cooled to 0$^o$C and treated with HATU (0.645 g, 1.7 mmol, 2 equiv.) and the amine **(1.9_2)** (0.44 g, 0.85 mmol, 1 equiv.). The reaction was stirred at RT overnight. Then water was added, and reaction was extracted with DCM. The organic layer was washed with brine, dried over $Na_2SO_4$ and

evaporated. Crude was purified by flash chromatography using DCM:MeOH (8%) to give **(1.10_2)** (0.65 g) as yellowish oil.

*5-(((2-((tert-butyldimethylsilyl)oxy)ethyl)(methyl)amino)methyl)-N-(3'-(5-(hydroxymethyl) picolinamido)-2,2'-dimethyl-[1,1'-biphenyl]-3-yl)picolinamide* ***(1.11_2)***

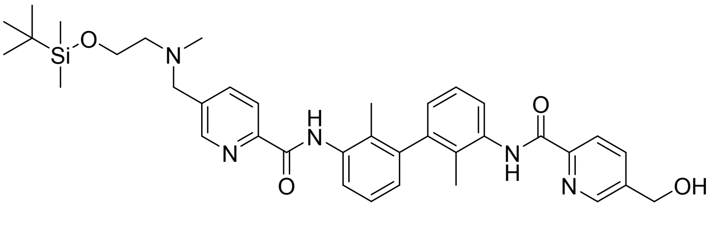

**(1.11_2)**

**(1.10_2)** (0.58 g, 0.85 mmol, 1 equiv.) was dissolved in THF/MeOH (2:1, 8.70 ml) and cooled to 0°C. Then a solution of 4M $LiBH_4$ in THF (1.063 ml, 5 equiv.) was added dropwise, and reaction was stirred at this temperature for 15 min and then left to warm to RT. Reaction was monitored by TLC. After full consumption of SM (15 min) water was added and solvent was evaporated. To the residue DCM was added and reaction was extracted with DCM/water. The organic layer was washed with brine, dried over $Na_2SO_4$ and evaporated to give **(1.11_2)** (0.23 g, 41% yield) as yellowish oil. **$^1$H NMR** (300 MHz, DMSO) δ 10.32 (d, *J* = 3.1 Hz, 2H), 8.66 (q, *J* = 2.2 Hz, 2H), 8.14 (dt, *J* = 8.4, 4.0 Hz, 2H), 8.04 – 7.96 (m, 2H), 7.88 (d, *J* = 8.5 Hz, 2H), 7.32 (td, *J* = 7.9, 3.1 Hz, 2H), 6.99 (dd, *J* = 7.6, 3.1 Hz, 2H), 5.50 (t, *J* = 4.6 Hz, 1H), 4.66 (d, *J* = 5.2 Hz, 2H), 4.02 (qd, *J* = 7.2, 3.1 Hz, 2H), 3.69 (dd, *J* = 6.1, 3.1 Hz, 4H), 2.22 (d, *J* = 3.1 Hz, 3H), 2.02 (d, *J* = 3.0 Hz, 6H), 0.84 (d, *J* = 3.1 Hz, 9H), 0.02 (d, *J* = 3.2 Hz, 6H). **LC-MS** (DAD/ESI): $t_R$= 6.63 min, Calcd for $C_{37}H_{47}N_5O_4Si$ (m/z): $[M+H]^+$ 654.35; found, $[M+H]^+$ 654.45, purity: 90%.

*5-(((2-((tert-butyldimethylsilyl)oxy)ethyl)(methyl)amino)methyl)-N-(3'-(5-formylpicolinamido)-2,2'-dimethyl-[1,1'-biphenyl]-3-yl)picolinamide* ***(1.12_2)***

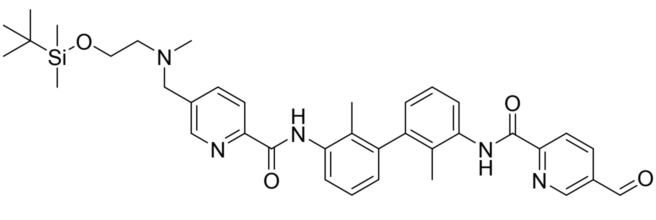

**(1.12_2)**

**(1.11_2)** (0.23 g, 0.35 mmol, 1 equiv.) was dissolved in DCM and cooled to 0°C. Then, Dess-Martin periodinane (0.298 g, 0.7 mmol, 2 equiv.) and $NaHCO_3$ (0.148 g, 1.76 mmol, 5 equiv.) were added and reaction was stirred for 0.5 h at 0°C. Next reaction was left warm to RT and stirred for 30 min (no SM). After that time water was added and reaction was extracted with DCM. The organic layer was washed with brine, dried over $Na_2SO_4$ and evaporated. Crude was purified by flash chromatography using Hex:EtOAC (0-100%) to give **(1.12_2)** (0.17 g, 74% yield) as yellowish solid. **$^1$H NMR** (300 MHz, DMSO) δ 10.49 (s, 1H), 10.33 (s, 1H), 10.23 (d, *J* = 2.9 Hz, 1H), 9.23 – 9.16 (m, 1H), 8.65 (s, 1H), 8.55 – 8.47 (m, 1H), 8.39 – 8.33 (m, 1H), 8.16 – 8.10 (m, 1H), 8.02 – 7.97 (m, 1H), 7.88 (d, *J* = 7.8 Hz, 1H), 7.79 (d, *J* = 8.0 Hz, 1H), 7.40 – 7.27 (m,

2H), 7.06 – 6.97 (m, 2H), 3.73 – 3.65 (m, 4H), 3.30 (s, 8H), 2.23 (d, *J* = 2.8 Hz, 3H), 2.02 (d, *J* = 2.8 Hz, 6H), 0.85 (d, *J* = 2.9 Hz, 9H), 0.02 (d, *J* = 3.0 Hz, 6H).

*(S)-5-(((2-((tert-butyldimethylsilyl)oxy)ethyl)(methyl)amino)methyl)-N-(3'-(5-(((1-hydroxypropan-2-yl)amino)methyl)picolinamido)-2,2'-dimethyl-[1,1'-biphenyl]-3yl)picolinamide* ***(1.13_2)***

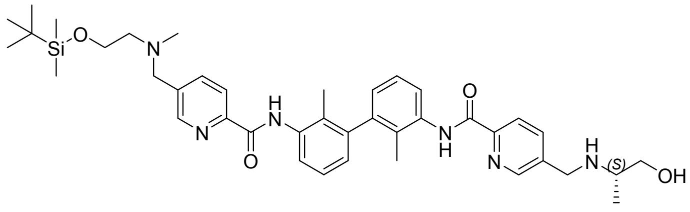

**(1.13_2)**

**(1.12_2)** (0.17 g, 0.26 mmol, 1 equiv.), 2-Amino-1-propanol (0.08 ml, 1.04 mmol, 4 equiv.), and few drops of AcOH were dissolved in a mixture of DCE/MeOH/DMF (2:1:1, 6 ml) and left for stirring for 2 h. Then $NaBH_3CN$ (0.164 g, 2.61 mmol, 10 equiv.) was added and reaction was left for overnight stirring. After that, water was added, and the reaction was extracted with EtOAc. The organic layer was washed with brine, dried over $Na_2SO_4$ and evaporated. Crude was purified by flash chromatography using DCM:MeOH (0-20%) to give **(1.13_2)** (0.1 g, 54% yield) as yellowish oil. **$^1$H NMR** (600 MHz, DMSO) δ 10.34 (d, *J* = 3.6 Hz, 2H), 8.71 – 8.60 (m, 2H), 8.13 (ddd, *J* = 8.0, 4.4, 0.8 Hz, 2H), 8.03 (dd, *J* = 8.0, 2.1 Hz, 1H), 7.99 (dd, *J* = 8.0, 2.1 Hz, 1H), 7.92 – 7.86 (m, 1H), 7.33 (t, *J* = 7.8 Hz, 2H), 6.99 (dt, *J* = 7.6, 1.3 Hz, 2H), 4.59 – 4.52 (m, 1H), 3.90 (d, *J* = 14.6 Hz, 1H), 3.84 (d, *J* = 14.6 Hz, 1H), 3.70 (dd, *J* = 12.4, 6.3 Hz, 3H), 3.30 – 3.28 (m, 2H), 2.62 – 2.58 (m, 1H), 2.23 (s, 3H), 2.02 (d, *J* = 1.8 Hz, 6H), 0.95 (d, *J* = 6.3 Hz, 2H), 0.85 (s, 9H), 0.03 (s, 6H).

*(S)-5-(((2-hydroxyethyl)(methyl) amino)methyl)-N-(3'-(5-(((1-hydroxypropan-2-yl)amino)-methyl)picolinamido)-2,2'-dimethyl-[1,1'-biphenyl]-3-yl)picolinamide* ***(2)***

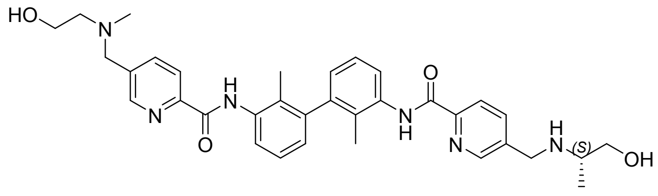

**(2)**

**(1.13_2)** (0.1 g, 0.14 mmol, 1 equiv.) was dissolved in DCM (3 ml) then 6 M HCl in i-PrOH (0.117 ml, 0.7 mmol, 5 equiv.) was added and reaction was left for stirring at RT overnight. Next, solvent was evaporated, and residue was dissolved in water. Then 2 M NaOH was added to obtain pH~8 and reaction was extracted with DCM. The organic layer was washed with brine, dried over $Na_2SO_4$ and evaporated. Crude was purified by pTLC using DCM:MeOH (30%) to give (**2)** (0.005 g, 6% yield) as yellowish oil. **$^1$H NMR** (600 MHz, MeOD) δ 8.68 – 8.63 (m, 2H), 8.17 (td, J = 8.0, 0.8 Hz, 2H), 8.01 (ddd, J = 15.5, 8.0, 2.1 Hz, 2H), 7.93 (dd, J = 8.1, 1.3 Hz, 2H), 7.29 (t, J = 7.8 Hz, 2H), 7.01 (dt, J = 7.6, 1.1 Hz, 2H), 4.00 (d, J = 13.8 Hz, 1H), 3.91 (d, J = 13.9 Hz, 1H), 3.71 – 3.67 (m, 4H), 3.56 (dd, J = 11.0, 4.7 Hz, 1H), 3.45 (dd, J = 11.0, 6.9 Hz,

1H), 2.87 – 2.80 (m, 1H), 2.59 (t, J = 5.9 Hz, 2H), 2.27 (s, 3H), 2.07 (s, 6H), 1.11 (d, J = 6.5 Hz, 3H). **$^{13}$C NMR** (151 MHz, MeOD) δ 164.41, 164.35, 150.54, 149.98, 143.69, 140.01, 139.73, 139.39, 139.00, 137.18, 137.16, 129.93, 127.84, 127.10, 123.54, 123.11, 122.98, 66.32, 60.40, 60.30, 60.13, 55.30, 49.42, 49.28, 49.14, 49.00, 48.86, 48.72, 48.58, 42.72, 16.50, 14.90. **LC-MS** (DAD/ESI) (12 min): $t_R$= 3.50 min, Calcd for $C_{34}H_{40}N_6O_4$ (m/z): $[M+H]^+$ 597.32; found, $[M+H]^+$ 597.42, purity: 99%. **HRMS** (ESI): Calcd for $C_{34}H_{40}N_6O_4$ (m/z): $[M+H]^+$ 597.3189; found, $[M+H]^+$ 597.3217, purity: 95%.

**Compound (1)**

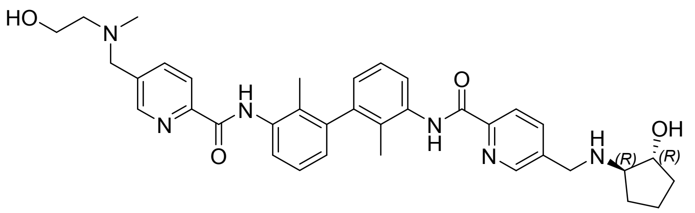

**5-((((1R,2R)-2-hydroxycyclopentyl)amino)methyl)-N-(3'-(5-(((2-hydroxyethyl)(methyl)amino)methyl)picolinamido)-2,2'-dimethyl-[1,1'-biphenyl]-3-yl)picolinamide**

*Synthesis of 5-(((2-((tert-butyldimethylsilyl)oxy)ethyl) (methyl)amino)methyl)-N-(3'-(5-((((1R,2R)-2-hydroxycyclopentyl)amino)methyl) picolinamido)-2,2'-dimethyl-[1,1'-biphenyl]-3-yl)picolinamide* ***(1.13_1)***

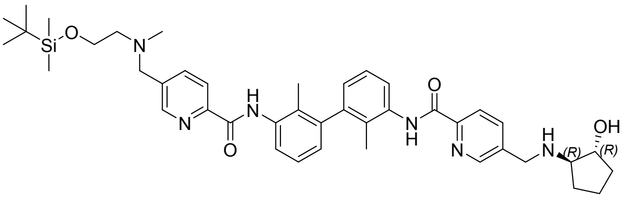

(1.13_1)

**(1.12_1)** (0.2 g, 0.3 mmol, 1 equiv.), (1R,2R)-trans-2-Aminocyclopentanol hydrochloride (0.084 g, 0.61 mmol, 2 equiv.), and few drops of AcOH were dissolved in a mixture of DCE/MeOH (20:10, 6 ml) and left for stirring for 2 h. Then $NaBH_3CN$ (0.193 g, 3.07 mmol, 10 equiv.) was added and reaction was left for overnight stirring. After that, water was added and reaction was extracted with EtOAc. The organic layer was washed with brine, dried over $Na_2SO_4$ and evaporated. Crude was purified by flash chromatography using DCM:MeOH (0-20%) to give **(1.13_1)** (0.087 g, 38% yield) as yellowish oil. **$^1$H NMR** (600 MHz, DMSO) δ 10.32 (s, 2H), 8.67 (s, 1H), 8.62 (d, *J* = 2.0 Hz, 1H), 8.11 (d, *J* = 8.0 Hz, 2H), 8.05 – 7.95 (m, 2H), 7.85 (d, *J* = 7.6 Hz, 2H), 7.30 (t, *J* = 7.8 Hz, 2H), 7.00 – 6.95 (m, 2H), 4.74 (d, *J* = 4.2 Hz, 1H), 3.67 (dd, *J* = 12.2, 6.2 Hz, 4H), 2.20 (s, 3H), 1.99 (d, *J* = 1.6 Hz, 6H),

1.77 (ddt, $J$ = 21.3, 13.2, 7.7 Hz, 2H), 1.60 – 1.52 (m, 3H), 1.47 – 1.37 (m, 2H), 0.82 (s, 9H), -0.00 (s, 6H).

*Synthesis of 5-((((1R,2R)-2-hydroxycyclopentyl) amino)methyl)-N-(3'-(5-(((2-hydroxyethyl)(methyl)amino)methyl)picolinamido)-2,2'-dimethyl-[1,1'-biphenyl]-3-yl)picolinamide* ***(1)***

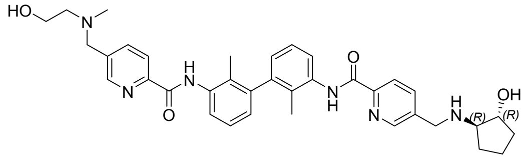

(1)

**(1.13_1)** (0.087 g, 0.12 mmol, 1 equiv.) was dissolved in DCM (2.61 ml) then 6 M HCl in i-PrOH (0.197 ml, 10 equiv.) was added and reaction was left for stirring at RT overnight. Next, solvent was evaporated and residue was dissolved in water. Then 2 M NaOH was added and reaction was extracted with DCM. The organic layer was washed with brine, dried over $Na_2SO_4$ and evaporated. Crude was purified by pTLC using DCM:MeOH (8:2) to give **(1)** (0.03 g, 15% yield) as yellowish solid. **$^1$H NMR** (600 MHz, MeOD) δ 8.69 (d, $J$ = 8.8 Hz, 2H), 8.20 (dd, $J$ = 8.0, 2.6 Hz, 2H), 8.05 – 8.01 (m, 2H), 7.92 (d, $J$ = 8.1 Hz, 2H), 7.34 (t, $J$ = 7.8 Hz, 2H), 7.06 (d, $J$ = 7.5 Hz, 2H), 3.96 (s, 3H), 3.73 (s, 2H), 3.70 (t, $J$ = 5.9 Hz, 2H), 2.92 – 2.87 (m, 1H), 2.61 (d, $J$ = 5.9 Hz, 2H), 2.30 (s, 3H), 2.11 (s, 7H), 2.06 – 2.02 (m, 1H), 1.99 – 1.93 (m, 1H), 1.73 – 1.69 (m, 2H), 1.57 (dt, $J$ = 14.4, 7.0 Hz, 1H), 1.45 – 1.39 (m, 1H), 1.29 (s, 1H). **$^{13}$C NMR** (101 MHz, METHANOL-D4) δ 164.51, 164.40, 150.64, 150.27, 150.19, 150.07, 143.74, 139.83, 139.34, 139.20, 139.05, 137.20, 130.13, 127.95, 127.13, 123.72, 123.14, 123.01, 78.40, 66.83, 60.31, 60.25, 60.10, 49.64, 49.43, 49.21, 49.00, 48.79, 48.57, 48.36, 42.68, 34.16, 30.68, 21.82, 14.91. **LC-MS** (DAD/ESI): $t_R$= 3.66 min, Calcd for $C_{36}H_{42}N_6O_4$ (m/z): $[M+H]^+$ 623.33; found, $[M+H]^+$ 623.47, purity: 100%.

**Compound (2a)**

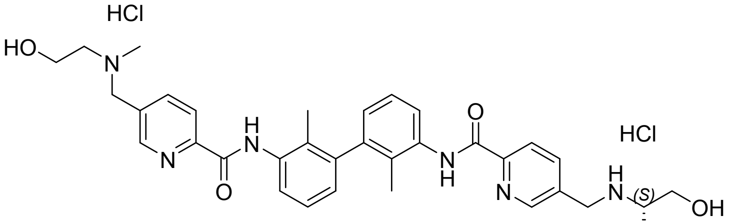

**(S)-5-(((2-hydroxyethyl)(methyl)amino)methyl)-N-(3'-(5-(((1-hydroxypropan-2-yl)amino)methyl)picolinamido)-2,2'-dimethyl-[1,1'-biphenyl]-3-yl)picolinamide trihydrochloride**

**(2)** (0.003 g, 0.005 mmol, 1 equiv.) was dissolved in MeOH/DCM (10:10, 0.6 ml) and 6 M HCl in i-PrOH (0.008 ml, 0.05 mmol, 10 equiv.) was added and reaction was left for stirring for 4 h. Then solvent was evaporated to give **(2a)** as HCl salt. **$^1$H NMR** (600 MHz, MeOD) δ 9.05 (d, J = 15.8 Hz, 2H), 8.62 – 8.44 (m, 4H), 7.77 (dd, J = 27.6, 7.9 Hz, 2H), 7.34 (t, J = 7.7 Hz, 2H), 7.08 (t, J = 7.2 Hz, 2H), 4.80 (d, J = 12.8 Hz, 1H), 4.62 (d, J = 13.2 Hz, 1H), 4.58 (s, 2H), 4.00 – 3.91 (m, 3H), 3.73 (dd, J = 12.2, 4.9 Hz, 1H), 3.58 – 3.50 (m, 1H), 3.44 (s, 1H), 3.36 (d, J = 7.9 Hz, 1H), 2.98 (s, 3H), 2.08 (s, 6H), 1.15 (d, J = 6.1 Hz, 3H). **$^{13}$C NMR** (151 MHz, MeOD) δ 162.59, 162.06, 151.06, 150.56, 149.39, 149.17, 144.57, 144.45, 143.76, 143.70, 136.77, 136.65, 133.60, 131.66, 131.27, 131.06, 128.77, 128.56, 127.19, 125.05, 124.74, 124.50, 124.25, 62.34, 58.66, 57.49, 57.39, 56.57, 49.43, 49.28, 49.14, 49.00, 48.86, 48.72, 48.57, 46.29, 41.09, 15.17, 15.10, 14.13. **LC-MS** (DAD/ESI): $t_R$= 3.32 min, Calcd for $C_{34}H_{40}N_6O_4$ (m/z): $[M+H]^+$ 597.3189; found, $[M+H]^+$ 597.3217, purity: 95%.

**Compound (7)**

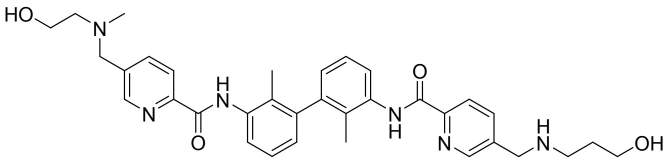

**5-(((2-hydroxyethyl)(methyl)amino)methyl)-N-(3'-(5-(((3-hydroxypropyl)amino)methyl) picolinamido)-2,2'-dimethyl-[1,1'-biphenyl]-3-yl)picolinamide**

*Synthesis of 5-(((2-((tert-butyldimethylsilyl)oxy)ethyl) (methyl)amino)methyl)-N-(3'-(5-(((3-hydroxypropyl)amino)methyl)picolinamido)-2,2'-dimethyl-[1,1'-biphenyl]-3-yl)picolinamide* ***(1.13_7)***

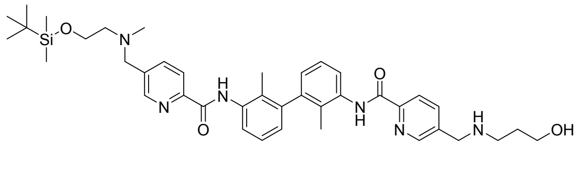

(1.13_7)

**(1.12_2)** (0.2 g, 0.31 mmol, 1 equiv.), 2-Amino-1-propanol (0.117 ml, 1.53 mmol, 5 equiv.), and few drops of AcOH were dissolved in a mixture of DCE/MeOH (10:10, 6 ml) and left for stirring for 2 h. Then $NaBH_3CN$ (0.193 g, 3.07 mmol, 10 equiv.) was added and reaction was left for overnight stirring. After that, water was added and reaction was extracted with EtOAc. The organic layer was washed with brine, dried over $Na_2SO_4$ and evaporated. Crude was purified by flash chromatography using DCM:MeOH (0-20%) to give **(1.13_7)** (0.07 g, 32% yield) as yellowish oil. **$^1$H NMR** (600 MHz, DMSO) δ 10.35 (d, *J* = 2.7 Hz, 2H), 8.69 (s, 1H), 8.68 – 8.64 (m, 1H), 8.14 (dd, *J* = 10.8, 8.0 Hz, 2H), 8.05 – 7.98 (m, 2H), 7.89 – 7.85 (m, 2H), 7.33 (t,

$J$ = 7.8 Hz, 2H), 7.00 (dd, $J$ = 7.9, 2.5 Hz, 2H), 4.10 (q, $J$ = 5.2 Hz, 1H), 3.70 (t, $J$ = 6.0 Hz, 2H), 3.68 (s, 2H), 3.51 – 3.39 (m, 3H), 2.56 (d, $J$ = 11.9 Hz, 5H), 2.23 (s, 3H), 2.02 (s, 7H), 1.63 (dt, $J$ = 17.5, 7.1 Hz, 3H), 0.85 (s, 9H), 0.03 (s, 6H).

*Synthesis of: 5-(((2-hydroxyethyl)(methyl)amino)methyl)-N-(3'-(5-(((3-hydroxypropyl) amino)methyl)picolinamido)-2,2'-dimethyl-[1,1'-biphenyl]-3-yl)picolinamide* ***(7)***

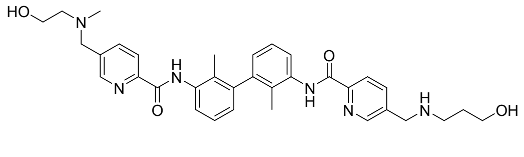

(7)

**(1.13_7)** (0.07 g, 0.1 mmol, 1 equiv.) was dissolved in DCM (2.1 ml) then 6 M HCl in i-PrOH (0.164 ml, 10 equiv.) was added and reaction was left for stirring at RT overnight. Next, solvent was evaporated and residue was dissolved in water. Then 2 M NaOH was added and reaction was extracted with DCM. The organic layer was washed with brine, dried over $Na_2SO_4$ and evaporated. Crude was purified by pTLC using DCM:MeOH (8:2) to give **(7)** (0.03 g, 15% yield) as yellowish solid. **$^1$H NMR** (600 MHz, MeOD) δ 8.66 (ddd, $J$ = 7.8, 2.1, 0.8 Hz, 2H), 8.18 (ddd, $J$ = 8.8, 8.0, 0.8 Hz, 2H), 8.03 – 8.00 (m, 2H), 7.93 (dd, $J$ = 8.1, 1.3 Hz, 2H), 7.31 (t, $J$ = 7.8 Hz, 2H), 7.03 (dt, $J$ = 7.5, 1.3 Hz, 2H), 3.93 (s, 2H), 3.72 – 3.67 (m, 4H), 3.64 (t, $J$ = 6.2 Hz, 2H), 2.77 (t, $J$ = 7.2 Hz, 2H), 2.60 (t, $J$ = 5.9 Hz, 2H), 2.28 (s, 3H), 2.08 (s, 6H), 1.83 – 1.74 (m, 2H). **$^{13}$C NMR** (151 MHz, MeOD) δ 164.51, 164.41, 150.58, 150.08, 150.07, 149.99, 143.73, 139.75, 139.54, 139.43, 139.09, 137.20, 137.16, 130.09, 127.91, 127.11, 123.67, 123.13, 122.99, 61.41, 60.42, 60.32, 60.15, 51.31, 49.85, 49.43, 49.28, 49.14, 49.00, 48.86, 48.72, 48.58, 47.53, 42.72, 32.75, 14.90. **LC-MS** (DAD/ESI): $t_R$= 3.52 min, Calcd for $C_{34}H_{40}N_6O_4$ (m/z): $[M-H]^-$ 595.30; found, $[M-H]^-$ 595.30, purity: 95%.

**Compound (8)**

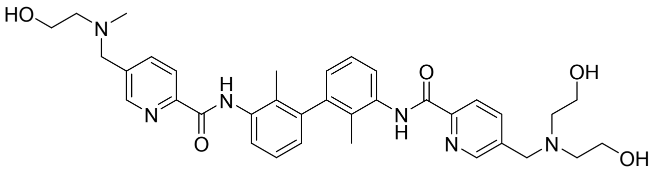

**5-((bis(2-hydroxyethyl)amino)methyl)-N-(3'-(5-(((2-hydroxyethyl)(methyl)amino) methyl)picolinamido)-2,2'-dimethyl-[1,1'-biphenyl]-3-yl)picolinamide**

*Synthesis of 5-((bis(2-hydroxyethyl)amino)methyl)-N-(3'-(5-(((2-((tert-butyldimethylsilyl)oxy)ethyl)(methyl)amino)methyl)picolinamido)-2,2'-dimethyl-[1,1'-biphenyl]-3-yl)picolinamide* ***(1.13_8)***

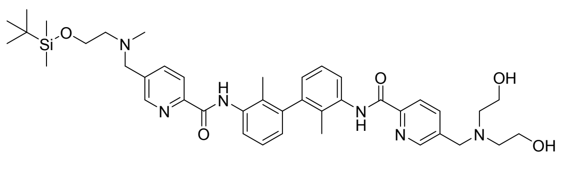

**(1.13_8)**

**(1.12_2)** (0.2 g, 0.31 mmol, 1 equiv.), Diethanolamine (0.088 ml, 0.952 mmol, 3 equiv.), and few drops of AcOH were dissolved in a mixture of DCE/MeOH (20:10, 6 ml) and left for stirring for 2 h. Then NaBH3CN (0.193 g, 3.07 mmol, 10 equiv.) was added and reaction was left for overnight stirring. After that, water was added and reaction was extracted with EtOAc. The organic layer was washed with brine, dried over $Na_2SO_4$ and evaporated. Crude was purified by flash chromatography using DCM:MeOH (0-20%) to give **(1.13_8)** (0.047 g, 21% yield) as yellowish oil. **$^1$H NMR** (600 MHz, DMSO) δ 10.34 (s, 2H), 8.70 – 8.69 (m, 1H), 8.66 – 8.64 (m, 1H), 8.13 (dd, *J* = 8.0, 3.6 Hz, 2H), 8.03 (dd, *J* = 8.0, 2.1 Hz, 1H), 7.99 (dd, *J* = 8.0, 2.1 Hz, 1H), 7.89 (t, *J* = 9.0 Hz, 2H), 7.33 (t, *J* = 7.8 Hz, 2H), 6.99 (d, *J* = 7.5 Hz, 2H), 4.43 (t, *J* = 5.5 Hz, 2H), 4.10 (q, *J* = 5.3 Hz, 1H), 3.81 (s, 2H), 3.70 (t, *J* = 6.0 Hz, 2H), 3.68 (s, 2H), 3.47 (q, *J* = 6.0 Hz, 4H), 2.56 (t, *J* = 6.2 Hz, 4H), 2.44 (s, 3H), 2.23 (s, 3H), 2.02 (d, *J* = 3.6 Hz, 6H), 0.85 (s, 9H), 0.03 (s, 6H).

*Synthesis of 5-((bis(2-hydroxyethyl)amino)methyl)-N-(3'-(5-(((2-hydroxyethyl)(methyl)amino)methyl)picolinamido)-2,2'-dimethyl-[1,1'-biphenyl]-3-yl)picolinamide* ***(8)***

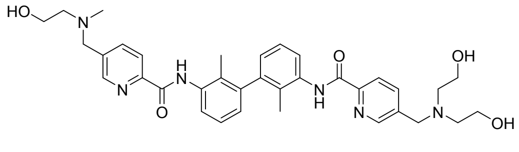

**(8)**

**(1.13_8)** (0.047 g, 0.06 mmol, 1 equiv.) was dissolved in DCM (1.41 ml, 30 equiv.) then 6 M HCl in i-PrOH (0.106 ml) was added and reaction was left for stirring at RT overnight. Next, solvent was evaporated and residue was dissolved in water. Then 2 M NaOH was added and reaction was extracted with DCM. The organic layer was washed with brine, dried over $Na_2SO_4$ and evaporated. Crude was purified by pTLC using DCM:MeOH (8:2) to give **(8)** (0.03 g, 15% yield) as yellowish solid. **$^1$H NMR** (400 MHz, MeOD) δ 8.65 (dd, J = 11.4, 2.0 Hz, 2H), 8.15 (dd, J = 8.0, 5.2 Hz, 2H), 8.01 (ddd, J = 14.7, 8.0, 2.1 Hz, 2H), 7.93 (dd, J = 8.1, 1.3 Hz, 2H), 7.30 (d, J = 7.9 Hz, 2H), 7.00 (dd, J = 7.7, 1.3 Hz, 2H), 3.83 (s, 2H), 3.74 – 3.66 (m, 4H), 3.63 (t, J = 5.7 Hz, 4H), 3.35 (s, 2H), 2.68 (t, J = 5.8 Hz, 4H), 2.59 (t, J = 5.9 Hz, 2H), 2.27 (s, 3H), 2.06 (s, 6H). **$^{13}$C NMR** (101 MHz, MeOD) δ 164.46, 164.39, 150.55, 150.35, 149.96, 149.77, 143.67, 140.54, 139.74, 139.48, 139.34, 137.16, 129.88, 129.84, 127.83, 127.80, 127.11, 60.74,

60.38, 60.28, 60.11, 57.57, 57.49, 42.71, 25.25, 14.94. **LC-MS** (DAD/ESI): $t_R$= 3.49 min, Calcd for $C_{35}H_{42}N_6O_5$ (m/z): $[M+H]^+$ 627.33; found, $[M+H]^+$ 627.46, purity: 98%.

**Compound (3)**

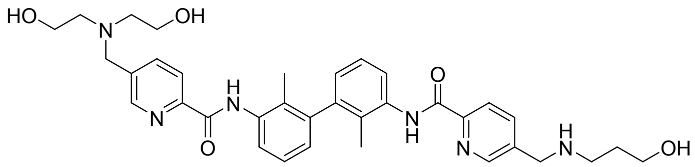


**5-((bis(2-hydroxyethyl)amino)methyl)-N-(3'-(5-(((3-hydroxypropyl)amino)methyl)picolinamido)-2,2'-dimethyl-[1,1'-biphenyl]-3-yl) picolinamide**

*Synthesis of tert-butyl (3'-(5-((bis(2-hydroxyethyl)amino) methyl)picolinamido)-2,2'-dimethyl-[1,1'-biphenyl]-3-yl)carbamate* ***(1.7_3)***

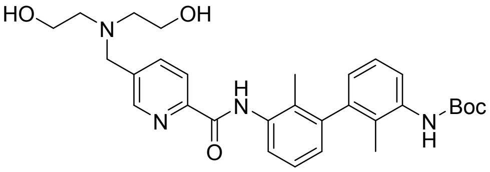


**(1.7_3)**

**(1.6_2)** (0.65 g, 1.27 mmol, 1 equiv.) was dissolved in ACN (13 ml), then $K_2CO_3$ (0.528 g, 3.82 mmol, 3 equiv.), KI (0.211 g, 1.27 mmol, 1 equiv.) and Diethanolamine (0.366 ml, 3.82 mmol, 3 equiv.) were added. Mixture was left for stirring at 50°C overnight. Next, water was added and reaction was extracted with DCM. The organic layer was washed with brine, dried over $Na_2SO_4$ and evaporated to give **(1.7_3)** (0.7 g) as yellowish foam. **$^1$H NMR** (300 MHz, DMSO) δ 10.31 (s, 1H), 8.69 (s, 1H), 8.58 (s, 1H), 8.13 (d, $J$ = 8.0 Hz, 1H), 8.07 – 8.01 (m, 1H), 7.87 (d, $J$ = 8.0 Hz, 1H), 7.32 (t, $J$ = 7.5 Hz, 2H), 7.21 (t, $J$ = 7.8 Hz, 1H), 6.92 (t, $J$ = 8.5 Hz, 2H), 4.41 (t, $J$ = 5.4 Hz, 2H), 3.81 (s, 2H), 3.47 (q, $J$ = 6.0 Hz, 4H), 2.61 – 2.55 (m, 4H), 1.98 (s, 3H), 1.89 (s, 3H), 1.46 (s, 9H).

*Synthesis of N-(3'-amino-2,2'-dimethyl-[1,1'-biphenyl]-3-yl)-5-((bis(2-hydroxyethyl)amino) methyl)picolinamide* ***(1.8_3)***

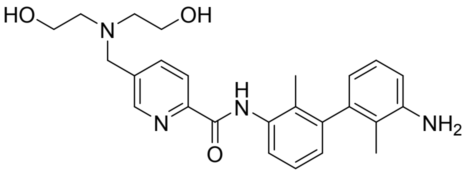


**(1.8_3)**

**(1.7_3)** (0.7 g, 1.31 mmol, 1 equiv.) was dissolved in DCM (21 ml) then 6 M HCl in i-PrOH (2.182 ml, 10 equiv.) was added and reaction was left for stirring at RT overnight. Next, solvent was evaporated and residue was dissolved in water. Then 2 M NaOH was added and

reaction was extracted with DCM. The organic layer was washed with brine, dried over $Na_2SO_4$ and evaporated to give **(1.8_3)** (0.514 g, 90% yield) as colorless oil. **$^1$H NMR** (300 MHz, DMSO) δ 10.28 (s, 1H), 8.69 (d, *J* = 2.0 Hz, 1H), 8.16 – 8.11 (m, 1H), 8.03 (dd, *J* = 8.0, 2.2 Hz, 1H), 7.86 (dd, *J* = 8.1, 1.5 Hz, 1H), 7.26 (t, *J* = 7.8 Hz, 1H), 6.96 – 6.88 (m, 2H), 6.65 (dt, *J* = 7.9, 1.8 Hz, 1H), 6.31 (dd, *J* = 7.5, 1.3 Hz, 1H), 4.89 (s, 2H), 4.44 – 4.39 (m, 2H), 3.80 (d, *J* = 2.5 Hz, 2H), 3.52 – 3.43 (m, 4H), 3.30 (s, 2H), 2.62 – 2.54 (m, 2H), 1.99 (d, *J* = 2.8 Hz, 3H), 1.74 (d, *J* = 2.4 Hz, 3H).

*Synthesis of N-(3'-amino-2,2'-dimethyl-[1,1'-biphenyl]-3-yl)-5-((bis(2-((tert-butyldimethylsilyl)oxy)ethyl) amino)methyl)picolinamide* ***(1.9_3)***

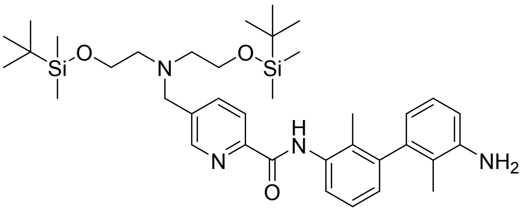

**(1.9_3)**

**(1.8_3)** (0.514 g, 1.18 mmol, 1 equiv.) was dissolved in DMF (7.71 ml) and cooled on an ice bath. Imidazole (0.362 g, 5.32 mmol, 4.5 equiv.) was then added slowly (over ˜10 minutes). A solution of TBDMS-Cl (0.41 g, 2.72 mmol, 2.3 equiv.) in DMF (2.05 ml, 5 equiv.) was added (over ˜2 minutes). The ice bath was removed and the solution was stirred at room temperature for 1 h (no SM). Then water was added and reaction was extracted with EtOAc. The organic layer was washed with brine, dried over $Na_2SO_4$ and evaporated to give **(1.9_3)** (0.45 g, 57% yield) as yellowish oil. **$^1$H NMR** (600 MHz, DMSO) δ 10.29 (s, 1H), 8.67 (dd, *J* = 2.0, 0.8 Hz, 1H), 8.11 (dd, *J* = 7.9, 0.8 Hz, 1H), 8.00 (dd, *J* = 8.0, 2.0 Hz, 1H), 7.86 (dd, *J* = 8.1, 1.3 Hz, 1H), 7.27 (d, *J* = 7.8 Hz, 1H), 6.95 – 6.90 (m, 2H), 6.65 (dd, *J* = 8.0, 1.3 Hz, 1H), 6.31 (dd, *J* = 7.4, 1.3 Hz, 1H), 4.91 (s, 2H), 3.85 (s, 2H), 3.64 (t, *J* = 6.1 Hz, 4H), 2.64 (t, *J* = 6.1 Hz, 4H), 1.74 (s, 3H), 0.83 (s, 18H), 0.00 (s, 12H).

*Synthesis of methyl 6-((3'-(5-((bis(2-((tert-butyldimethylsilyl)oxy)ethyl)amino)methyl) picolinamido)-2,2'-dimethyl-[1,1'-biphenyl]-3-yl)carbamoyl)nicotinate* ***(1.10_3)***

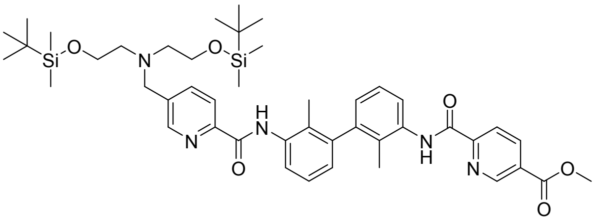

**(1.10_3)**

To a solution of 5-(Methoxycarbonyl)picolinic acid (0.123 g, 0.68 mmol, 1 equiv.) in DMF (13.5 ml) was added TEA (0.189 ml, 1.36 mmol, 2 equiv.). The mixture was cooled to 0oC and treated

with HATU (0.516 g, 1.36 mmol, 2 equiv.) and the **(1.9_3)** (0.45 g, 0.68 mmol, 1 equiv.). The reaction was stirred at RT overnight. Then water was added and reaction was extracted with DCM. The organic layer was washed with brine, dried over $Na_2SO_4$ and evaporated. Crude was purified by flash chromatography using DCM:MeOH (8%) to give **(1.10_3)** (0.265 g, 47% yield) as yellowish solid. **$^1$H NMR** (600 MHz, DMSO) δ 10.49 (s, 1H), 10.34 (s, 1H), 9.19 (dd, *J* = 2.1, 0.9 Hz, 1H), 8.69 – 8.66 (m, 1H), 8.56 (dd, *J* = 8.1, 2.1 Hz, 1H), 8.31 (dd, *J* = 8.2, 0.9 Hz, 1H), 8.13 – 8.10 (m, 1H), 8.01 (dd, *J* = 8.0, 2.1 Hz, 1H), 7.90 (d, *J* = 8.0 Hz, 1H), 7.76 (d, *J* = 8.0 Hz, 1H), 7.33 (td, *J* = 7.7, 2.7 Hz, 2H), 7.03 (dd, *J* = 7.7, 1.3 Hz, 1H), 6.99 (dd, *J* = 7.6, 1.3 Hz, 1H), 3.94 (s, 3H), 3.85 (s, 2H), 3.67 – 3.60 (m, 4H), 2.64 (t, *J* = 6.1 Hz, 4H), 2.01 (d, *J* = 5.4 Hz, 6H), 0.83 (s, 18H), 0.00 (s, 12H).

*Synthesis of 5-((bis(2-((tert-butyldimethylsilyl)oxy)ethyl) amino)methyl)-N-(3'-(5-(hydroxymethyl)picolinamido)-2,2'-dimethyl-[1,1'-biphenyl]-3-yl)picolinamide **(1.11_3)***

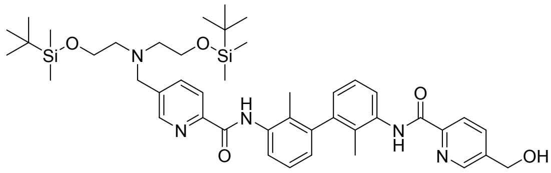


(1.11_3)

**(1.10_3)** (0.265 g, 0.32 mmol, 1 equiv.) was dissolved in THF/MeOH (10:5, 2.65 ml:1.33 ml) and cooled to 0°C. Then solution of 4M $LiBH_4$ in THF (0.401 ml, 1.6 ml, 5 equiv.) was added dropwise and reaction was stirred at this temperature for 15 min and then left to warm to RT. Reaction was monitored by TLC. After full consumption of SM (15 min) water was added and solvent was evaporated. To the residue DCM was added and reaction was extracted with DCM/water. The organic layer was washed with brine, dried over $Na_2SO_4$ and evaporated to give **(1.11_3)** (0.25 g, 98% yield) as yellowish oil. **$^1$H NMR** (600 MHz, DMSO) δ 10.34 (d, *J* = 5.4 Hz, 2H), 8.67 (ddd, *J* = 7.2, 2.1, 0.9 Hz, 2H), 8.15 (dd, *J* = 8.0, 0.8 Hz, 1H), 8.12 (dd, *J* = 7.9, 0.8 Hz, 1H), 8.00 (td, *J* = 7.8, 2.1 Hz, 2H), 7.92 – 7.87 (m, 2H), 7.33 (t, *J* = 7.8 Hz, 2H), 6.99 (dt, *J* = 7.6, 1.8 Hz, 2H), 5.51 (t, *J* = 5.7 Hz, 1H), 4.66 (d, *J* = 5.6 Hz, 2H), 3.85 (s, 2H), 3.65 (t, *J* = 6.1 Hz, 4H), 2.64 (t, *J* = 6.1 Hz, 4H), 2.02 (d, *J* = 1.5 Hz, 6H), 0.83 (s, 18H), 0.00 (s, 12H).

*Synthesis of 5-((bis(2-((tert-butyldimethylsilyl)oxy) ethyl)amino)methyl)-N-(3'-(5-formylpicolinamido)-2,2'-dimethyl-[1,1'-biphenyl]-3-yl)picolinamide **(1.12_3)***

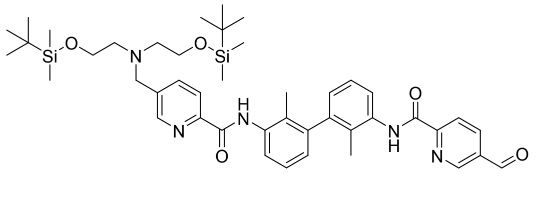

(1.12_3)

**(1.11_3)** (0.2 g, 0.25 mmol, 1 equiv.) was dissolved in DCM and cooled to 0°C. Then, Dess-Martin periodinane (0.213 g, 0.5 mmol, 2 equiv.) and $NaHCO_3$ (0.105 g, 1.25 mmol, 5 equiv.) was added and reaction was stirred for 0.5 h at 0°C. Next reaction was left warm to RT and stirred for 30 min (no SM). After that time $NaHCO_3$ was added and reaction was extracted with water. The organic layer was washed with brine, dried over $Na_2SO_4$ and evaporated. Crude was purified by flash chromatography using Hex:EtOAc (0-100%) to give **(1.12_3)** (0.14 g, 70% yield) as yellowish oil. **$^1$H NMR** (300 MHz, DMSO) δ 10.49 (s, 1H), 10.32 (s, 1H), 10.23 (d, $J$ = 2.4 Hz, 1H), 9.22 (d, $J$ = 2.2 Hz, 1H), 8.67 (s, 1H), 8.51 (dt, $J$ = 8.1, 2.3 Hz, 1H), 8.35 (dd, $J$ = 8.2, 2.4 Hz, 1H), 8.14 – 8.09 (m, 1H), 8.00 (d, $J$ = 8.2 Hz, 1H), 7.90 (d, $J$ = 8.0 Hz, 1H), 7.79 (d, $J$ = 7.8 Hz, 1H), 7.33 (t, $J$ = 7.8 Hz, 2H), 7.05 – 6.96 (m, 2H), 3.85 (s, 2H), 3.65 (t, $J$ = 5.9 Hz, 4H), 2.65 (t, $J$ = 6.3 Hz, 4H), 2.02 (d, $J$ = 2.3 Hz, 6H), 0.83 (d, $J$ = 2.5 Hz, 18H), 0.01 (s, 12H).

*Synthesis of 5-((bis(2-((tert-butyldimethylsilyl)oxy)ethyl)amino)methyl)-N-(3'-(5-(((3-hydroxypropyl)amino)methyl) picolinamido)-2,2'-dimethyl-[1,1'-biphenyl]-3-yl)picolinamide **(1.13_3)***

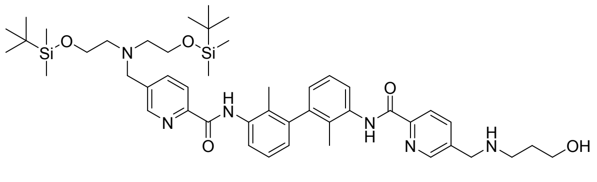

(1.13_3)

**(1.12_3)** (0.14 g, 0.18 mmol, 1 equiv.), 3-Amino-1-propanol (0.054 ml, 0.7 mmol, 4 equiv.), and few drops of AcOH were dissolved in a mixture of DCE/MeOH (20:10, 2.8 ml:1.4 ml) and left for stirring for 2 h. Then $NaBH_3CN$ (0.088 g, 1.41 mmol, 8 equiv.) was added and reaction was left for overnight stirring. After that, water was added and reaction was extracted with EtOAc. The organic layer was washed with brine, dried over $Na_2SO_4$ and evaporated. Crude was purified by flash chromatography using DCM:MeOH (0-20%) to give **(1.13_3)** (0.077 g, 51% yield) as yellowish oil. **$^1$H NMR** (600 MHz, DMSO) δ 10.34 (d, $J$ = 5.0 Hz, 2H), 8.67 (dd, $J$ = 8.4, 2.1 Hz, 2H), 8.12 (t, $J$ = 7.9 Hz, 2H), 8.01 (ddd, $J$ = 7.3, 4.8, 2.1 Hz, 2H), 7.91 – 7.87 (m, 2H), 7.33 (t, $J$ = 7.6 Hz, 2H), 6.99 (d, $J$ = 7.4 Hz, 2H), 4.59 – 4.52 (m, 0H), 3.85 (s, 2H), 3.81 (s, 2H), 3.65 (t, $J$ = 6.1 Hz, 4H), 3.46 (t, $J$ = 6.3 Hz, 1H), 3.42 (d, $J$ = 5.3 Hz, 1H), 2.64 (t, $J$ = 6.1 Hz, 4H), 2.55 (t, $J$ = 6.9 Hz, 2H), 2.02 (d, $J$ = 2.0 Hz, 6H), 1.58 (q, $J$ = 6.6 Hz, 1H), 0.83 (s, 18H), 0.00 (s, 12H).

*Synthesis of 5-((bis(2-hydroxyethyl)amino)methyl)-N-(3'-(5-(((3-hydroxypropyl)amino)methyl) picolinamido)-2,2'-dimethyl-[1,1'-biphenyl]-3-yl) picolinamide* ***(3)***

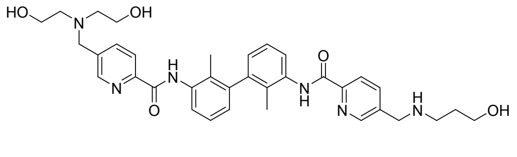

(3)

**(1.13_3)** (0.077 g, 0.09 mmol, 1 equiv.) was dissolved in DCM (2.31 ml) then 6 M HCl in i-PrOH (0.15 ml, 0.9 mmol, 10 equiv.) was added and reaction was left for stirring at RT overnight. Next, solvent was evaporated, and residue was dissolved in water. Then 2 M NaOH was added, and reaction was extracted with DCM. The organic layer was washed with brine, dried over $Na_2SO_4$ and evaporated. Crude was purified by pTLC using DCM:MeOH (6:4) to give **(3)** (0.005 g, 9% yield) as yellowish solid. **$^1$H NMR** (600 MHz, DMSO) δ 10.34 (s, 2H), 8.70 (d, $J$ = 2.0 Hz, 1H), 8.66 (s, 1H), 8.13 (d, $J$ = 7.9 Hz, 2H), 8.03 (dd, $J$ = 8.1, 2.0 Hz, 1H), 8.00 (dd, $J$ = 7.9, 2.1 Hz, 1H), 7.89 (t, $J$ = 7.1 Hz, 2H), 7.33 (t, $J$ = 7.8 Hz, 2H), 6.99 (dd, $J$ = 7.5, 1.3 Hz, 2H), 4.43 (t, $J$ = 5.5 Hz, 2H), 3.81 (d, $J$ = 2.7 Hz, 4H), 3.47 (p, $J$ = 6.1 Hz, 6H), 2.56 (t, $J$ = 6.2 Hz, 4H), 2.02 (d, $J$ = 2.2 Hz, 6H), 1.59 (q, $J$ = 6.7 Hz, 2H), 1.25 (d, $J$ = 5.5 Hz, 2H), 0.90 – 0.83 (m, 2H). **$^{13}$C NMR** (101 MHz, METHANOL-D4) δ 164.24, 164.02, 150.27, 150.08, 149.65, 143.55, 143.52, 140.40, 139.44, 139.21, 138.68, 137.12, 137.06, 129.54, 129.49, 127.71, 127.64, 127.08, 123.19, 123.08, 122.89, 61.22, 60.65, 57.46, 57.38, 51.04, 49.64, 49.43, 49.21, 49.00, 48.79, 48.57, 48.36, 47.47, 32.37, 14.91. **LC-MS** (DAD/ESI): $t_R$= 3.48 min, Calcd for $C_{35}H_{42}N_6O_5$ (m/z): $[M+H]^+$ 627.33; found, $[M+H]^+$ 627.43, purity: 100%.

**Compound (4)**

**5-((bis(2-hydroxyethyl)amino)methyl)-N-(3'-(5-((((1R,2R)-2-hydroxycyclopentyl)amino) methyl)picolinamido)-2,2'-dimethyl-[1,1'-biphenyl]-3-yl)picolinamide**

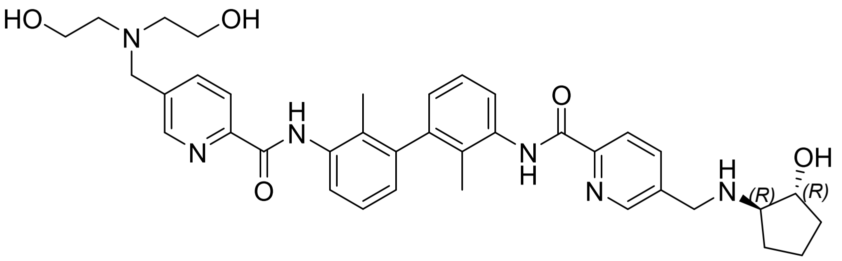

*Synthesis of 5-((bis(2-((tert-butyldimethylsilyl)oxy)ethyl) amino)methyl)-N-(3'-(5-(chloromethyl)picolinamido)-2,2'-dimethyl-[1,1'-biphenyl]-3-yl)picolinamide* ***(1.12_4)***

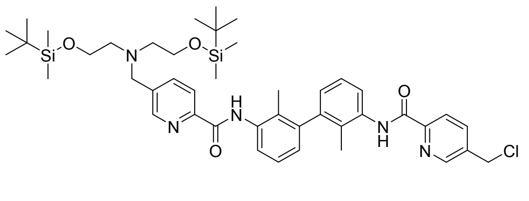

(1.12_4)

**(1.11_3)** (0.05 g, 0.06 mmol, 1 equiv.) was dissolved in DCM (0.5 ml) and cooled to 0°C. Then TEA (0.026 ml, 0.19 mmol, 3 equiv.) and methanesulfonic chloride (0.007 ml, 0.09 mmol, 1 equiv.) was added and reaction was stirred for 30 min (no SM). Then water was added, and reaction was extracted with DCM, washed with brine, dried over $Na_2SO_4$ and evaporated to give **(1.12_4)** (0.041 g, 80% yield) as yellowish oil. **$^1$H NMR** (300 MHz, DMSO) δ 10.37 (s, 1H), 10.32 (s, 1H), 8.81 (s, 1H), 8.68 (s, 1H), 8.27 – 8.10 (m, 4H), 8.01 (d, *J* = 8.4 Hz, 1H), 7.88 (dd, *J* = 18.0, 8.0 Hz, 2H), 7.33 (t, *J* = 7.8 Hz, 3H), 7.00 (t, *J* = 6.7 Hz, 3H), 5.46 (s, 2H), 3.85 (s, 2H), 3.65 (t, *J* = 6.1 Hz, 4H), 2.65 (t, *J* = 6.0 Hz, 4H), 2.02 (s, 6H), 0.83 (d, *J* = 1.0 Hz, 18H), 0.00 (d, *J* = 1.0 Hz, 12H).

*Synthesis of 5-((bis(2-((tert-butyldimethylsilyl)oxy)ethyl) amino)methyl)-N-(3'-(5-((((1R,2R)-2-hydroxycyclopentyl)amino)methyl)picolinamido)-2,2'-dimethyl-[1,1'-biphenyl]-3-yl) picolinamide **(1.13_4)***

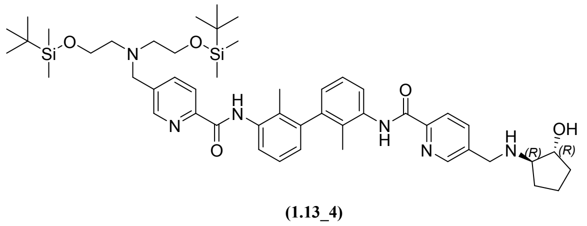

(1.13_4)

**(1.12_4)** (0.041 g, 0.05 mmol, 1 equiv.) was dissolved in ACN (0.82 ml), then $K_2CO_3$ (0.028 g, 0.2 mmol, 4 equiv.) KI (0.008 g, 0.05 mmol, 1 equiv.) and (1R,2R)-trans-2-Aminocyclopentanol hydrochloride (0.014 g, 0.1 mmol, 2 equiv.) were added. Mixture was left for stirring at 50°C overnight. Next, water was aded and reaction was extracted with DCM. The organic layer was washed with brine, dried over $Na_2SO_4$ and evaporated. Crude was purified by pTLC using DCM:MeOH (8:2) to give **(1.13_4)** (0.027 g, 61% yield) as yellowish oil. **$^1$H NMR** (600 MHz, DMSO) δ 10.34 (s, 2H), 8.67 (t, *J* = 2.6 Hz, 2H), 8.16 – 8.11 (m, 1H), 8.04 – 8.00 (m, 2H), 7.92 – 7.89 (m, 2H), 7.33 (t, *J* = 7.8 Hz, 2H), 6.99 (d, *J* = 7.5 Hz, 2H), 4.52 (d, *J* = 4.2 Hz, 1H), 4.10 (q, *J* = 5.3 Hz, 2H), 3.85 (d, *J* = 6.9 Hz, 4H), 3.79 (p, *J* = 4.4 Hz, 1H), 3.65 (t, *J* = 6.1 Hz, 4H), 2.64 (t, *J* = 6.1 Hz, 4H), 2.02 (d, *J* = 2.7 Hz, 6H), 1.84 – 1.77 (m, 2H), 1.61 – 1.55 (m, 2H), 1.40 (dt, *J* = 13.1, 6.2 Hz, 1H), 1.30 – 1.25 (m, 1H), 0.83 (s, 18H), 0.00 (s, 12H).

*Synthesis of 5-((bis(2-hydroxyethyl)amino)methyl)-N-(3'-(5-((((1R,2R)-2-hydroxycyclopentyl) amino)methyl)picolinamido)-2,2'-dimethyl-[1,1'-biphenyl]-3-yl)picolinamide **(4)***

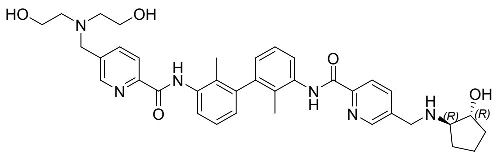

(4)

**(1.13_4)** (0.027 g, 0.03 mmol, 1 equiv.) was dissolved in DCM (0.81 ml) then 6 M HCl in i-PrOH (0.051 ml, 10 equiv.) was added and reaction was left for stirring at RT overnight. Next, solvent was evaporated and residue was dissolved in water. Then 2 M NaOH was added and reaction was extracted with DCM. The organic layer was washed with brine, dried over $Na_2SO_4$ and evaporated. Crude was purified by pTLC using DCM:MeOH (8:2) to give **(4)** (0.03 g, 15% yield) as yellowish solid. **$^1$H NMR** (600 MHz, MeOD) δ 8.71 (d, *J* = 2.1 Hz, 2H), 8.23 – 8.17 (m, 2H), 8.06 (td, *J* = 8.2, 2.2 Hz, 2H), 7.92 (d, *J* = 8.1 Hz, 2H), 7.72 (dd, *J* = 5.7, 3.3 Hz, 1H), 7.63 (dd, *J* = 5.8, 3.3 Hz, 1H), 7.34 (t, *J* = 7.8 Hz, 2H), 7.06 (dt, *J* = 7.6, 1.6 Hz, 2H), 4.22 (ddd, *J* = 5.7, 4.3, 1.2 Hz, 1H), 4.01 – 3.97 (m, 2H), 3.64 (t, *J* = 5.8 Hz, 4H), 2.94 (q, *J* = 7.3 Hz, 1H), 2.70 (t, *J* = 5.8 Hz, 4H), 2.10 (s, 6H), 2.06 (td, *J* = 14.0, 13.2, 8.5 Hz, 2H), 1.98 (dt, *J* = 14.2, 7.2 Hz, 1H), 1.74 – 1.67 (m, 1H), 1.57 (dq, *J* = 13.8, 6.3 Hz, 1H), 1.46 – 1.42 (m, 1H), 1.40 – 1.36 (m, 1H). **$^{13}$C NMR** (101 MHz, METHANOL-D4) δ 164.28, 164.16, 150.28, 150.01, 149.82, 149.70, 143.59, 143.58, 140.48, 139.96, 139.43, 139.02, 137.15, 137.12, 129.56, 127.71, 127.69, 127.09, 123.23, 123.04, 122.91, 78.66, 66.83, 60.71, 57.54, 57.45, 49.64, 49.42, 49.19, 48.98, 48.78, 48.56, 48.36, 34.08, 30.97, 21.84, 14.92. **LC-MS** (DAD/ESI): $t_R$= 3.67 min, Calcd. for $C_{37}H_{44}N_6O_5$ (m/z): $[M+H]^+$ 653.35; found, $[M+H]^+$ 653.38, purity: 98%.

**Compound (5)**

**5-(((1,3-dihydroxypropan-2-yl)(methyl)amino)methyl)-N-(3'-(5-(((3-hydroxypropyl)amino)methyl)picolinamido)-2,2'-dimethyl-[1,1'-biphenyl]-3-yl)picolinamide**

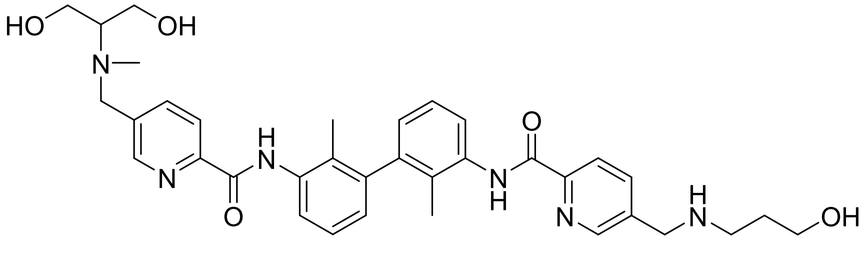

*Synthesis of tert-butyl (3'-(5-(((1,3-dihydroxypropan-2-yl)amino)methyl)picolinamido)-2,2'-dimethyl-[1,1'-biphenyl]-3-yl)carbamate **(1.7_5)***

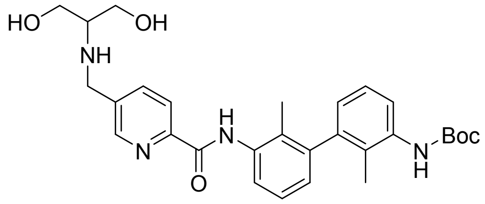

(1.7_5)

**(1.6_2)** (0.7 g, 1.37 mmol, 1 equiv.) was dissolved in ACN (14 ml), then $K_2CO_3$ (0.569 g, 4.11 mmol, 3 equiv.), KI (0.228 g, 1.37 mmol, 1 equiv.) and 2-Aminopropane-1,3-diol (0.375 g, 4.11 mmol, 3 equiv.) were added. Mixture was left for stirring at 50$^o$C overnight. Next, water was aded and reaction was extracted with DCM. The organic layer was washed with brine, dried over $Na_2SO_4$ and evaporated to give **(1.7_5)** (0.65 g, 91% yield) as yellowish foam. **$^1$H NMR** (300 MHz, DMSO) δ 10.31 (s, 1H), 8.70 – 8.67 (m, 1H), 8.12 (dd, *J* = 8.0, 0.9 Hz, 1H), 8.03 (dd, *J* = 8.1, 2.0 Hz, 1H), 7.89 – 7.83 (m, 1H), 7.32 (t, *J* = 7.5 Hz, 2H), 7.21 (t, *J* = 7.8 Hz, 1H), 6.92 (ddd, *J* = 9.0, 7.5, 1.4 Hz, 2H), 4.43 (t, *J* = 5.4 Hz, 2H), 3.93 (s, 2H), 3.50 – 3.34 (m, 4H), 1.98 (d, *J* = 3.0 Hz, 3H), 1.89 (s, 3H), 1.46 (d, *J* = 0.8 Hz, 9H).

*Synthesis of tert-butyl (2,2'-dimethyl-3'-(5-(((2,2,3,3,9,9,10,10-octamethyl-4,8-dioxa-3,9-disilaundecan-6-yl)amino)methyl) picolinamido)-[1,1'-biphenyl]-3-yl)carbamate **(1.8_5)***

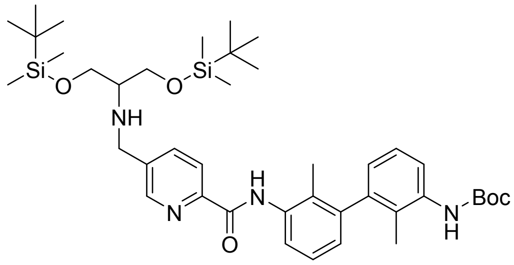

(1.8_5)

**(1.7_5)** (0.65 g, 1.25 mmol, 1 equiv.) was dissolved in DMF (9.75 ml) and cooled on an ice bath. Imidazole (0.382 g, 5.62 mmol, 4.5 equiv.) was then added slowly (over ˜10 minutes). A solution of TBDMS-Cl (0.433 g, 2.87 mmol, 2.3 ml) in DMF (2.16 ml, 5 equiv.) was added (over ˜2 minutes). The ice bath was removed and the solution was stirred at room temperature for 1 h (no SM). Then water was added and reaction was extracted with EtOAc. The organic layer was washed with brine, dried over $Na_2SO_4$ and evaporated to give **(1.8_5)** (1.13 g) as yellowish oil. **$^1$H NMR** (300 MHz, DMSO) δ 10.30 (s, 1H), 8.67 (d, *J* = 2.4 Hz, 1H), 8.58 (s, 1H), 8.12 – 8.10 (m, 1H), 8.01 (dd, *J* = 7.9, 2.3 Hz, 1H), 7.87 (d, *J* = 8.0 Hz, 1H), 7.32 (dd, *J* = 9.8, 4.5 Hz, 1H), 7.23 – 7.16 (m, 1H), 6.92 (td, *J* = 8.2, 7.4, 1.4 Hz, 2H), 3.95 (s, 1H), 3.63 – 3.52 (m, 4H), 1.97 (d, *J* = 3.0 Hz, 3H), 1.88 (d, *J* = 3.0 Hz, 3H), 1.45 (d, *J* = 3.2 Hz, 9H), 0.86 – 0.82 (m, 18H), 0.01 (dd, *J* = 3.4, 1.0 Hz, 12H).

*Synthesis of tert-butyl (2,2'-dimethyl-3'-(5-((methyl(2,2,3,3,9,9,10,10-octamethyl-4,8-dioxa-3,9-disilaundecan-6-yl)amino)methyl)picolinamido)-[1,1'-biphenyl]-3-yl)carbamate **(1.9_5)***

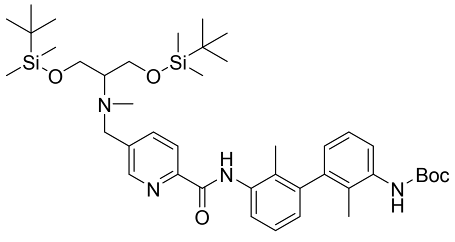

(1.9_5)

**(1.8_5)** (1.13 g, 1.51 mmol, 1 equiv.) was dissolved in ACN (22.6 ml), then $K_2CO_3$ (0.625 g, 4.53 mmol, 3 equiv.), and $CH_3I$ (0.188 ml, 3.02 mmol, 2 equiv.) were added. Mixture was left for stirring at 50°C for 1 h and then at RT overnight. Next, water was added and reaction was extracted with DCM. The organic layer was washed with brine, dried over $Na_2SO_4$ and evaporated. Crude was purified by flash chromatography using Hex:EtOAc (0-100%) to give **(1.9_5)** (0.36 g, 31% yield) as yellowish oil. **$^1$H NMR** (600 MHz, DMSO) δ 10.32 (s, 1H), 8.67 – 8.60 (m, 2H), 8.12 (d, *J* = 7.9 Hz, 1H), 7.97 (dd, *J* = 8.0, 2.0 Hz, 1H), 7.87 (dd, *J* = 8.1, 1.3 Hz, 1H), 7.31 (dt, *J* = 15.5, 8.0 Hz, 2H), 7.21 (t, *J* = 7.8 Hz, 1H), 6.92 (ddd, *J* = 18.5, 7.6, 1.3 Hz, 2H), 3.90 (s, 2H), 3.79 – 3.70 (m, 4H), 2.72 (p, *J* = 5.9 Hz, 1H), 2.29 (s, 3H), 1.97 (s, 3H), 1.89 (s, 3H), 1.46 (s, 9H), 0.87 (s, 18H), 0.04 (d, *J* = 1.1 Hz, 12H).

*Synthesis of N-(3'-amino-2,2'-dimethyl-[1,1'-biphenyl]-3-yl)-5-(((1,3-dihydroxypropan-2-yl)(methyl)amino)methyl)picolinamide **(1.10_5)***

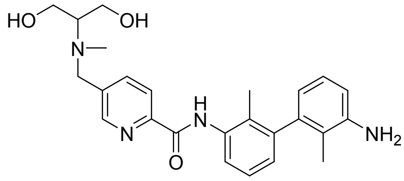

(1.10_5)

**(1.9_5)** (0.36 g, 0.47 mmol, 1 equiv.) was dissolved in DCM (10.8 ml) then 6 M HCl in i-PrOH (0.786 ml, 10 equiv.) was added and reaction was left for stirring at RT overnight. Next, solvent was evaporated and residue was dissolved in water. Then 2 M NaOH was added and reaction was extracted with DCM. The organic layer was washed with brine, dried over $Na_2SO_4$ and evaporated to give **(1.10_5)** (0.2 g) as yellowish oil.

*Synthesis of N-(3'-amino-2,2'-dimethyl-[1,1'-biphenyl]-3-yl)-5-((methyl(2,2,3,3,9,9,10,10-octamethyl-4,8-dioxa-3,9-disilaundecan-6-yl)amino)methyl)picolinamide **(1.11_5)***

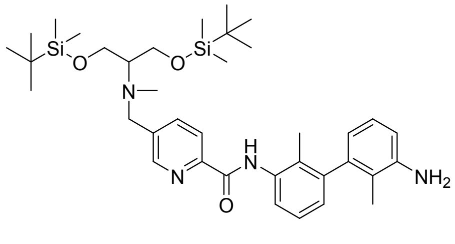

(1.11_5)

**(1.10_5)** (0.2 g, 0.46 mmol, 1 equiv.) was dissolved in DMF (3 ml) and cooled on an ice bath. Imidazole (0.141 g, 2.07 mmol, 4.5 equiv.) was then added slowly (over ˜10 minutes). A solution of TBDMS-Cl (0.16 g, 1.06 mmol, 2.3 equiv.) in DMF (0.8 ml, 5 equiv.) was added (over ˜2 minutes). The ice bath was removed and the solution was stirred at room temperature for 1 h (no SM). Then water was added and reaction was extracted

with EtOAc. The organic layer was washed with brine, dried over $Na_2SO_4$ and evaporated to give **(1.11_5)** (0.32 g) as yellowish oil. **$^1$H NMR** (600 MHz, DMSO) δ 10.29 (s, 1H), 8.65 – 8.60 (m, 1H), 8.12 (dd, *J* = 7.9, 0.8 Hz, 1H), 7.99 – 7.92 (m, 2H), 7.86 (dd, *J* = 8.1, 1.3 Hz, 1H), 7.25 (d, *J* = 7.8 Hz, 1H), 6.96 – 6.90 (m, 2H), 6.65 (dd, *J* = 8.0, 1.3 Hz, 1H), 6.31 (dd, *J* = 7.4, 1.3 Hz, 1H), 4.91 (s, 2H), 3.89 (s, 2H), 3.77 – 3.70 (m, 4H), 2.29 (s, 3H), 1.99 (s, 3H), 1.74 (s, 3H), 0.87 (s, 18H), 0.04 (d, *J* = 1.1 Hz, 12H).

*Synthesis of methyl 6-((2,2'-dimethyl-3'-(5-((methyl(2,2,3,3,9,9,10,10-octamethyl-4,8-dioxa-3,9-disilaundecan-6-yl)amino)methyl)picolinamido)-[1,1'-biphenyl]-3yl)carbamoyl)nicotinate* ***(1.12_5)***

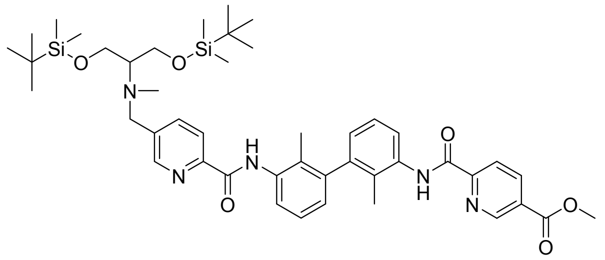


**(1.12_5)**

To a solution of 5-(Methoxycarbonyl)picolinic acid (0.087 g, 0.48 mmol, 1 equiv.) in DMF (9.6 ml) was added TEA (0.135 ml, 0.97 mmol, 2 equiv.). The mixture was cooled to 0°C and treated with HATU (0.367 g, 0.97 mmol, 2 equiv.) and the **(1.11_5)** (0.32 g, 0.48 mmol, 1 equiv.). The reaction was stirred at RT overnight. Then water was added and reaction was extracted with DCM. The organic layer was washed with brine, dried over $Na_2SO_4$ and evaporated. Crude was purified by flash chromatography using Hex:DCM:MeOH (8%) to give **(1.12_5)** (0.24 g, 73% yield) as yellowish solid. **$^1$H NMR** (600 MHz, DMSO) δ 10.49 (s, 1H), 10.33 (s, 1H), 9.19 (dd, *J* = 2.1, 0.8 Hz, 1H), 8.63 (s, 1H), 8.56 (dd, *J* = 8.1, 2.1 Hz, 1H), 8.31 (dd, *J* = 8.1, 0.9 Hz, 1H), 8.12 (d, *J* = 8.0 Hz, 1H), 7.98 (dd, *J* = 8.1, 2.1 Hz, 1H), 7.90 (d, *J* = 8.0 Hz, 1H), 7.78 – 7.75 (m, 1H), 7.33 (td, *J* = 7.8, 3.1 Hz, 2H), 7.03 (d, *J* = 7.5 Hz, 1H), 6.99 (dd, *J* = 7.4, 1.3 Hz, 1H), 3.94 (s, 3H), 3.90 (s, 2H), 3.77 – 3.73 (m, 4H), 2.72 (t, *J* = 5.9 Hz, 1H), 2.29 (s, 3H), 2.01 (d, *J* = 5.9 Hz, 6H), 0.87 (s, 18H), 0.04 (d, *J* = 1.1 Hz, 12H).

*Synthesis of N-(2,2'-dimethyl-3'-(5-((methyl (2,2,3,3,9,9,10,10-octamethyl-4,8-dioxa-3,9-disilaundecan-6-yl)amino)methyl)picolinamido)-[1,1'-biphenyl]-3-yl)-5(hydroxymethyl) picolinamide* ***(1.13_5)***

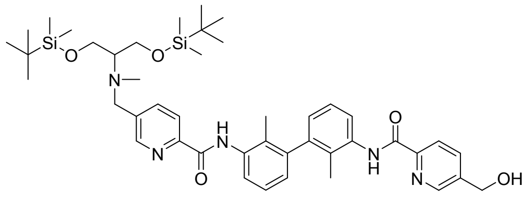

(1.13_5)

**(1.12_5)** (0.24 g, 0.29 mmol, 1 equiv.) was dissolved in THF/MeOH (10:5, 2.4 ml : 1.2 ml) and cooled to 0°C. Then solution of 4M $LiBH_4$ in THF (0.363 ml, 1.45 mmol, 5 equiv.) was added dropwise and reaction was stirred at this temperature for 15 min and then left to warm to RT. Reaction was monitored by TLC. After full consumption of SM (15 min) water was added and solvent was evaporated. To the residue DCM was added and reaction was extracted with DCM/water. The organic layer was washed with brine, dried over $Na_2SO_4$ and evaporated to give **(1.13_5)** (0.23 g) as yellowish oil. **$^1$H NMR** (600 MHz, DMSO) δ 10.34 (d, $J$ = 6.4 Hz, 2H), 8.66 (dd, $J$ = 2.1, 0.9 Hz, 1H), 8.63 (d, $J$ = 2.1 Hz, 1H), 8.18 – 8.10 (m, 2H), 7.99 (ddd, $J$ = 11.3, 7.9, 2.1 Hz, 2H), 7.91 – 7.87 (m, 2H), 7.33 (t, $J$ = 7.8 Hz, 2H), 6.99 (dt, $J$ = 7.6, 1.7 Hz, 2H), 5.51 (t, $J$ = 5.7 Hz, 1H), 4.66 (d, $J$ = 5.6 Hz, 2H), 3.90 (s, 2H), 3.79 – 3.68 (m, 4H), 2.72 (p, $J$ = 5.9 Hz, 1H), 2.29 (s, 3H), 2.02 (d, $J$ = 1.1 Hz, 6H), 0.87 (s, 18H), 0.04 (d, $J$ = 1.1 Hz, 12H).

*Synthesis of N-(2,2'-dimethyl-3'-(5-((methyl(2,2,3,3,9,9,10,10-octamethyl-4,8-dioxa-3,9-disilaundecan-6-yl)amino)methyl) picolinamido)-[1,1'-biphenyl]-3-yl)-5-formylpicolinamide **(1.14_5)***

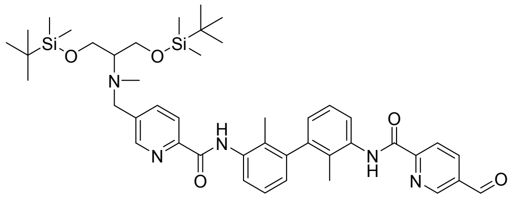

(1.14_5)

**(1.13_5)** (0.23 g, 0.29 mmol, 1 equiv.) was dissolved in DCM (6.9 ml) and cooled to 0°C. Then, Dess-Martin periodinane (0.244 g, 0.58 mmol, 2 equiv.) and $NaHCO_3$ (0.121 g, 1.44 mmol, 5 equiv.) were added and reaction was stirred for 0.5 h at 0°C. Next reaction was left warm to RT and stirred for 30 min (no SM). After that time $NaHCO_3$ was added and reaction was extracted with water. The organic layer was washed with brine, dried over $Na_2SO_4$ and evaporated. Crude was purified by flash chromatography using Hex:EtOAc (0-50%) to give **(1.14_5)** (0.174 g, 76% yield) as yellowish solid. **$^1$H NMR** (600 MHz, DMSO) δ 10.51 (s, 1H), 10.34 (s, 1H), 10.23 (s, 1H), 9.22 (dd, $J$ = 2.1, 0.8 Hz, 1H), 8.65 – 8.62 (m, 1H), 8.51 (dd, $J$ = 8.0, 2.0 Hz, 1H), 8.36 (d, $J$ = 8.1 Hz, 1H), 8.12 (d, $J$ = 8.0 Hz, 1H), 7.98 (dd, $J$ = 8.0, 2.0 Hz, 1H), 7.90 (d, $J$ = 7.9 Hz, 1H), 7.78 (d, $J$ = 8.2 Hz, 1H), 7.36 – 7.31 (m, 2H), 7.05 – 7.02 (m, 1H), 6.99 (dd, $J$ = 7.8, 1.3 Hz, 1H), 3.90 (s, 2H), 3.79 – 3.71 (m, 4H), 2.74 – 2.71 (m, 1H), 2.29 (s, 3H), 2.02 (s, 6H), 0.87 (s, 18H), 0.04 (d, $J$ = 1.1 Hz, 12H).

*Synthesis of N-(2,2'-dimethyl-3'-(5-((methyl(2,2,3,3,9,9,10,10-octamethyl-4,8-dioxa-3,9-disilaundecan-6-yl)amino)methyl)picolinamido)-[1,1'-biphenyl]-3-yl)-5-(((3-hydroxypropyl) amino)methyl)picolinamide* ***(1.15_5)***

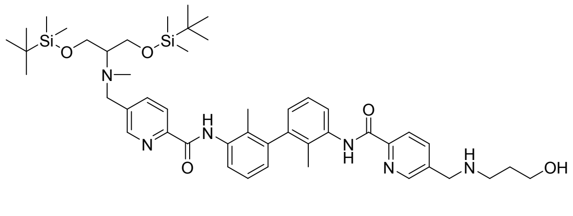


(1.15_5)

**(1.14_5)** (0.174 g, 0.22 mmol, 1 equiv.), 3-Amino-1-propanol (0.067 ml, 0.87 mmol, 4 equiv.), and few drops of AcOH were dissolved in a mixture of DCE/MeOH (20:10, 3.48 ml : 1.74 ml) and left for stirring for 2 h. Then $NaBH_3CN$ (0.11 g, 1.75 mmol, 8 equiv.) was added and reaction was left for overnight stirring. After that, water was added and reaction was extracted with EtOAc. The organic layer was washed with brine, dried over $Na_2SO_4$ and evaporated. Crude was purified by flash chromatography using DCM:MeOH (0-20%) to give **(1.15_5)** (0.016 g, 16% yield) as yellowish oil.

*Synthesis of 5-(((1,3-dihydroxypropan-2-yl)(methyl)amino) methyl)-N-(3'-(5-(((3-hydroxypropyl)amino)methyl)picolinamido)-2,2'-dimethyl-[1,1'-biphenyl]-3-yl)picolinamide* ***(5)***

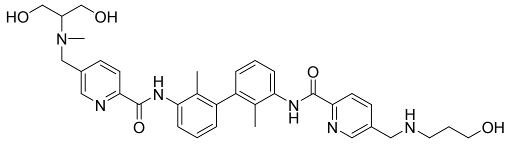


(5)

**(1.15_5)** (0.03 g, 0.04 mmol, 1 equiv.) was dissolved in DCM (0.9 ml) then 6 M HCl in i-PrOH (0.058 ml, 0.35 mmol, 10 equiv.) was added and reaction was left for stirring at RT overnight. Next, solvent was evaporated and residue was dissolved in water. Then 2 M NaOH was added and reaction was extracted with DCM. The organic layer was washed with brine, dried over $Na_2SO_4$ and evaporated. Crude was purified by pTLC using DCM:MeOH (8:2) to give **(5)** (0.03 g, 15% yield) as yellowish solid. **$^1$H NMR** (600 MHz, MeOD) δ 8.70 – 8.67 (m, 2H), 8.19 (ddd, *J* = 13.5, 8.0, 0.8 Hz, 2H), 8.03 (ddd, *J* = 17.0, 8.0, 2.1 Hz, 2H), 7.92 (ddd, *J* = 8.1, 4.3, 1.3 Hz, 2H), 7.33 (t, *J* = 7.8 Hz, 2H), 7.05 (dt, *J* = 7.6, 1.6 Hz, 2H), 3.95 (s, 2H), 3.91 (s, 2H), 3.75 (dd, *J* = 11.3, 7.1 Hz, 2H), 3.65 (dt, *J* = 12.4, 5.8 Hz, 4H), 2.86 (tt, *J* = 7.0, 5.6 Hz, 1H), 2.74 (t, *J* = 7.2 Hz, 2H), 2.34 (s, 3H), 2.10 (s, 6H), 1.77 (dq, *J* = 9.1, 6.4 Hz, 2H). **LC-MS** (DAD/ESI): $t_R$= 3.49 min, Calcd for $C_{35}H_{42}N_6O_5$ (m/z): $[M+H]^+$ 627.33; found, $[M+H]^+$ 627.46, purity: 100%.

**Compound (9)**

**5-((4-hydroxypiperidin-1-yl)methyl)-N-(3'-(5-(((3-hydroxypropyl)amino)methyl) picolinamido)-2,2'-dimethyl-[1,1'-biphenyl]-3-yl)picolinamide**

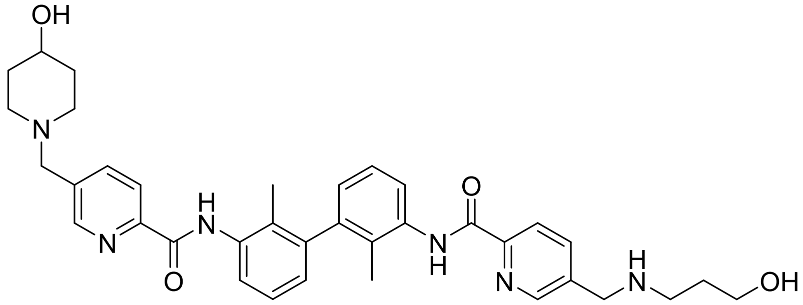

*Synthesis of tert-butyl (3'-(5-((4-hydroxypiperidin-1-yl)methyl)picolinamido)-2,2'-dimethyl-[1,1'-biphenyl]-3-yl)carbamate* ***(1.7_9)***

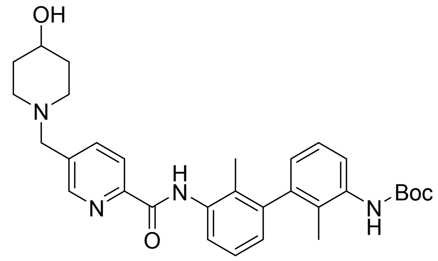

**(1.7_9)**

**(1.6_2)** (2 g, 3.92 mmol, 1 equiv.) was dissolved in ACN (40 ml), then $K_2CO_3$ (1.625 g, 11.75 mmol, 3 equiv.), KI (0.65 g, 3.92 mmol, 1 equiv.) and 4-Hydroxypiperidine (0.793 g, 7.84 mmol, 2 equiv.) were added. Mixture was left for stirring at 50$^o$C overnight. Next, water was added and reaction was extracted with DCM. The organic layer was washed with brine, dried over $Na_2SO_4$ and evaporated to give **(1.7_9)** (2 g, 96% yield) as white foam. **$^1$H NMR** (600 MHz, DMSO) δ 10.32 (s, 1H), 8.63 – 8.58 (m, 2H), 8.16 – 8.09 (m, 1H), 7.96 (dd, *J* = 8.0, 2.1 Hz, 1H), 7.84 (dd, *J* = 8.1, 1.3 Hz, 1H), 7.32 (s, 1H), 7.30 (t, *J* = 7.8 Hz, 1H), 7.21 (t, *J* = 7.8 Hz, 1H), 6.94 (dd, *J* = 7.6, 1.3 Hz, 1H), 6.90 (dd, *J* = 7.6, 1.3 Hz, 1H), 4.56 (d, *J* = 4.1 Hz, 1H), 3.59 (s, 2H), 3.46 (s, 1H), 2.67 (d, *J* = 11.2 Hz, 2H), 2.13 – 2.06 (m, 2H), 1.97 (s, 3H), 1.89 (s, 3H), 1.70 (d, *J* = 12.0 Hz, 2H), 1.46 (s, 9H), 1.40 (d, *J* = 9.8 Hz, 2H).

*Synthesis of N-(3'-amino-2,2'-dimethyl-[1,1'-biphenyl]-3-yl)-5-((4-hydroxypiperidin-1-yl)methyl)picolinamide* ***(1.8_9)***

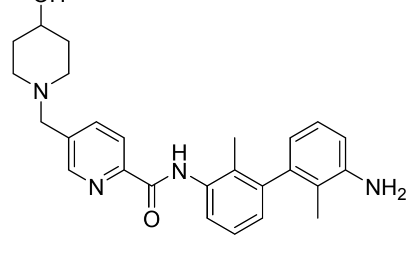

**(1.8_9)**

**(1.7_9)** (2 g, 3.77 mmol, 1 equiv.) was dissolved in DCM (60 ml) then 6 M HCl in i-PrOH (6.281 ml, 37.69 ml, 10 equiv.) was added and reaction was left for stirring at RT overnight. Then, 2 M NaOH was added and reaction was extracted with DCM and water. The organic layer was washed with brine, dried over $Na_2SO_4$ and evaporated to give **(1.8_9)** (1.59 g,

98% yield) as light orange foam. **$^1$H NMR** (600 MHz, DMSO-$d_6$) δ 10.29 (s, 1H), 8.66 – 8.56 (m, 1H), 8.14 (dd, *J* = 7.9, 0.8 Hz, 1H), 7.96 (dd, *J* = 8.0, 2.1 Hz, 1H), 7.82 (dd, *J* = 8.1, 1.3 Hz, 1H), 7.26 (t, *J* = 7.8 Hz, 1H), 6.99 – 6.91 (m, 2H), 6.65 (dd, *J* = 8.0, 1.3 Hz, 1H), 6.31 (dd, *J* = 7.4, 1.3 Hz, 1H), 4.91 (s, 2H), 4.56 (d, *J* = 4.2 Hz, 1H), 3.59 (s, 2H), 3.46 (s, 1H), 2.67 (d, *J* = 11.2 Hz, 2H), 2.13 – 2.05 (m, 1H), 1.99 (s, 3H), 1.74 (s, 3H), 1.71 (d, *J* = 12.0 Hz, 2H), 1.40 (q, *J* = 11.1, 9.5 Hz, 2H).

*Synthesis of N-(3'-amino-2,2'-dimethyl-[1,1'-biphenyl]-3-yl) -5-((4-((tert-butyldimethylsilyl )oxy)piperidin-1-yl)methyl)picolinamide* ***(1.9_9)***

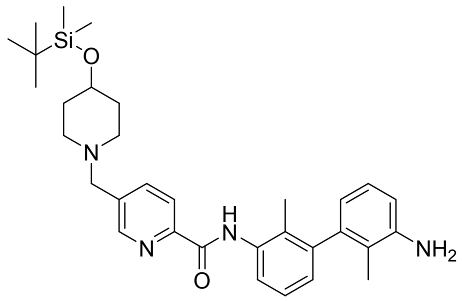

**(1.9_9)**

**(1.8_9)** (1.59 g, 3.69 mmol, 1 equiv.) was dissolved in DMF (23.85 ml) and cooled on an ice bath. Imidazole (0.629 g, 9.23 mmol, 2.5 equiv.) was then added slowly (over ˜10 minutes). A solution of TBDMS-Cl (0.696 g, 4.62 mmol, 1.25 equiv.) in DMF (3.48 ml, 5 equiv.) was added (over ˜2 minutes). The ice bath was removed and the solution was stirred at 50$^o$C overnight. Then water was added and reaction was extracted with EtOAc. The organic layer was washed with brine, dried over $Na_2SO_4$ and evaporated to give **(1.9_9)** (2 g, 99%) as yellowish oil. **$^1$H NMR** (600 MHz, DMSO) δ 10.29 (s, 1H), 8.64 – 8.62 (m, 1H), 8.13 (dd, *J* = 8.0, 0.8 Hz, 1H), 7.97 – 7.94 (m, 1H), 7.82 (dd, *J* = 8.1, 1.3 Hz, 1H), 7.26 (t, *J* = 7.7 Hz, 1H), 6.97 – 6.91 (m, 2H), 6.65 (dd, *J* = 8.0, 1.3 Hz, 1H), 6.31 (dd, *J* = 7.4, 1.3 Hz, 1H), 4.91 (s, 2H), 3.60 (s, 3H), 2.61 (p, *J* = 1.8 Hz, 0H), 2.19 (s, 2H), 1.99 (s, 3H), 1.74 (s, 3H), 1.74 – 1.69 (m, 0H), 1.45 (dtd, *J* = 12.2, 8.4, 3.4 Hz, 3H), 0.85 (d, *J* = 7.2 Hz, 9H), 0.03 (s, 6H).

*Synthesis of methyl 6-((3'-(5-((4-((tert-butyldimethylsilyl)oxy)piperidin-1-yl)methyl) picolinamido)-2,2'-dimethyl-[1,1'-biphenyl]-3-yl)carbamoyl)nicotinate* ***(1.10_9)***

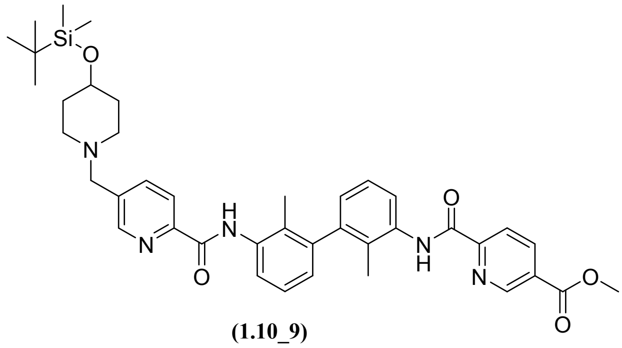

**(1.10_9)**

To a solution of 5-(Methoxycarbonyl)picolinic acid (0.665 g, 3.67 mmol, 1 equiv.) in DMF (60 ml) was added TEA (1.023 ml, 7.34 mmol, 2 equiv.). The mixture was cooled to 0$^o$C and treated with HATU (2.792 g, 7.34 mmol, 2 equiv.) and the **(1.9_9)** (2 g, 3.67 mmol, 1 equiv.).

The reaction was stirred at RT overnight. Then water was added and reaction was extracted with DCM. The organic layer was washed with brine, dried over $Na_2SO_4$ and evaporated. Crude was purified by flash chromatography using DCM:MeOH (8%) to give **(1.10_9)** (2.34 g, 99% yield) as yellowish oil.

*Synthesis of 5-((4-((tert-butyldimethylsilyl)oxy)piperidin-1-yl)methyl)-N-(3'-(5-(hydroxymethyl)picolinamido)-2,2'-dimethyl-[1,1'-biphenyl]-3-yl)picolinamide **(1.11_9)***

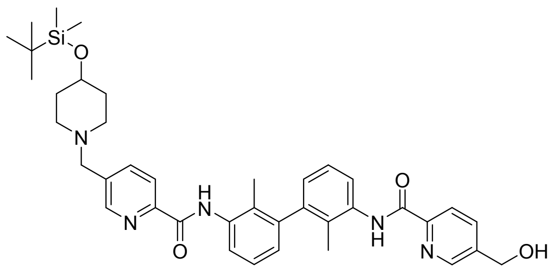

**(1.11_9)**

**(1.10_9)** (1.5 g, 2.12 mmol, 1 equiv.) was dissolved in THF/MeOH (10:5, 15 ml: 7.5 ml) and cooled to 0°C. Then solution of 4M $LiBH_4$ in THF (2.648 ml, 5 equiv.) was added dropwise and reaction was stirred at this temperature for 15 min and then left to warm to RT. Reaction was monitored by TLC. After full consumption of SM (15 min) water was added and solvent was evaporated. To the residue DCM was added and reaction was extracted with DCM/water. The organic layer was washed with brine, dried over $Na_2SO_4$ and evaporated to give **(1.11_9)** (1.73 g) as yellowish oil. **$^1$H NMR** (600 MHz, DMSO) δ 10.34 (d, $J$ = 2.8 Hz, 2H), 8.65 (ddd, $J$ = 20.2, 2.2, 0.8 Hz, 2H), 8.15 (td, $J$ = 8.0, 0.8 Hz, 2H), 8.01 – 7.95 (m, 2H), 7.91 – 7.86 (m, 2H), 7.32 (t, $J$ = 7.8 Hz, 2H), 6.99 (d, $J$ = 7.6 Hz, 2H), 5.51 (t, $J$ = 5.7 Hz, 1H), 4.66 (d, $J$ = 5.6 Hz, 2H), 3.63 – 3.59 (m, 2H), 2.61 (p, $J$ = 1.9 Hz, 2H), 2.19 (d, $J$ = 6.0 Hz, 2H), 2.02 (t, $J$ = 1.5 Hz, 6H), 1.71 (d, $J$ = 13.2 Hz, 2H), 1.45 (dtd, $J$ = 12.2, 8.5, 3.5 Hz, 2H), 0.85 (s, 9H), 0.03 (s, 6H).

*Synthesis of 5-((4-((tert-butyldimethylsilyl)oxy)piperidin-1-yl)methyl) -N-(3'-(5-formylpicolinamido)-2,2'-dimethyl-[1,1'-biphenyl]-3-yl) picolinamide **(1.12_9)***

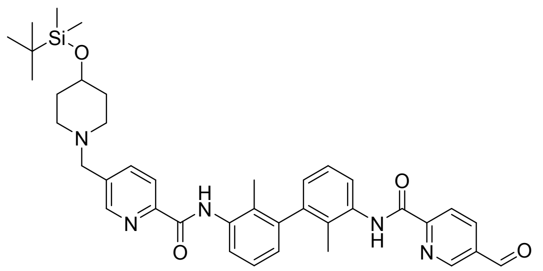

**(1.12_9)**

**(1.11_9)** (1.2 g, 1.76 mmol, 1 equiv.) was dissolved in DCM (36 ml) and cooled to 0°C. Then, Dess-Martin periodinane (2.994 g, 7.06 mmol, 4 equiv.) and $NaHCO_3$ (0.741 g, 8.82 mmol, 5 equiv.) were added and reaction was stirred for 0.5 h at 0°C. Next reaction was left warm to RT and stirred for 30 min (no SM). After that time $NaHCO_3$ was added and reaction was extracted with water. The organic layer was washed with brine, dried over $Na_2SO_4$

and evaporated. Product was macerated with hot methanol to give **(1.12_9)** (0.72 g, 60% yield) as yellowish oil.

*Synthesis of 5-((4-((tert-butyldimethylsilyl)oxy)piperidin-1-yl)methyl)-N-(3'-(5-(((3-hydroxypropyl)amino)methyl)picolinamido)-2,2'-dimethyl-[1, 1'-biphenyl]-3-yl)picolinamide **(1.13_9)***

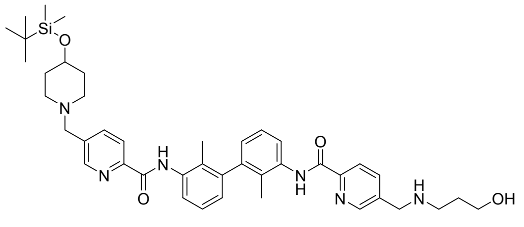


**(1.13_9)**

**(1.12_9)** (0.24 g, 0.35 mmol, 1 equiv.), 3-Amino-1-propanol (0.108 ml, 1.42 mmol, 4 equiv.), and few drops of AcOH were dissolved in a mixture of DCE/MeOH (20:10, 4.8 ml : 2.4 ml) and left for stirring for 2 h. Then sodium triacetoxyborohydride (0.6 g, 2.83 mmol, 8 equiv.) was added and reaction was left for overweekend stirring. After that, water was added and reaction was extracted with EtOAc. The organic layer was washed with brine, dried over $Na_2SO_4$ and evaporated. Crude was purified by flash chromatography using DCM:MeOH (0-20%) to give **(1.13_9)** (0.033 g, 13% yield) as yellowish oil.

*Synthesis of 5-((4-hydroxypiperidin-1-yl)methyl)-N-(3'-(5-(((3-hydroxypropyl)amino)methyl) picolinamido)-2,2'-dimethyl-[1,1'-biphenyl]-3-yl) picolinamide **(9)***

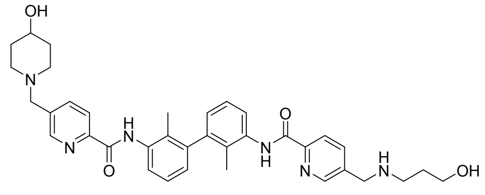


**(9)**

**(1.13_9)** (0.033 g, 0.04 mmol, 1 equiv.) was dissolved in DCM (0.99 ml) then 4 M HCl in dioxane (0.112 ml, 10 equiv.) was added and reaction was left for stirring at RT overnight. Next, solvent was evaporated and residue was dissolved in water. Then 2 M NaOH was added and reaction was extracted with DCM. The organic layer was washed with brine, dried over $Na_2SO_4$ and evaporated. Crude was purified by pTLC using DCM:MeOH (6:4) to give **(9)** (0.03 g, 15% yield) as yellowish solid. **$^1$H NMR** (600 MHz, MeOD) δ 8.68 (dd, $J$ = 2.1, 0.8 Hz, 1H), 8.66 (dd, $J$ = 2.1, 0.8 Hz, 1H), 8.20 (ddd, $J$ = 8.0, 4.5, 0.8 Hz, 2H), 8.04 – 7.99 (m, 2H), 7.94 – 7.90 (m, 2H), 7.33 (t, $J$ = 7.8 Hz, 2H), 7.05 (dd, $J$ = 7.6, 1.3 Hz, 2H), 3.92 (s, 2H), 3.66 – 3.63 (m, 4H), 2.83 – 2.78 (m, 0H), 2.75 (t, $J$ = 7.2 Hz, 2H), 2.23 (t, $J$ = 10.8 Hz, 2H), 2.10 (s, 6H), 1.88 – 1.84 (m, 2H), 1.78 (dq, $J$ = 8.9, 6.4 Hz, 2H), 1.58 (dtd, $J$ = 13.1, 9.6, 3.7 Hz, 2H). **$^{13}$C NMR** (151 MHz, MeOD) δ 164.33, 163.91, 151.55, 150.93, 150.90, 150.42, 143.72, 143.63, 140.46, 140.29, 137.08, 136.95, 130.26, 130.15, 128.10, 127.95, 127.11, 123.84, 123.76, 123.39,

123.06, 60.43, 60.05, 49.54, 47.36, 34.24, 30.00, 14.91. **LC-MS** (DAD/ESI): $t_R$= 3.58 min, Calcd for $C_{36}H_{42}N_6O_4$ (m/z): $[M+H]^+$ 623.33; found, $[M+H]^+$ 623.41, purity: 95%.

**Compound (10)**

**(S)-5-((4-hydroxypiperidin-1-yl)methyl)-N-(3'-(5-(((1-hydroxypropan-2-yl)amino) methyl)picolinamido)-2,2'-dimethyl-[1,1'-biphenyl]-3-yl)picolinamide**

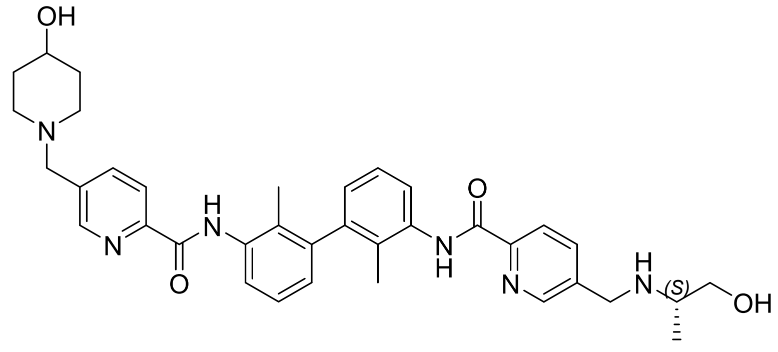

*Synthesis of (S)-5-((4-((tert-butyldimethylsilyl)oxy) piperidin-1-yl)methyl)-N-(3'-(5-(((1-hydroxypropan-2-yl)amino)methyl)picolinamido)-2,2'-dimethyl-[1,1'-biphenyl]-3-yl) picolinamide* ***(1.13_10)***

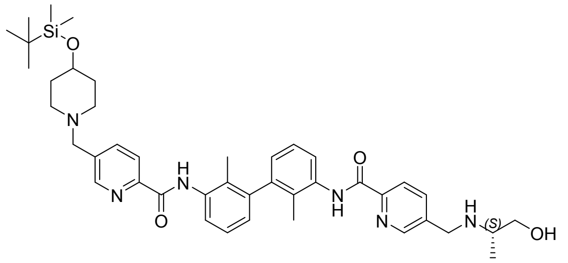

**(1.13_10)**

**(1.12_9)** (0.24 g, 0.35 mmol, 1 equiv.), (S)-2-Aminopropan-1-ol (0.11 ml, 1.42 mmol, 4 equiv.), and few drops of AcOH were dissolved in a mixture of DCE/MeOH (20:10, 4.8 ml : 2.4 ml) and left for stirring for 2 h. Then sodium triacetoxyborohydride (0.6 g, 2.83 mmol, 8 equiv.) was added and reaction was left for overweekend stirring. After that, water was added and reaction was extracted with EtOAc. The organic layer was washed with brine, dried over $Na_2SO_4$ and evaporated. Crude was purified by flash chromatography using DCM:MeOH (0-20%) to give **(1.13_10)** (0.045 g, 17% yield) as yellowish oil. **$^1$H NMR** (600 MHz, DMSO) δ 10.34 (d, $J$ = 5.0 Hz, 2H), 8.72 – 8.67 (m, 1H), 8.63 (d, $J$ = 2.0 Hz, 1H), 8.14 (d, $J$ = 8.3 Hz, 2H), 8.05 (d, $J$ = 7.6 Hz, 0H), 7.98 (dd, $J$ = 8.0, 2.1 Hz, 1H), 7.87 (d, $J$ = 8.1 Hz, 2H), 7.33 (t, $J$ = 7.9 Hz, 2H), 7.00 (d, $J$ = 7.6 Hz, 2H), 4.56 (t, $J$ = 5.6 Hz, 1H), 3.60 (s, 2H), 3.48 (q, $J$ = 5.4 Hz, 1H), 3.42 (d, $J$ = 5.4 Hz, 1H), 3.17 (d, $J$ = 5.3 Hz, 2H), 2.20 (q, $J$ = 10.7, 9.3 Hz, 3H), 1.71 (s, 2H), 1.46 (q, $J$ = 8.7 Hz, 2H), 1.07 – 0.99 (m, 0H), 0.85 (s, 9H), 0.03 (s, 6H).

*Synthesis of (S)-5-((4-hydroxypiperidin-1-yl)methyl)-N-(3'-(5-(((1-hydroxypropan-2-yl)amino) methyl)picolinamido)-2,2'-dimethyl-[1,1'-biphenyl]-3-yl)picolinamide* ***(10)***

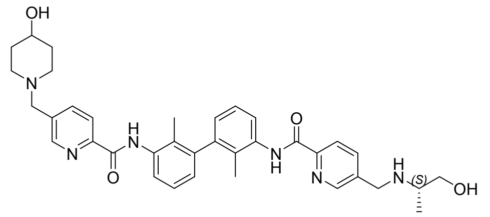

(10)

**(1.13_10)** (0.033 g, 0.04 mmol, 1 equiv.) was dissolved in DCM (0.99 ml) then 4 M HCl in dioxane (0.112 ml, 10 equiv.) was added and reaction was left for stirring at RT overnight. Next, solvent was evaporated and residue was dissolved in water. Then 2 M NaOH was added and reaction was extracted with DCM. The organic layer was washed with brine, dried over $Na_2SO_4$ and evaporated. Crude was purified by pTLC using DCM:MeOH (6:4) to give **(10)** (0.03 g, 15% yield) as yellowish solid. **$^1$H NMR** (600 MHz, MeOD) δ 8.70 (dd, *J* = 2.2, 0.8 Hz, 1H), 8.66 (dd, *J* = 2.1, 0.8 Hz, 1H), 8.20 (ddd, *J* = 8.0, 2.7, 0.8 Hz, 2H), 8.04 (dd, *J* = 8.0, 2.2 Hz, 1H), 8.00 (dd, *J* = 8.0, 2.1 Hz, 1H), 7.94 – 7.90 (m, 2H), 7.36 – 7.32 (m, 2H), 7.05 (dd, *J* = 7.6, 1.3 Hz, 2H), 4.01 (d, *J* = 13.9 Hz, 1H), 3.91 (d, *J* = 13.9 Hz, 1H), 3.66 (s, 2H), 3.64 (s, 1H), 3.54 (dd, *J* = 10.9, 4.8 Hz, 1H), 3.44 (dd, *J* = 10.9, 7.0 Hz, 1H), 2.80 (td, *J* = 6.6, 4.7 Hz, 1H), 2.24 (t, *J* = 10.8 Hz, 2H), 2.11 (d, *J* = 0.9 Hz, 6H), 1.87 (dt, *J* = 8.7, 5.8 Hz, 2H), 1.62 – 1.53 (m, 2H), 1.10 (d, *J* = 6.4 Hz, 3H). **$^{13}$C NMR** (101 MHz, METHANOL-D4) δ 164.11, 164.08, 150.41, 149.82, 149.77, 149.74, 143.45, 139.68, 138.81, 138.34, 136.85, 136.83, 129.48, 129.46, 127.66, 126.97, 123.15, 123.01, 122.87, 66.12, 60.47, 55.07, 51.99, 49.64, 49.43, 49.21, 48.79, 48.57, 48.47, 48.36, 34.67, 25.19, 16.41, 14.90, 14.88. **LC-MS** (DAD/ESI): $t_R$= 3.60 min, Calcd for $C_{36}H_{42}N_6O_4$ (m/z): $[M+H]^+$ 623.33; found, $[M+H]^+$ 623.41, purity: 100%. **HRMS** (ESI): Calcd for $C_{36}H_{42}N_6O_4$ (m/z): $[M+H]^+$ 623.3346; found, $[M+H]^+$ 623.3329, purity: 100%.

**Compound (11)**

**5-((((1R,2R)-2-hydroxycyclopentyl)amino)methyl)-N-(3'-(5-((4-hydroxypiperidin-1-yl) methyl)picolinamido)-2,2'-dimethyl-[1,1'-biphenyl]-3-yl)picolinamide**

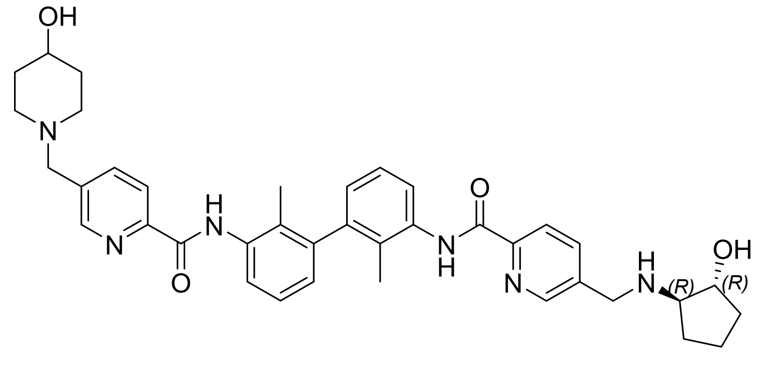

*Synthesis of 5-((4-((tert-butyldimethylsilyl)oxy)piperidin-1-yl)methyl)-N-(3'-(5-((((1R,2R)-2-hydroxycyclopentyl)amino)methyl)picolinamido)-2,2'-dimethyl-[1,1'-biphenyl]-3-yl) picolinamide* ***(1.13_11)***

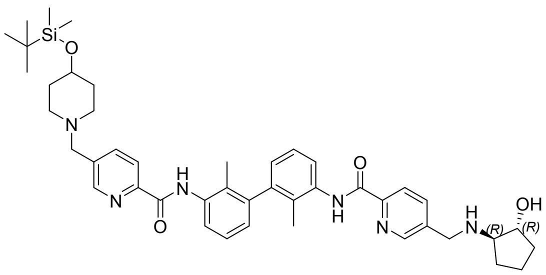

(1.13_11)

**(1.12_9)** (0.24 g, 0.35 mmol, 1 equiv.), (1R,2R)-trans-2-Aminocyclopentanol hydrochloride (0.146 g, 1.06 mmol, 3 equiv.), and few drops of AcOH were dissolved in a mixture of DCE/MeOH (20:10, 4.8 ml : 2.4 ml) and left for stirring for 2 h. Then sodium triacetoxyborohydride (0.6 g, 2.83 mmol, 8 equiv.) was added and reaction was left for overweekend stirring. After that, water was added and reaction was extracted with EtOAc. The organic layer was washed with brine, dried over $Na_2SO_4$ and evaporated. Crude was purified by flash chromatography using DCM:MeOH (0-20%) to give **(1.13_11)** (0.017 g, 6% yield) as yellowish oil. **$^1$H NMR** (600 MHz, DMSO) δ 10.34 (s, 2H), 8.68 (s, 1H), 8.63 (d, *J* = 2.0 Hz, 1H), 8.14 (dd, *J* = 7.9, 5.6 Hz, 2H), 8.03 (s, 0H), 7.97 (dd, *J* = 8.0, 2.1 Hz, 1H), 7.88 (dd, *J* = 16.0, 8.1 Hz, 2H), 7.33 (t, *J* = 7.8 Hz, 2H), 6.99 (d, *J* = 7.6 Hz, 2H), 4.56 (t, *J* = 5.6 Hz, 1H), 3.86 (s, 1H), 3.80 (s, 1H), 3.72 (s, 1H), 3.60 (s, 2H), 3.50 – 3.46 (m, 2H), 3.42 – 3.41 (m, 2H), 2.19 (dt, *J* = 17.2, 7.6 Hz, 3H), 2.02 (d, *J* = 2.7 Hz, 6H), 1.85 – 1.78 (m, 2H), 1.71 (s, 2H), 1.58 – 1.54 (m, 1H), 1.47 – 1.41 (m, 1H), 0.85 (s, 9H), 0.03 (s, 6H).

*Synthesis of 5-((((1R,2R)-2-hydroxycyclopentyl) amino)methyl)-N-(3'-(5-((4-hydroxypiperidin-1-yl)methyl)picolinamido)-2,2'-dimethyl-[1,1'-biphenyl]-3-yl)picolinamide* ***(11)***

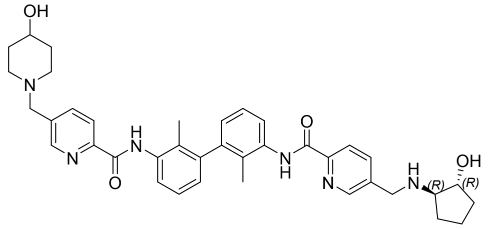

(11)

**(1.13_11)** (0.033 g, 0.04 mmol, 1 equiv.) was dissolved in DCM (0.99 ml) then 4 M HCl in dioxane (0.108 ml, 10 equiv.) was added and reaction was left for stirring at RT overnight. Next, solvent was evaporated and residue was dissolved in water. Then 2 M NaOH was added and reaction was extracted with DCM. The organic layer was washed with brine, dried over $Na_2SO_4$ and evaporated. Crude was purified by pTLC using DCM:MeOH (6:4) to give **(11)** (0.03 g, 15% yield) as yellowish solid. **$^1$H NMR** (600 MHz, MeOD) δ 8.70 (dd, *J* = 2.1, 0.8 Hz, 1H), 8.66 (dd, *J* = 2.1, 0.8 Hz, 1H), 8.20 (dt, *J* = 8.0, 1.0 Hz, 2H), 8.04 (dd, *J* = 8.0, 2.2 Hz, 1H), 8.00 (dd, *J* = 8.0, 2.1 Hz, 1H), 7.92 (dt, *J* = 8.1, 1.5 Hz, 2H), 7.36 – 7.31 (m, 2H), 7.09 – 7.03 (m, 2H), 3.98 – 3.93 (m, 3H), 3.66 (s, 2H), 3.64 (s, 2H), 2.92 – 2.88 (m, 1H), 2.80 (d, *J*

= 11.5 Hz, 2H), 2.24 (t, $J$ = 10.7 Hz, 2H), 2.12 – 2.09 (m, 6H), 2.06 – 2.01 (m, 1H), 1.97 (ddd, $J$ = 13.3, 8.0, 6.5 Hz, 1H), 1.91 – 1.83 (m, 2H), 1.76 – 1.69 (m, 2H), 1.63 – 1.54 (m, 3H), 1.42 (dt, $J$ = 13.0, 7.9 Hz, 1H).

**Compound (14)**

**(S)-5-(((1-hydroxypropan-2-yl)amino)methyl)-N-(3'-(5-(((3-hydroxypropyl)amino) methyl)picolinamido)-2,2'-dimethyl-[1,1'-biphenyl]-3-yl)picolinamide**

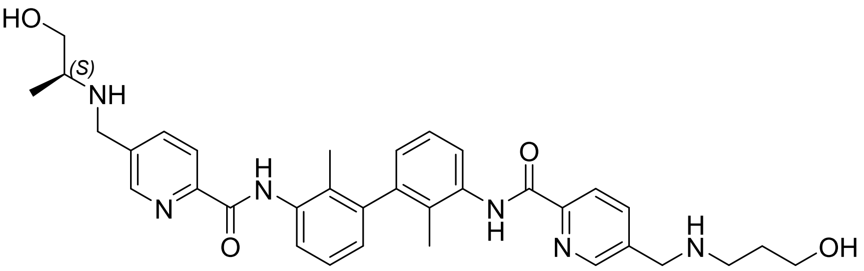

*Synthesis of N-(3'-amino-2,2'-dimethyl-[1,1'-biphenyl]-3-yl)-5-(hydroxymethyl)picolinamide* ***(1.6_14)***

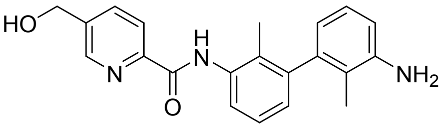

**(1.6_14)**

**(1.5_2)** (2.9 g, 6.48 mmol, 1 equiv.) was dissolved in DCM (87 ml) then 6M HCl in i-PrOH (10.8 ml, 10 equiv.) was added and reaction was left for stirring at RT overnight. Then, solvent was evaporated, residue was extracted with DCM/MeOH/water. To water phase was added KOH water solution (to obtain pH~8) and extracted with DCM/MeOH. to give **(1.6_14)** (2.06 g, 92% yield) as yellowish solid. **$^1$H NMR** (600 MHz, DMSO) δ 10.29 (s, 1H), 8.66 (dt, $J$ = 2.1, 0.8 Hz, 1H), 8.15 (dd, $J$ = 7.9, 0.8 Hz, 1H), 8.04 – 7.98 (m, 1H), 7.83 (dd, $J$ = 8.1, 1.3 Hz, 1H), 7.26 (t, $J$ = 7.8 Hz, 1H), 6.98 – 6.88 (m, 2H), 6.65 (dd, $J$ = 8.0, 1.3 Hz, 1H), 6.31 (dd, $J$ = 7.5, 1.3 Hz, 1H), 5.51 (t, $J$ = 5.7 Hz, 1H), 4.91 (s, 2H), 4.66 (d, $J$ = 5.6 Hz, 2H), 1.99 (s, 3H), 1.74 (s, 3H).

*Synthesis of N-(3'-amino-2,2'-dimethyl-[1,1'-biphenyl]-3-yl)-5-(((tert-butyldimethylsilyl) oxy)methyl)picolinamide* ***(1.7_14)***

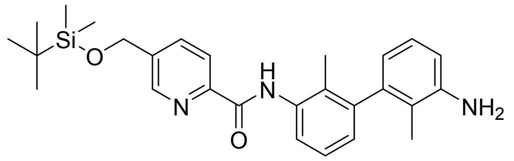

(1.7_14)

**(1.6_14)** (2.06 g, 5.93 mmol, 1 equiv.) was dissolved in DMF (30.9 ml) and cooled on an ice bath. Imidazole (1.009 g, 14.82 mmol, 2.5 equiv.) was then added slowly (over ˜10 minutes). A solution of TBDMS-Cl (1.117 g, 7.41 mmol, 1.25 equiv.) in DMF (5.59 ml) was added (over ˜2 minutes). The ice bath was removed and the solution was stirred at 50°C overnight. Then water was added and reaction was extracted with EtOAc. The organic layer was washed with brine, dried over $Na_2SO_4$ and evaporated. Crude was purified by flash chromatography using Hex:EtOAc (0-50%) to give **(1.7_14)** (2.1 g, 77% yield) as white solid. **$^1$H NMR** (600 MHz, DMSO) δ 10.29 (s, 1H), 8.66 (dd, *J* = 2.1, 0.9 Hz, 1H), 8.17 (dd, *J* = 8.0, 0.8 Hz, 1H), 7.98 (ddt, *J* = 8.0, 1.9, 0.8 Hz, 1H), 7.82 (dd, *J* = 8.1, 1.3 Hz, 1H), 7.26 (t, *J* = 7.8 Hz, 1H), 6.97 – 6.91 (m, 2H), 6.65 (dd, *J* = 8.0, 1.3 Hz, 1H), 6.31 (dd, *J* = 7.4, 1.3 Hz, 1H), 4.91 (s, 2H), 4.88 (s, 2H), 1.99 (d, *J* = 1.7 Hz, 3H), 1.74 (s, 3H), 0.92 (s, 9H), 0.12 (s, 6H).

*Synthesis of 5-bromo-N-(3'-(5-(((tert-butyldimethylsilyl) oxy)methyl)picolinamido)-2,2'-dimethyl-[1,1'-biphenyl]-3-yl)picolinamide* ***(1.8_14)***

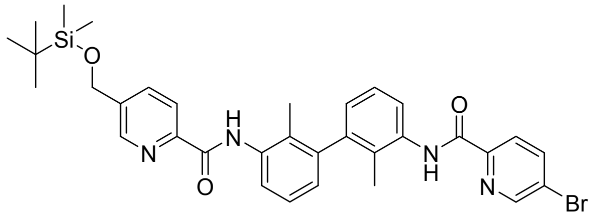

(1.8_14)

To a solution of 5-bromopicolinic acid (0.936 g, 4.64 mmol, 1 equiv.) in DMF (64.2 ml) was added TEA (1.292 ml, 9.27 mmol, 2 equiv.). The mixture was cooled to 0°C and treated with HATU (3.525 g, 9.27 mmol, 2 equiv.) and the **(1.7_14)** (2.14 g, 4.64 mmol, 1 equiv.). The reaction was stirred at RT overnight. Then water was added and reaction was extracted with DCM. The organic layer was washed with brine, dried over $Na_2SO_4$ and evaporated. Crude was purified by flash chromatography using Hex:DCM:MeOH (8%) to give **(1.8_14)** (1.8 g, 60% yield) as yellowish solid. **$^1$H NMR** (600 MHz, DMSO) δ 10.33 (d, *J* = 8.6 Hz, 2H), 8.88 (d, *J* = 2.3 Hz, 1H), 8.72 – 8.65 (m, 1H), 8.34 (dd, *J* = 8.4, 2.3 Hz, 1H), 8.17 (d, *J* = 8.0 Hz, 1H), 8.10 (d, *J* = 8.3 Hz, 1H), 7.98 (dd, *J* = 8.0, 2.1 Hz, 1H), 7.87 (d, *J* = 8.2 Hz, 1H), 7.77 (d, *J* = 8.0 Hz, 1H), 7.32 (t, *J* = 7.8 Hz, 2H), 7.00 (ddd, *J* = 14.2, 7.6, 1.3 Hz, 2H), 4.89 (s, 2H), 2.01 (d, *J* = 11.5 Hz, 6H), 0.92 (s, 9H), 0.12 (s, 6H).

*Synthesis of 5-(((tert-butyldimethylsilyl)oxy)methyl)-N-(2,2'-dimethyl-3'-(5-vinylpicolinamido)-[1,1'-biphenyl]-3-yl)picolinamide* ***(1.9_14)***

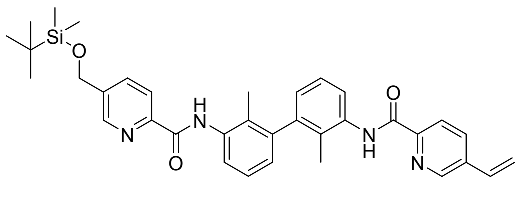

**(1.9_14)**

Potassium trifluoro(vinyl)borate (0.851 g, 6.35 mmol, 2.05 equiv.) was added to **(1.8_14)** (2 g, 3.1 mmol, 1 equiv.) and potassium carbonate (1.712 g, 12.39 mmol, 4 equiv.) in dioxane/water (50:10, 100 ml : 20 ml). Through the mixture argon was flush and all was bubbled for 10 min. Then Pd(dppf)Cl2*DCM (0.126 g, 0.15 mmol, 0.05 equiv.) was added and reaction was left for overnight stirring at 90°C. After that, reaction was cooled to RT, water was added and reaction was extracted with EtOAc. The organic layer was washed with brine, dried over $Na_2SO_4$ and evaporated. Crude was purified by flash chromatography using Hex:EtOAc (0-50%) to give **(1.9_14)** (1.19 g, 65% yield) as yellowish solid.

*Synthesis of N-(2,2'-dimethyl-3'-(5-vinylpicolinamido)-[1,1'-biphenyl]-3-yl)-5-(hydroxymethyl) picolinamide* ***(1.10_14)***

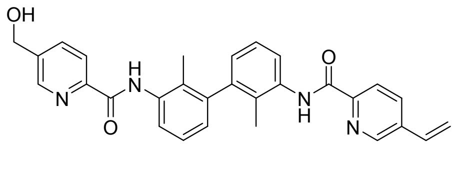

**(1.10_14)**

**(1.9_14)** (1.19 g, 2.01 mmol, 1 equiv.) was dissolved in DCM (35.7 ml) then 6M HCl in i-PrOH (2.676 ml, 10 equiv.) was added and reaction was left for stirring at RT overnight. Then, solvent was evaporated, residue was extracted with DCM/MeOH/water. To water phase was added KOH water solution (to obtain pH~8) and extracted with DCM/MeOH to give **(1.10_14)** (0.96 g of crude) as yellowish solid. $^1$**H NMR** (600 MHz, DMSO) δ 10.33 (d, *J* = 10.8 Hz, 2H), 8.83 (d, *J* = 2.1 Hz, 1H), 8.66 (dd, *J* = 2.1, 0.9 Hz, 1H), 8.21 (dd, *J* = 8.2, 2.2 Hz, 1H), 8.18 – 8.13 (m, 2H), 8.03 – 7.98 (m, 1H), 7.89 (td, *J* = 8.3, 1.3 Hz, 2H), 7.33 (td, *J* = 7.8, 1.7 Hz, 2H), 6.99 (dt, *J* = 7.5, 1.5 Hz, 2H), 6.90 (dd, *J* = 17.7, 11.1 Hz, 1H), 6.17 (d, *J* = 17.9 Hz, 1H), 5.56 (d, *J* = 11.2 Hz, 1H), 5.51 (t, *J* = 5.7 Hz, 1H), 4.66 (d, *J* = 5.7 Hz, 2H), 2.02 (d, *J* = 2.3 Hz, 6H).

*Synthesis of N-(2,2'-dimethyl-3'-(5-vinylpicolinamido)-[1,1'-biphenyl]-3-yl)-5-formyl picolinamide* ***(1.11_14)***

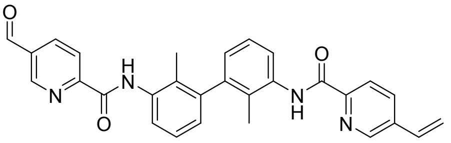

(1.11_14)

**(1.10_14)** (0.96 g, 2.01 mmol, 1 equiv.) was dissolved in DCM (28.8 ml) and cooled to 0°C. Then, Dess-Martin periodinane (1.702 g, 4.01 mmol, 2 equiv.) and $NaHCO_3$ (0.843 g, 10.03 mmol, 5 equiv.) were added and reaction wa stirred for 0.5 h at 0°C. Next reaction was left warm to RT and stirred for 30 min (no SM). After that time $NaHCO_3$ was added and reaction was extracted with water. The organic layer was washed with brine, dried over $Na_2SO_4$ and evaporated. Product was macerated with hot methanol to give **(1.11_14)** (0.96 g, 100% yield) as yellowish solid. **$^1$H NMR** (600 MHz, DMSO) δ 10.51 (s, 1H), 10.33 (s, 1H), 10.23 (s, 1H), 9.22 (dd, *J* = 2.0, 0.8 Hz, 1H), 8.83 (s, 1H), 8.51 (dd, *J* = 8.0, 2.0 Hz, 1H), 8.36 (d, *J* = 8.0 Hz, 1H), 8.21 (dd, *J* = 8.2, 2.1 Hz, 1H), 8.15 (d, *J* = 8.2 Hz, 1H), 7.89 (dd, *J* = 8.0, 1.3 Hz, 1H), 7.78 (dd, *J* = 8.1, 1.3 Hz, 1H), 7.34 (td, *J* = 7.8, 4.5 Hz, 2H), 7.04 – 6.98 (m, 2H), 6.90 (dd, *J* = 17.7, 11.1 Hz, 1H), 6.17 (d, *J* = 17.8 Hz, 1H), 5.56 (d, *J* = 11.1 Hz, 1H), 2.02 (d, *J* = 4.6 Hz, 7H).

*Synthesis of (S)-N-(2,2'-dimethyl-3'-(5-vinylpicolinamido) -[1,1'-biphenyl]-3-yl)-5-(((1-hydroxypropan-2-yl)amino)methyl)picolinamide* ***(1.12_14)***

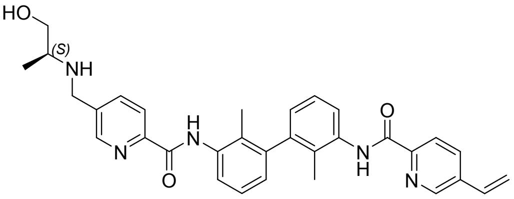

(1.12_14)

**(1.11_14)** (0.96 g, 2.01 mmol, 1 equiv.), (S)-(+)-2-Amino-1-propanol (0.462 g, 6.04 mmol, 3 equiv.), and few drops of AcOH were dissolved in a mixture of DCE/MeOH (20:10, 19.2 ml : 9.6 ml) and left for stirring for 2 h. Then sodium triacetoxyborohydride (2.135 g, 10.07 mmol, 5 equiv.) was added and reaction was left for overnight stirring. After that, water was added and reaction was extracted with EtOAc. The organic layer was washed with brine, dried over $Na_2SO_4$ and evaporated. Crude was purified by flash chromatography using DCM:MeOH (0-30%) to give **(1.12_14)** (0.46 g, 45% yield) as yellowish foam. **$^1$H NMR** (600 MHz, DMSO) δ 10.33 (d, *J* = 7.4 Hz, 2H), 8.83 (d, *J* = 2.1 Hz, 1H), 8.69 (d, *J* = 2.0 Hz, 1H), 8.20 (dd, *J* = 8.2, 2.1 Hz, 1H), 8.17 – 8.12 (m, 2H), 8.03 (dd, *J* = 8.0, 2.1 Hz, 1H), 7.93 – 7.88 (m, 2H), 7.33 (td, *J* = 7.8, 1.5 Hz, 2H), 7.03 – 6.96 (m, 2H), 6.90 (dd, *J* = 17.7, 11.1 Hz, 1H), 6.17 (d, *J* = 17.8 Hz, 1H), 5.56 (d, *J* = 11.1 Hz, 1H), 4.57 (s, 1H), 3.92 (d, *J* = 14.6 Hz, 1H), 3.86 (d, *J* = 14.5 Hz, 1H), 3.32 – 3.29 (m, 2H), 3.17 (d, *J* = 3.6 Hz, 1H), 2.02 (d, *J* = 1.8 Hz, 6H), 0.96 (d, *J* = 6.3 Hz, 3H).

*Synthesis of (S)-5-(((1-((tert-butyldimethylsilyl)oxy) propan-2-yl)amino)methyl)-N-(2,2'-dimethyl-3'-(5-vinylpicolinamido)-[1,1'-biphenyl]-3-yl)picolinamide **(1.13_14)***

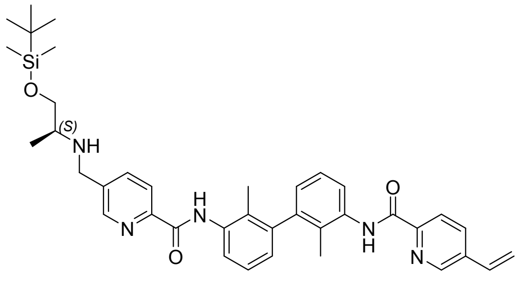

**(1.13_14)**

**(1.12_14)** (0.56 g, 1.05 mmol, 1 equiv.) was dissolved in DMF (8.4 ml) and cooled on an ice bath. Imidazole (0.178 g, 2.61 mmol, 2.5 equiv.) was then added slowly (over ~10 minutes). A solution of TBDMS-Cl (0.197 g, 1.31 mmol, 1.25 equiv.) in DMF (0.98 ml) was added (over ~2 minutes). The ice bath was removed and the solution was stirred at $50^{o}$C overnight. Then water was added and reaction was extracted with EtOAc. The organic layer was washed with brine, dried over $Na_2SO_4$ and evaporated. Crude was purified by flash chromatography using Hex:EtOAc (0-50%) to give **(1.13_14)** (0.48 g, 71% yield) as yellowish oil. **$^1$H NMR** (600 MHz, DMSO) δ 10.33 (d, *J* = 5.3 Hz, 2H), 8.83 (d, *J* = 2.1 Hz, 1H), 8.67 (d, *J* = 2.1 Hz, 1H), 8.20 (dd, *J* = 8.2, 2.2 Hz, 1H), 8.17 – 8.11 (m, 2H), 8.01 (dd, *J* = 8.0, 2.1 Hz, 1H), 7.89 (dt, *J* = 8.0, 1.6 Hz, 2H), 7.33 (td, *J* = 7.8, 1.6 Hz, 2H), 6.99 (ddd, *J* = 7.6, 3.0, 1.3 Hz, 2H), 6.90 (dd, *J* = 17.7, 11.1 Hz, 1H), 6.17 (d, *J* = 17.8 Hz, 1H), 5.56 (d, *J* = 11.3 Hz, 1H), 3.92 (d, *J* = 14.7 Hz, 1H), 3.85 (d, *J* = 14.7 Hz, 1H), 3.51 – 3.49 (m, 1H), 3.40 (dd, *J* = 9.9, 5.9 Hz, 1H), 2.66 – 2.59 (m, 1H), 2.02 (d, *J* = 3.3 Hz, 6H), 0.98 (d, *J* = 6.3 Hz, 3H), 0.85 (s, 9H), 0.02 (s, 6H).

*Synthesis of (S)-5-(((1-((tert-butyldimethylsilyl)oxy) propan-2-yl)amino)methyl)-N-(3'-(5-formylpicolinamido)-2,2'-dimethyl-[1,1'-biphenyl]-3-yl)picolinamide **(1.14_14)***

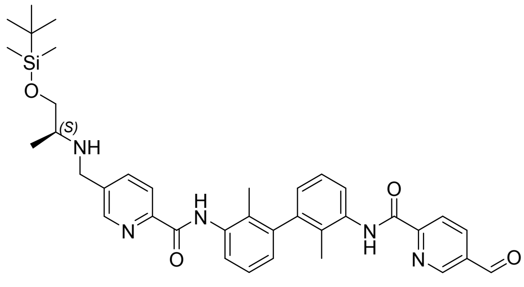

**(1.14_14)**

To **(1.13_14)** (0.48 g, 0.74 mmol, 1 equiv.) in mixture of 1,4-dioxane/water (50:10, 24 ml : 3.16 ml) was added potassium osmate (0.005 g, 0.01 mmol, 0.01 equiv.) and sodium periodate (0.316 g, 1.48 mmol, 2 equiv.). The reaction was stirred at RT for 4 h (no SM). Next, water was added and reaction was extracted with DCM. The organic layer was washed with brine, dried over $Na_2SO_4$ and evaporated. Crude was purified by flash chromatography using Hex:EtOAc (0-100%) to give **(1.14_14)** (0.29 g, 60% yield) as yellowish oil. **$^1$H NMR** (600 MHz, DMSO) δ 10.51 (s, 1H), 10.34 (s, 1H), 10.23 (s, 1H), 9.22 (dd, *J* = 2.1, 0.9 Hz, 1H), 8.70 – 8.65 (m, 1H), 8.55 – 8.50 (m, 1H), 8.36 (d, *J* = 8.0 Hz, 1H), 8.16 – 8.11

(m, 1H), 8.01 (dd, $J$ = 8.0, 2.1 Hz, 1H), 7.92 – 7.88 (m, 1H), 7.78 (dd, $J$ = 7.8, 1.2 Hz, 1H), 7.33 (q, $J$ = 7.3 Hz, 2H), 7.06 – 6.97 (m, 2H), 3.92 (d, $J$ = 14.9 Hz, 1H), 3.85 (d, $J$ = 14.8 Hz, 1H), 3.50 (dd, $J$ = 9.9, 6.0 Hz, 1H), 3.43 – 3.39 (m, 1H), 2.17 (d, $J$ = 8.6 Hz, 1H), 2.02 (d, $J$ = 1.4 Hz, 6H), 0.98 (d, $J$ = 6.3 Hz, 3H), 0.85 (s, 9H), 0.02 (s, 6H).

*Synthesis of (S)-5-(((1-((tert-butyldimethylsilyl)oxy) propan-2-yl)amino)methyl)-N-(3'-(5-(((3-hydroxypropyl)amino)methyl)picolinamido)-2,2'-dimethyl-[1,1'-biphenyl]-3-yl)picolinamide* ***(1.15_14)***

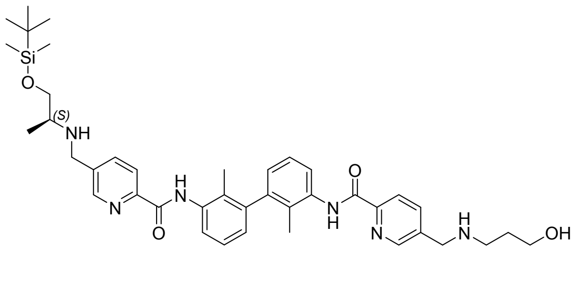

**(1.15_14)**

**(1.14_14)** (0.29 g, 0.44 mmol, 1 equiv.), 3-Amino-1-propanol (0.102 ml, 1.33 mmol, 3 equiv.), and few drops of AcOH were dissolved in a mixture of DCE/MeOH (20:10, 5.8 ml : 2.9 ml) and left for stirring for 2 h. Then sodium triacetoxyborohydride (0.566 g, 2.67 mmol, 6 equiv.) was added and reaction was left for overnight stirring. After that, water was added and reaction was extracted with EtOAc. The organic layer was washed with brine, dried over $Na_2SO_4$ and evaporated. Crude was purified by flash chromatography using DCM:MeOH (0-30%) to give **(1.15_14)** (0.53 g, 54% yield) as yellowish oil. **$^1$H NMR** (600 MHz, DMSO) δ 10.33 (d, $J$ = 1.4 Hz, 2H), 8.67 (dd, $J$ = 5.8, 2.1 Hz, 2H), 8.13 (dd, $J$ = 8.0, 3.5 Hz, 2H), 8.01 (ddd, $J$ = 8.2, 6.3, 2.1 Hz, 2H), 7.89 (d, $J$ = 7.9 Hz, 2H), 7.32 (t, $J$ = 7.8 Hz, 2H), 6.99 (dd, $J$ = 7.6, 1.3 Hz, 2H), 3.92 (d, $J$ = 14.5 Hz, 1H), 3.85 (d, $J$ = 14.7 Hz, 1H), 3.81 (s, 2H), 3.52 – 3.49 (m, 1H), 3.46 (t, $J$ = 6.3 Hz, 2H), 3.40 (dd, $J$ = 9.8, 5.8 Hz, 1H), 2.65 – 2.60 (m, 2H), 2.02 (d, $J$ = 1.5 Hz, 7H), 1.58 (p, $J$ = 6.6 Hz, 2H), 0.98 (d, $J$ = 6.3 Hz, 3H), 0.85 (s, 10H), 0.02 (s, 6H).

*Synthesis of (S)-5-(((1-hydroxypropan-2-yl)amino)methyl)-N-(3'-(5-(((3-hydroxypropyl) amino)methyl)picolinamido)-2,2'-dimethyl-[1,1'-biphenyl]-3-yl)picolinamide* ***(14)***

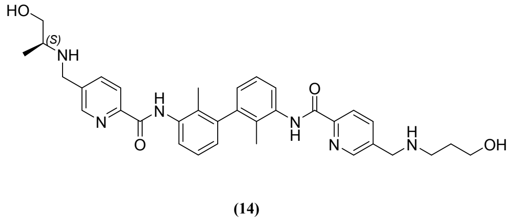

**(14)**

**(1.15_14)** (0.17 g, 0.24 mmol, 1 equiv.) was dissolved in DCM (5.1 ml) then 6M HCl in i-PrOH (0.398 ml, 10 equiv.) was added and reaction was left for stirring at RT overnight. Then, solvent was evaporated, residue was extracted with DCM/MeOH/water. To water phase was added KOH water solution (to obtain pH~8) and extracted with DCM/MeOH.

The organic layer was washed with brine, dried over $Na_2SO_4$ and evaporated. Crude was purified by pTLC using DCM:MeOH (8:2) to give **(14)** (0.0187 g, 13% yield) as white solid. **$^1$H NMR** (600 MHz, MeOD) δ 8.85 – 8.82 (m, 2H), 8.28 (td, *J* = 7.9, 0.9 Hz, 2H), 8.18 (ddd, *J* = 8.1, 4.2, 2.2 Hz, 2H), 7.89 (dt, *J* = 8.1, 1.5 Hz, 2H), 7.34 (t, *J* = 7.8 Hz, 2H), 7.06 (dt, *J* = 7.6, 1.2 Hz, 2H), 4.32 (q, *J* = 7.3, 6.6 Hz, 4H), 3.81 (dd, *J* = 11.7, 4.1 Hz, 1H), 3.71 (t, *J* = 5.9 Hz, 2H), 3.61 (dd, *J* = 11.7, 6.0 Hz, 1H), 3.31 (p, *J* = 1.6 Hz, 3H), 3.16 (t, *J* = 7.3 Hz, 2H), 2.09 (s, 6H), 1.97 – 1.91 (m, 2H), 1.33 (d, *J* = 6.7 Hz, 3H). **LC-MS** (DAD/ESI): $t_R$= 3.76 min, Calcd for $C_{34}H_{40}N_6O_4$ (m/z): $[M+H]^+$ 597.32; found, $[M+H]^+$ 597.42, purity: 100%.

**Compound (12)**

**5-((((1R,2R)-2-hydroxycyclopentyl)amino)methyl)-N-(3'-(5-(((3-hydroxypropyl)amino) methyl)picolinamido)-2,2'-dimethyl-[1,1'-biphenyl]-3-yl)picolinamide**

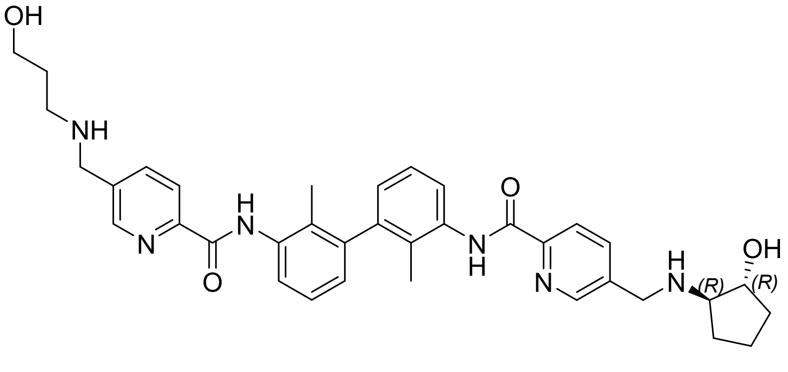


*Synthesis of N-(2,2'-dimethyl-3'-(5-vinylpicolinamido)-[1,1'-biphenyl]-3-yl)-5-(((3-hydroxypropyl)amino)methyl)picolinamide* ***(1.12_12)***

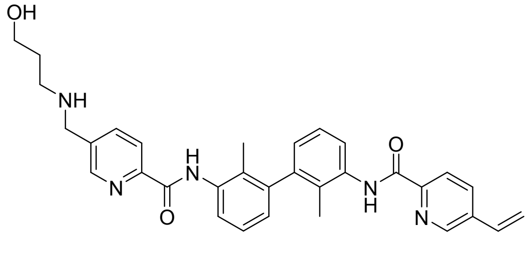


**(1.12_12)**

**(1.11_14)** (0.9 g, 1.89 mmol, 1 equiv.), 3-Amino-1-propanol (0.577 ml, 7.55 mmol, 4 equiv.), and few drops of AcOH were dissolved in a mixture of DCE/MeOH (20:10, 18 ml : 9 ml) and left for stirring for 2 h. Then sodium triacetoxyborohydride (3.202 g, 15.11 mmol, 8 equiv.) was added and reaction was left for overnight stirring. After that, water was added and reaction was extracted with EtOAc. The organic layer was washed with brine, dried over $Na_2SO_4$ and evaporated. Crude was purified by flash chromatography using DCM:MeOH (0-30%) to give **(1.12_12)** (0.46 g, 45% yield) as yellowish foam. **$^1$H NMR** (600 MHz, DMSO) δ 10.38 (s, 1H), 10.33 (s, 1H), 8.83 (d, *J* = 2.1 Hz, 1H), 8.79 (s, 1H), 8.27 – 8.19 (m, 2H), 8.15 (d, *J* = 8.2 Hz, 2H), 7.89

(dd, *J* = 8.0, 1.3 Hz, 1H), 7.83 (d, *J* = 8.0 Hz, 1H), 7.34 (dd, *J* = 7.8, 2.2 Hz, 2H), 7.00 (ddd, *J* = 10.2, 7.6, 1.3 Hz, 2H), 6.90 (dd, *J* = 17.7, 11.1 Hz, 1H), 6.22 – 6.14 (m, 1H), 5.56 (d, *J* = 11.1 Hz, 1H), 4.17 (s, 2H), 3.48 (d, *J* = 6.0 Hz, 2H), 2.89 (s, 2H), 2.02 (d, *J* = 4.8 Hz, 6H), 1.72 (s, 2H).

*Synthesis of 5-(((3-((tert-butyldimethylsilyl)oxy)propyl) amino)methyl)-N-(2,2'-dimethyl-3'-(5-vinylpicolinamido)-[1,1'-biphenyl]-3-yl)picolinamide* ***(1.13_12)***

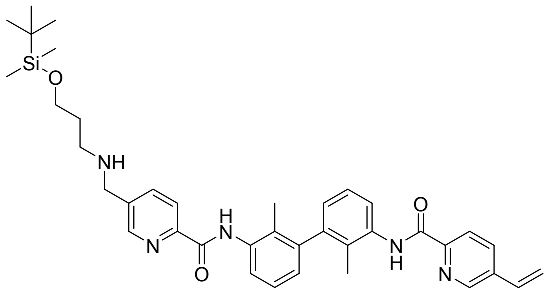


**(1.13_12)**

**(1.12_12)** (0.46 g, 0.86 mmol, 1 equiv.) was dissolved in DMF (6.9 ml) and cooled on an ice bath. Imidazole (0.146 g, 2.15 mmol, 2.5 equiv.) was then added slowly (over ˜10 minutes). A solution of TBDMS-Cl (0.162 g, 1.07 mmol, 1.25 equiv.) in DMF (0.81 ml) was added (over ˜2 minutes). The ice bath was removed and the solution was stirred at RT overnight. Then water was added and reaction was extracted with EtOAc. The organic layer was washed with brine, dried over $Na_2SO_4$ and evaporated. Crude was purified by flash chromatography using Hex:EtOAc (0-50%) to give **(1.13_12)** (0.48 g, 86% yield) as yellowish oil. **$^1$H NMR** (600 MHz, DMSO) δ 10.33 (d, *J* = 5.0 Hz, 2H), 8.83 (d, *J* = 2.1 Hz, 1H), 8.66 (dd, *J* = 2.0, 0.8 Hz, 1H), 8.20 (dd, *J* = 8.2, 2.2 Hz, 1H), 8.17 – 8.11 (m, 2H), 8.00 (dd, *J* = 8.0, 2.1 Hz, 1H), 7.89 (ddd, *J* = 8.0, 3.0, 1.3 Hz, 2H), 7.33 (t, *J* = 7.7 Hz, 2H), 7.00 – 6.97 (m, 1H), 6.90 (dd, *J* = 17.7, 11.1 Hz, 1H), 6.19 – 6.14 (m, 1H), 5.56 (d, *J* = 11.2 Hz, 1H), 3.81 (s, 2H), 3.64 (t, *J* = 6.2 Hz, 2H), 2.55 – 2.52 (m, 2H), 2.02 (d, *J* = 2.8 Hz, 6H), 1.60 (q, *J* = 6.5 Hz, 2H), 0.82 (s, 9H), 0.00 (s, 6H).

*Synthesis of 5-(((3-((tert-butyldimethylsilyl)oxy)propyl) amino)methyl) -N-(3'-(5-formylpicolinamido)-2,2'-dimethyl-[1,1'-biphenyl]-3-yl) picolinamide* ***(1.14_12)***

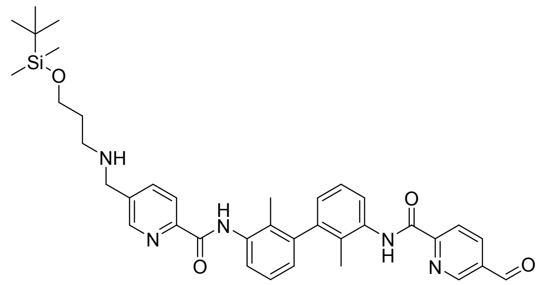

(1.14_12)

To **(1.13_12)** (0.48 g, 0.74 mmol, 1 equiv.) in mixture of 1,4-dioxane/water (50:10, 24 ml : 3.16 ml) was added potassium osmate (0.005 g, 0.01 mmol, 0.02 equiv.) and sodium periodate (0.316 g, 1.48 mmol, 2 equiv.). The reaction was stirred at RT for 2 h. Next, water was added and reaction was extracted with DCM. The organic layer was washed with brine, dried over $Na_2SO_4$ and evaporated. Crude was purified by flash chromatography using Hex:EtOAc (0-100%) to give **(1.14_12)** (0.196 g, 41% yield) as yellowish solid. **LC-MS** (DAD/ESI): $t_R$= 7.36 min, Calcd for $C_{36}H_{39}N_5O_7$ (m/z): $[M+H]^+$ 654.29; found, $[M+H]^+$ 654.12, purity: 92%.

*Synthesisis of 5-(((3-((tert-butyldimethylsilyl)oxy)propyl) amino)methyl)-N-(3'-(5-((((1R,2R)-2-hydroxycyclopentyl)amino)methyl)picolinamido)-2,2'-dimethyl-[1,1'-biphenyl]-3-yl) picolinamide **(1.15_12)***

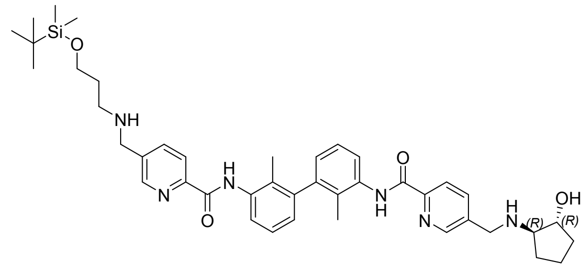

(1.15_12)

**(1.14_12)** (0.196 g, 0.3 mmol, 1 equiv.), (1R,2R)-trans-2-Aminocyclopentanol hydrochloride (0.124 g, 0.9 mmol, 3 equiv.), and few drops of AcOH were dissolved in a mixture of DCE/MeOH (20:10, 3.92 ml : 1.96 ml) and left for stirring for 2 h. Then sodium triacetoxyborohydride (0.51 g, 2.41 mmol, 8 equiv.) was added and reaction was left for overweekend stirring. After that, water was added and reaction was extracted with EtOAc. The organic layer was washed with brine, dried over $Na_2SO_4$ and evaporated. Crude was purified by flash chromatography using DCM:MeOH (0-20%) to give compound **(1.15_12)** (0.05 g, 23% yield) as yellowish oil. **$^1$H NMR** (600 MHz, DMSO) δ 10.33 (d, *J* = 1.4 Hz, 2H), 8.67 – 8.65 (m, 2H), 8.14 – 8.11 (m, 2H), 8.00 (ddd, *J* = 11.7, 8.0, 2.1 Hz, 2H), 7.89 (td, *J* = 5.9, 3.0 Hz, 2H), 7.32 (t, *J* = 7.7 Hz, 2H), 6.99 (dd, *J* = 7.6, 1.3 Hz, 2H), 4.52 (d, *J* = 4.2 Hz, 1H), 3.84 (d, *J* = 3.9 Hz, 2H), 3.81 (s, 2H), 3.79 (d, *J* = 6.7 Hz, 2H), 3.63 (t, *J* = 6.2 Hz, 2H), 2.73 (q, *J* = 6.0 Hz, 1H), 2.02 (d, *J* = 1.8 Hz, 6H), 1.84 – 1.79 (m, 1H), 1.61 (t, *J* = 6.6 Hz, 1H), 1.56 (tdd, *J* = 8.1, 5.3, 2.3 Hz, 1H), 1.42 – 1.37 (m, 1H), 1.31 – 1.24 (m, 1H), 0.85 (t, *J* = 6.9 Hz, 1H), 0.82 (s, 9H), 0.00 (s,6H).

*Synthesis of 5-((((1R,2R)-2-hydroxycyclopentyl)amino) methyl)-N-(3'-(5-(((3-hydroxypropyl) amino)methyl)picolinamido)-2,2'-dimethyl-[1,1'-biphenyl]-3-yl)picolinamide* ***(12)***

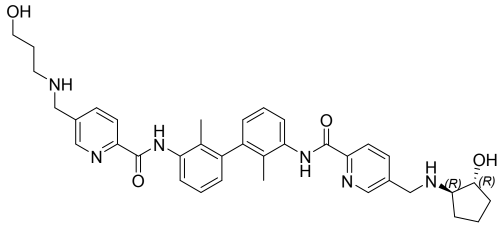

**(12)**

**(1.15_12)** (0.05 g, 0.07 mmol, 1 equiv.) was dissolved in DCM (1.5 ml) then 6M HCl in i-PrOH (0.17 ml, 10 equiv.) was added and reaction was left for stirring at RT overnight. Then, solvent was evaporated, residue was extracted with DCM/MeOH/water. To water phase was added KOH water solution (to obtain pH~8) and extracted with DCM/MeOH. to give **(12)** (0.00051 g, 1% yield) as colorless solid. $^{1}$**H NMR** (600 MHz, MeOD) δ 8.71 – 8.67 (m, 2H), 8.23 – 8.18 (m, 2H), 8.03 (ddd, *J* = 10.9, 8.0, 2.2 Hz, 2H), 7.92 (d, *J* = 8.0 Hz, 2H), 7.34 (t, *J* = 7.8 Hz, 2H), 7.08 – 7.03 (m, 2H), 3.96 (t, *J* = 3.6 Hz, 3H), 3.92 (s, 2H), 3.64 (t, *J* = 6.2 Hz, 2H), 2.94 – 2.88 (m, 1H), 2.75 (t, *J* = 7.2 Hz, 2H), 2.10 (s, 6H), 2.03 (ddd, *J* = 17.2, 7.2, 4.6 Hz, 2H), 1.99 – 1.94 (m, 1H), 1.78 (p, *J* = 6.6 Hz, 2H), 1.74 – 1.68 (m, 2H), 1.59 – 1.53 (m, 1H), 1.42 (dd, *J* = 13.1, 7.7 Hz, 1H). $^{13}$**C NMR** (151 MHz, MeOD) δ 150.08, 143.76, 139.11, 139.04, 137.18, 130.23, 127.98, 127.12, 123.79, 123.14, 123.09, 78.84, 66.87, 61.40, 51.29, 50.02, 49.42, 49.28, 49.14, 49.00, 48.86, 48.72, 48.57, 47.52, 34.11, 32.70, 31.09, 21.85, 14.89.

**Compound (16)**

**N-(2,2'-dimethyl-3'-(5-(((2-(methylsulfonamido)ethyl)amino)methyl)picolinamido)-[1,1'-biphenyl]-3-yl)-5-(((2-hydroxyethyl)amino)methyl)picolinamide**

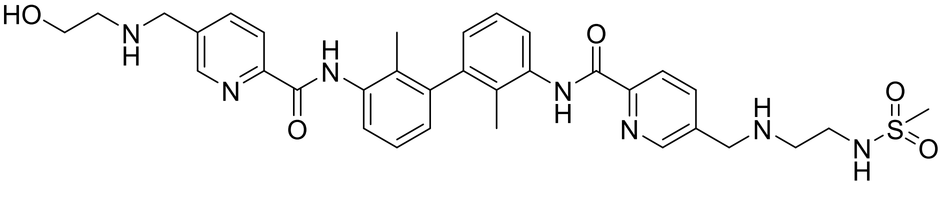

*Synthesis of tert-butyl (2-aminoethyl)carbamate* ***(1.2_16)***

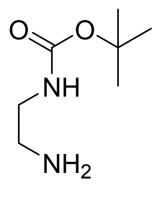

**(1.2_16)**

A solution of di-tert-butyl dicarbonate (2.179 g, 9.98 mmol, 0.2 equiv.) in DCM (30 ml) was added dropwise to a solution of ethylenediamine **(1.1_16)** (3 g, 49.92 mmol, 1 equiv.) in DCM (30 ml) at 0$^{o}$C with vigorous stirring. Stirring was continued for a further 24 h at room temperature. After concentration to an oily residue, the reaction mixture was dissolved in aqueous 2M sodium carbonate and

extracted with dichloromethane. The The organic layer was washed with 2M sodium carbonate and dried over anhydrous $MgSO_4$. The solvent was evaporated under reduced pressure to give **(1.2_16)** (0.79 g, 33% yield). **$^1$H NMR** (600 MHz, DMSO) δ 6.79 – 6.65 (m, 2H), 2.95 – 2.92 (m, 2H), 2.90 (q, *J* = 6.2 Hz, 2H), 1.37 (s, 9H).

*Synthesis of tert-butyl (2-(methylsulfonamido)ethyl) carbamate **(1.3_16)***

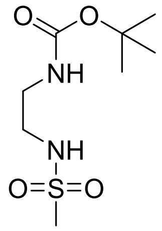


**(1.3_16)**

To a stirred solution of **(1.2_16)** (0.4 g, 2.5 mmol, 1 equiv.) and TEA (0.696 ml, 4.99 mmol, 2 equiv.) in DCM (8 ml) was added methanesulfonyl chloride (0.213 ml, 2.75 mmol, 1.1 equiv.) at 0°C and reaction was stirred at this temperature for 10 min and then left for stirring at RT for 4 h. After that time, water was added and mixture was extracted with DCM. The organic layer was washed with brine, dried over $Na_2SO_4$ and concentrated in vacuo to give **(1.3_16)** (0.47 g, crude) as yellowish solid.

*Synthesis of N-(2-aminoethyl)methanesulfonamide **(1.4_16)***

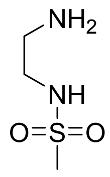


**(1.4_16)**

**(1.3_16)** (0.47 g, 1.97 mmol, 1 equiv.) was dissolved in DCM (14.10 ml) then 6M HCl in i-PrOH (3.287 ml, 10 equiv.) was added and reaction was left for stirring at RT overnight. Then, solvent was evaporated, residue was extracted with DCM/MeOH/water. Water phase was evaporated to give **(1.4_16)** (0.25 g, crude) as yellowish solid. **$^1$H NMR** (600 MHz, DMSO) δ 8.14 (s, 3H), 7.38 (t, *J* = 5.9 Hz, 1H), 3.21 (q, *J* = 6.3 Hz, 2H), 2.96 (s, 2H), 2.93 – 2.86 (m, 2H).

*Synthesis of N-(3'-amino-2,2'-dimethyl-[1,1'-biphenyl]-3-yl)-5-(hydroxymethyl)picolinamide **(1.5_16)***

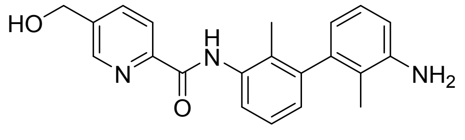


**(1.5_16)**

**(1.5_2)** (1.5 g, 3.35 mmol, 1 equiv.) was dissolved in DCM (45 ml) then 6M HCl in i-PrOH (5.586 ml, 10 equiv.) was added and reaction was left for stirring at RT overnight. Then, solvent was evaporated, residue was extracted with DCM/MeOH/water. To water phase was added KOH water solution (to obtain pH~8) and extracted with DCM/MeOH. to give **(1.5_16)** (1 g, 86% yield) as yellowish solid. **$^1$H NMR** (600 MHz, DMSO) δ 10.29 (s, 1H), 8.66 (dd, *J* = 2.1, 0.8 Hz, 1H), 8.15 (dd, *J* = 8.0,

0.8 Hz, 1H), 8.05 – 7.95 (m, 1H), 7.89 – 7.79 (m, 1H), 7.26 (t, *J* = 7.8 Hz, 1H), 7.00 – 6.89 (m, 2H), 6.65 (dd, *J* = 8.0, 1.3 Hz, 1H), 6.31 (dd, *J* = 7.4, 1.3 Hz, 1H), 5.51 (t, *J* = 5.7 Hz, 1H), 4.91 (s, 2H), 4.66 (d, *J* = 5.6 Hz, 2H), 1.99 (s, 3H), 1.74 (s, 3H).

*Synthesis of N-(3'-amino-2,2'-dimethyl-[1,1'-biphenyl]-3-yl)-5-(((tert-butyldimethylsilyl)oxy) methyl)picolinamide* ***(1.6_16)***

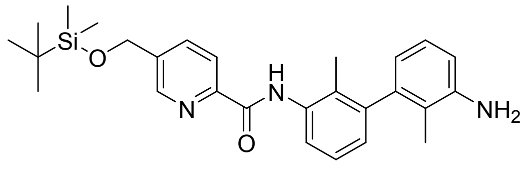


**(1.6_16)**

**(1.5_16)** (1 g, 2.88 mmol, 1 equiv.) was dissolved in DMF (15 ml) and cooled on an ice bath. Imidazole (0.49 g, 7.2 mmol, 2.5 equiv.) was then added slowly (over ˜10 minutes). A solution of TBDMS-Cl (0.542 g, 3.6 mmol, 1.25 equiv.) in DMF (2.71 ml) was added (over ˜2 minutes). The ice bath was removed and the solution was stirred at 50°C overnight. Then water was added and reaction was extracted with EtOAc. The organic layer was washed with brine, dried over $Na_2SO_4$ and evaporated. Crude was purified by flash chromatography using Hex:EtOAc (0-50%) to give **(1.6_16)** (1.54 g of crude) as colorless oil. **$^1$H NMR** (600 MHz, DMSO) δ 10.28 (s, 1H), 8.66 (dd, *J* = 2.1, 0.9 Hz, 1H), 8.17 (dd, *J* = 8.0, 0.8 Hz, 1H), 8.03 – 7.97 (m, 1H), 7.82 (dd, *J* = 8.0, 1.3 Hz, 1H), 7.26 (t, *J* = 7.8 Hz, 1H), 6.98 – 6.89 (m, 2H), 6.65 (dd, *J* = 7.9, 1.3 Hz, 1H), 6.31 (dd, *J* = 7.4, 1.3 Hz, 1H), 4.90 (s, 2H), 4.89 (s, 2H), 1.99 (s, 3H), 1.74 (s, 3H), 0.92 (s, 9H), 0.12 (s, 6H).

*Synthesis of methyl 6-((3'-(5-(((tert-butyldimethylsilyl)oxy) methyl)picolinamido)-2,2'-dimethyl-[1,1'-biphenyl]-3-yl)carbamoyl)nicotinate* ***(1.7_16)***

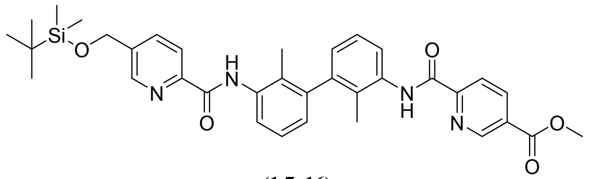


**(1.7_16)**

To a solution of 5-(Methoxycarbonyl)picolinic acid (0.471 g, 2.6 mmol, 1 equiv.) in DMF (36 ml) was added TEA (1.087 ml, 7.80 mmol, 3 equiv.). The mixture was cooled to 0°C and treated with HATU (1.087 g, 2.86 mmol, 1.1 equiv.), and the **(1.6_16)** (1.2 g, 2.6 mmol, 1 equiv.). The reaction was stirred at RT overnight. Then water was added and reaction was extracted with DCM. The organic layer was washed with brine, dried over $Na_2SO_4$ and evaporated. To crude was added MTBE and formed solid was filtered off to give **(1.7_16)** (1.19 g, 73% yield) as yellowish solid. **$^1$H NMR** (300 MHz, DMSO) δ 10.47 (s, 1H), 10.33 (s, 1H), 9.19 (dd, *J* = 2.1, 0.9 Hz, 1H), 8.67 (s, 1H), 8.56 (dd, *J* = 8.2, 2.2 Hz, 1H), 8.31 (dd, *J* = 8.2, 0.9

Hz, 1H), 8.17 (d, *J* = 8.0 Hz, 1H), 7.99 (d, *J* = 8.1 Hz, 1H), 7.87 (d, *J* = 8.0 Hz, 1H), 7.82 – 7.72 (m, 1H), 7.33 (t, *J* = 7.5 Hz, 2H), 7.01 (t, *J* = 8.5 Hz, 2H), 4.89 (s, 2H), 3.94 (d, *J* = 0.8 Hz, 3H), 2.02 (d, *J* = 3.3 Hz, 6H), 0.93 (d, *J* = 0.8 Hz, 9H), 0.12 (d, *J* = 0.8 Hz, 6H).

*Synthesis of methyl 6-((3'-(5-(hydroxymethyl)picolinamido) -2,2'-dimethyl-[1,1'-biphenyl]-3-yl)carbamoyl)nicotinate* ***(1.8_16)***

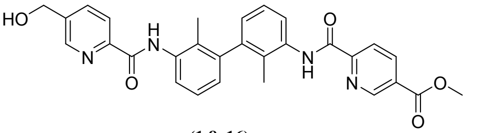


**(1.8_16)**

**(1.7_16)** (0.6 g, 0.96 mmol, 1 equiv.) was dissolved in DCM (18 ml) then 6M HCl in i-PrOH (1.6 ml, 10 equiv.) was added and reaction was left for stirring at RT overnight. Then, solvent was evaporated, residue was extracted with DCM/MeOH/water. To water phase was added KOH water solution (to obtain pH~8) and extracted with DCM/MeOH. to give **(1.8_16)** (0.43 g, 88% yield) as yellowish solid. **$^1$H NMR** (600 MHz, DMSO) δ 10.48 (s, 1H), 10.34 (s, 1H), 9.19 (dd, *J* = 2.1, 0.9 Hz, 1H), 8.66 (dd, *J* = 2.1, 0.8 Hz, 1H), 8.55 (dd, *J* = 8.1, 2.1 Hz, 1H), 8.31 (dd, *J* = 8.2, 0.9 Hz, 1H), 8.15 (dd, *J* = 8.0, 0.8 Hz, 1H), 8.00 (ddd, *J* = 8.1, 2.0, 1.0 Hz, 1H), 7.88 (dd, *J* = 8.1, 1.3 Hz, 1H), 7.76 (dd, *J* = 8.1, 1.3 Hz, 1H), 7.33 (td, *J* = 7.8, 3.9 Hz, 2H), 7.01 (ddd, *J* = 19.8, 7.6, 1.3 Hz, 2H), 5.51 (s, 1H), 4.66 (s, 2H), 3.94 (s, 3H), 2.02 (d, *J* = 6.8 Hz, 6H).

*Synthesis of methyl 6-((3'-(5-(chloromethyl)picolinamido)-2,2'-dimethyl-[1,1'-biphenyl]-3-yl)carbamoyl)nicotinate* ***(1.9_16)***

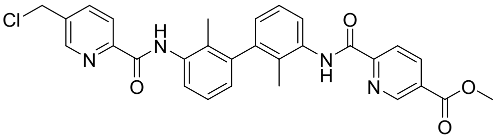


**(1.9_16)**

Solution of **(1.8_16)** (0.43 g, 0.84 mmol, 1 equiv.) in DCM (8.6 ml) was cooled to 0°C and then TEA (0.423 ml, 3.03 mmol, 3 equiv.) was added. Next, methanesulfonyl chloride (0.078 ml, 1.01 mmol, 1.2 equiv.) was added and reaction was stirred this temperature for 10 min and then left for overnight stirring at RT. After that time, solid was filtered off and filtrate was extracted with water. The organic layer was concentrated in vacuo to give **(1.9_16)** (0.53 g) as yellowish oil. **$^1$H NMR** (600 MHz, DMSO) δ 10.53 (s, 1H), 10.42 (s, 1H), 9.24 (dd, *J* = 2.1, 0.9 Hz, 1H), 8.85 (t, *J* = 2.5 Hz, 1H), 8.60 (dd, *J* = 8.1, 2.1 Hz, 1H), 8.35 (dd, *J* = 8.1, 0.9 Hz, 1H), 8.28 (d, *J* = 7.6 Hz, 1H), 8.23 – 8.17 (m, 1H), 7.88 (dd, *J* = 8.1, 1.3 Hz, 1H), 7.81 (dd, *J* = 8.1, 1.3 Hz, 1H), 7.38 (t, *J* = 7.8 Hz, 2H), 7.09 – 7.02 (m, 2H), 5.50 (s, 2H), 3.99 (s, 3H), 2.06 (d, *J* = 5.6 Hz, 6H).

*Synthesis of methyl 6-((3'-(5-(((2-hydroxyethyl)amino) methyl)picolinamido)-2,2'-dimethyl-[1,1'-biphenyl]-3-yl)carbamoyl)nicotinate* ***(1.10_16)***

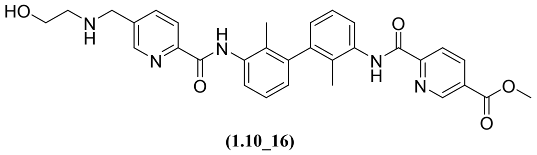

(1.10_16)

**(1.9_16)** 0.5 g, 0.95 mmol, 1 equiv.) was dissolved in ACN (10 ml), then $K_2CO_3$ (0.392 g, 2.84 mmol, 3 equiv.), KI (0.157 g, 0.95 mmol, 1 equiv.) and Ethanolamine (0.171 ml, 2.84 mmol, 3 equiv.) were added. Mixture was left for stirring at 50°C for 1 h (no SM). Next, water was added and reaction was extracted with DCM. The organic layer was washed with brine, dried over $Na_2SO_4$ and evaporated to give **(1.10_16)** (0.35 g, 67% yield) as yellowish solid. **$^1$H NMR** (600 MHz, DMSO) δ 10.48 (s, 1H), 10.33 (s, 1H), 9.19 (dd, *J* = 2.1, 0.9 Hz, 1H), 8.67 (dd, *J* = 2.1, 0.8 Hz, 1H), 8.56 (dd, *J* = 8.1, 2.1 Hz, 1H), 8.31 (dd, *J* = 8.2, 0.9 Hz, 1H), 8.17 – 8.10 (m, 1H), 8.01 (dd, *J* = 8.0, 2.1 Hz, 1H), 7.89 (dd, *J* = 8.2, 1.3 Hz, 1H), 7.76 (dd, *J* = 8.1, 1.3 Hz, 1H), 7.33 (td, *J* = 7.8, 4.0 Hz, 2H), 7.03 (d, *J* = 1.3 Hz, 0H), 4.49 (t, *J* = 5.4 Hz, 1H), 3.94 (s, 3H), 3.85 (s, 2H), 3.47 (q, *J* = 5.7 Hz, 2H), 2.57 (t, *J* = 5.8 Hz, 2H), 2.02 (d, *J* = 7.2 Hz, 6H).

*Synthesis of methyl 6-((3'-(5-(((tert-butoxycarbonyl)(2-hydroxyethyl)amino)methyl) picolinamido)-2,2'-dimethyl-[1,1'-biphenyl]-3-yl) carbamoyl)nicotinate* ***(1.11_16)***

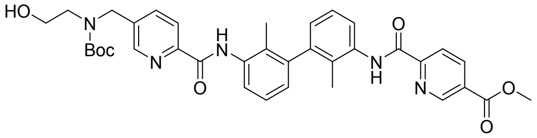

(1.11_16)

**(1.10_16)** (0.35 g, 0.63 mmol, 1 equiv.) was dissolved in ACN (7 ml). Then DIPEA (0.33 ml, 1.9 mmol, 3 equiv.) and Di-tert-butyl dicarbonate (0.179 g, 0.82 mmol, 1.3 equiv.) and were added and reaction was left for stirring at 50°C overnight. The reaction was subsequently diluted with DCM and the resulting organic solution was washed with saturated aqueous $NaHCO_3$, then brine. The organic layer was dried under sodium sulfate, concentrated to afford **(1.11_16)** (19.21 g, 83% yield) as white solid. **$^1$H NMR** (600 MHz, DMSO) δ 10.48 (s, 1H), 10.33 (s, 1H), 9.19 (dd, J = 2.1, 0.9 Hz, 1H), 8.60 (s, 1H), 8.55 (dd, J = 8.1, 2.1 Hz, 1H), 8.31 (dd, J = 8.1, 0.9 Hz, 1H), 8.16 (s, 1H), 7.91 (d, J = 8.0 Hz, 1H), 7.86 (d, J = 8.0 Hz, 1H), 7.76 (dd, J = 8.1, 1.3 Hz, 1H), 7.33 (td, J = 7.8, 4.3 Hz, 2H), 7.06 – 6.99 (m, 2H), 4.72 (t, J = 5.4 Hz, 1H), 4.57 (s, 2H), 3.94 (s, 3H), 3.50 (s, 2H), 3.29 – 3.23 (m, 2H), 2.01 (d, J = 4.8 Hz, 6H), 1.47 (s, 9H). **LC-MS** (DAD/ESI): $t_R$= 8.50 min, Calcd for $C_{36}H_{39}N_5O_7$ (m/z): $[M+H]^+$ 654.29; found, $[M+H]^+$ 654.12, purity: 92%.

*Synthesis of methyl 6-((3'-(5-(((tert-butoxycarbonyl)(2-((tert-butyldimethylsilyl)oxy) ethyl)amino)methyl)picolinamido)-2,2'-dimethyl-[1,1'-biphenyl]-3-yl)carbamoyl)nicotinate* ***(1.12_16)***

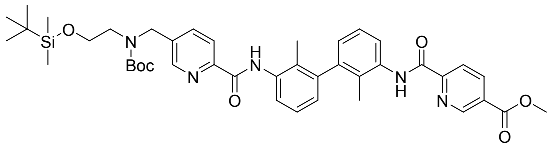

(1.12_16)

**(1.11_16)** (0.4 g, 0.61 mmol, 1 equiv.) was dissolved in DMF (6 ml) and cooled on an ice bath. Imidazole (0.104 g, 1.53 mmol, 2.5 equiv.) was then added slowly (over ˜10 minutes). A solution of TBDMS-Cl (0.115 g, 0.76 mmol, 1.25 equiv.) in DMF (0.58 ml) was added (over ˜2 minutes). The ice bath was removed and the solution was stirred at 50°C overnight. Then water was added and reaction was extracted with EtOAc. The organic layer was washed with brine, dried over $Na_2SO_4$ and evaporated. Crude was purified by flash chromatography using Hex:EtOAc (0-50%) to give **(1.12_16)** (0.51 g of crude) as yellowish oil. **$^1$H NMR** (600 MHz, DMSO) δ 10.52 (s, 1H), 10.37 (s, 1H), 9.24 (dd, *J* = 2.1, 0.9 Hz, 1H), 8.65 (s, 1H), 8.60 (dd, *J* = 8.1, 2.1 Hz, 1H), 8.35 (dd, *J* = 8.1, 0.9 Hz, 1H), 8.19 (s, 1H), 7.96 (d, *J* = 7.9 Hz, 1H), 7.91 (d, *J* = 8.1 Hz, 1H), 7.84 – 7.79 (m, 1H), 7.37 (td, *J* = 7.8, 3.4 Hz, 2H), 7.10 – 7.03 (m, 2H), 4.60 (s, 2H), 3.99 (s, 3H), 3.72 (d, *J* = 18.6 Hz, 2H), 3.43 (s, 1H), 2.06 (d, *J* = 3.1 Hz, 6H), 1.42 (d, *J* = 70.9 Hz, 9H), 0.89 (s, 9H), 0.00 (s, 6H).

*Synthesis of tert-butyl (2-((tert-butyldimethylsilyl)oxy) ethyl)((6-((3'-(5-(hydroxymethyl)picolinamido)-2,2'-dimethyl-[1,1'-biphenyl]-3-yl) carbamoyl)pyridin-3-yl)methyl)carbamate* ***(1.13_16)***

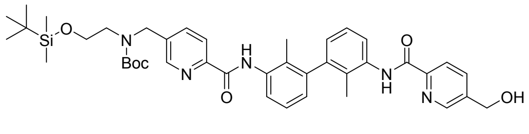

(1.13_16)

**(1.12_16)** (0.51 g, 0.66 mmol, 1 equiv.) was dissolved in THF/MeOH (10:5, 5.1 ml : 2.55 ml) and cooled to 0°C. Then solution of 4M $LiBH_4$ in THF (1.66 ml, 6.64 mmol, 10 equiv.) was added dropwise and reaction was stirred at this temperature for 15 min and then left to warm to RT. Reaction was monitored by TLC. After full consumption of SM (15 min) water was added and solvent was evaporated. To the residue DCM was added and reaction was extracted with DCM/water. The organic layer was washed with brine, dried over $Na_2SO_4$ and evaporated. Crude was recrystallized from EtOH:i-PrOH (1:2) to give **(1.13_16)** (0.43 g, crude) as yellowish foam. **$^1$H NMR** (600 MHz, DMSO) δ 10.33 (d, *J* = 4.1 Hz, 2H), 8.66 (dd, *J* = 2.1, 0.9 Hz, 1H), 8.60 (s, 1H), 8.21 – 8.12 (m, 2H), 8.00 (dd, *J* = 8.1, 2.0 Hz, 1H), 7.92 (d, *J* = 8.1 Hz, 1H), 7.89

– 7.82 (m, 2H), 7.33 (t, *J* = 7.8 Hz, 2H), 6.99 (dt, *J* = 7.6, 1.6 Hz, 2H), 5.50 (t, *J* = 5.7 Hz, 1H), 4.66 (d, *J* = 5.6 Hz, 2H), 4.56 (s, 2H), 3.68 (d, *J* = 18.6 Hz, 2H), 3.45 – 3.34 (m, 1H), 2.02 (d, *J* = 3.6 Hz, 6H), 1.46 – 1.27 (m, 9H), 0.84 (d, *J* = 3.8 Hz, 9H), 0.02 (s, 6H).

*Synthesis of tert-butyl (2-((tert-butyldimethylsilyl)oxy) ethyl)((6-((3'-(5-(chloromethyl)picolinamido)-2,2'-dimethyl-[1,1'-biphenyl]-3-yl) carbamoyl)pyridin-3-yl)methyl)carbamate* ***(1.14_16)***

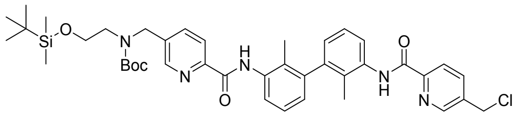

**(1.14_16)**

Solution of **(1.13_16)** (0.43 g, 0.58 mmol, 1 equiv.) in DCM (8.6 ml) was cooled to 0°C and then TEA (0.194 ml, 1.39 mmol, 2 equiv.) was added. Next, methanesulfonyl chloride (0.054 g, 0.7 mmol, 1.2 equiv.) was added and reaction was stirred this temperature for 10 min and then left for overnight stirring at RT. After that time, water was added and mixture was extracted with DCM. The organic layer was washed with brine, dried over $Na_2SO_4$ and concentrated in vacuo to give **(1.14_16)** (0.378 g) as yellowish oil. **$^1$H NMR** (600 MHz, DMSO) δ 10.37 (d, *J* = 3.5 Hz, 1H), 10.33 (s, 1H), 8.86 – 8.77 (m, 1H), 8.60 (s, 1H), 8.24 (dd, *J* = 15.1, 8.1 Hz, 1H), 8.17 (ddd, *J* = 13.3, 8.1, 2.1 Hz, 2H), 7.92 (d, *J* = 8.2 Hz, 1H), 7.84 (d, *J* = 8.2 Hz, 1H), 7.36 – 7.29 (m, 2H), 7.04 – 6.97 (m, 2H), 5.46 (d, *J* = 9.6 Hz, 2H), 4.56 (s, 2H), 3.68 (d, *J* = 18.4 Hz, 2H), 2.02 (d, *J* = 2.5 Hz, 6H), 1.43 (s, 5H), 1.31 (s, 4H), 0.84 (d, *J* = 4.0 Hz, 9H), 0.02 (s, 6H).

*Synthesis of tert-butyl (2-((tert-butyldimethylsilyl)oxy) ethyl)((6-((2,2'-dimethyl-3'-(5-(((2-(methylsulfonamido)ethyl)amino)methyl)picolinamido)-[1,1'-biphenyl]-3-yl)carbamoyl) pyridin-3-yl)methyl)carbamate* ***(1.15_16)***

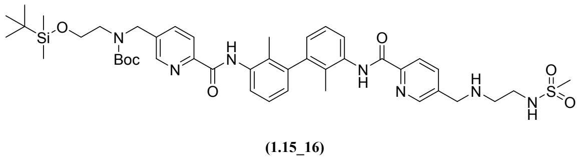

**(1.15_16)**

**(1.14_16)** (0.1 g, 0.13 mmol, 1 equiv.) was dissolved in ACN (2 ml), then $K_2CO_3$ (0.109 g, 0.79 mmol, 6 equiv.), KI (0.022 g, 0.13 mmol, 1 equiv.) and **IV** (0.055 g, 0.4 mmol, 3 equiv.) were added. Mixture was left for stirring at 50°C for 1 h (no SM). Next, water was added and reaction was extracted with DCM. The organic layer was washed with brine, dried over $Na_2SO_4$ and evaporated. Crude was purified by flash chromatography using DCM:MeOH (0-30%) to give **(1.15_16)** (0.038 g, 34% yield) as yellowish solid. **LC-MS** (DAD/ESI): $t_R$= 8.97 min, Calcd for $C_{44}H_{61}N_7O_7SSi$ (m/z): $[M-H]^-$ 858.40; found, $[M-H]^-$ 858.50, purity: 95%.

*Synthesis of N-(2,2'-dimethyl-3'-(5-(((2-(methylsulfonamido)ethyl)amino)methyl)picolinamido)-[1,1'-biphenyl]-3-yl)-5-(((2-hydroxyethyl)amino)methyl)picolinamide* ***(16)***

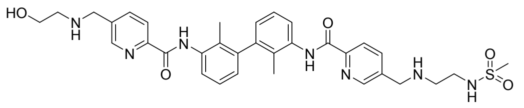

(16)

**(1.15_16)** (0.038 g, 0.04 mmol, 1 equiv.) was dissolved in DCM (1.14 ml) then 6M HCl in i-PrOH (0.074 ml, 10 equiv.) was added and reaction was left for stirring at RT overnight. Then, solvent was evaporated, residue was extracted with DCM/MeOH/water. Water phase was evaporated to give **(16)** (0.00077 g, 3% yield) as yellowish solid. **$^1$H NMR** (600 MHz, MeOD) δ 8.63 – 8.58 (m, 2H), 8.11 (ddd, *J* = 8.0, 4.3, 0.8 Hz, 2H), 7.93 (ddd, *J* = 8.0, 4.0, 2.2 Hz, 2H), 7.82 (dd, *J* = 8.0, 1.3 Hz, 2H), 7.24 (t, *J* = 7.8 Hz, 2H), 6.96 (d, *J* = 7.5 Hz, 2H), 3.87 (s, 2H), 3.83 (s, 2H), 3.63 – 3.57 (m, 2H), 3.13 (t, *J* = 6.2 Hz, 2H), 2.85 (s, 3H), 2.72 – 2.67 (m, 4H), 2.00 (s, 6H). **LC-MS** (DAD/ESI): $t_R$= 3.76 min, Calcd for $C_{33}H_{39}N_7O_5S$ (m/z): $[M+H]^+$ 644.37; found, $[M-H]^-$ 644.21, purity: 100%.

**Compound (13)**

**5-((((1R,2R)-2-hydroxycyclopentyl)amino)methyl)-N-(3'-(5-((((S)-1-hydroxypropan-2-yl)amino)methyl)picolinamido)-2,2'-dimethyl-[1,1'-biphenyl]-3-yl)picolinamide**

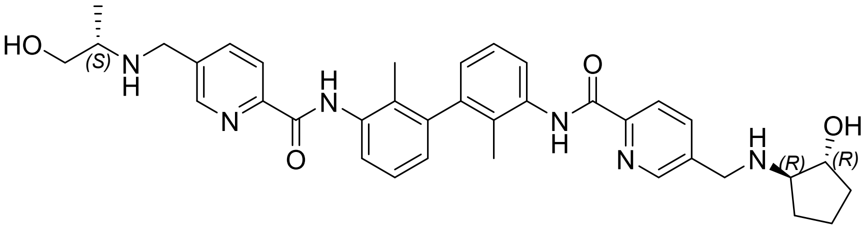

*Synthesis of methyl 6-((3'-(5-(bromomethyl)picolinamido)-2,2'-dimethyl-[1,1'-biphenyl]-3-yl)carbamoyl)nicotinate* ***(1.9_13)***

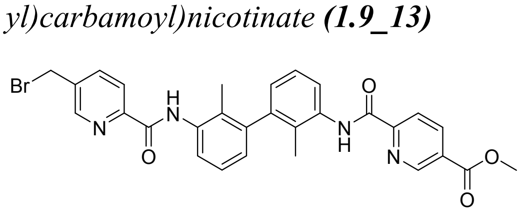

(1.9_13)

**(1.8_16)** (0.15 g, 0.29 mmol, 1 equiv.) was dissolved in DCM (1.5 ml), then NBS (0.078 g, 0.44 mmol, 1.5 equiv.) and $PPh_3$ (0.116 g, 0.44 mmol, 1.5 equiv.) were added. Reaction was left for stirring for 30 min (no SM). Then water was added and reaction was extracted with DCM. The organic layer was washed with brine, dried over $Na_2SO_4$ and evaporated. Crude was purified by flash chromatography using Hex:EtOAc (0-50%) to give **(1.9_13)** (0.17 g, 100% yield) as yellowish

solid. **$^1$H NMR** (600 MHz, DMSO) δ 10.49 (s, 1H), 10.35 (s, 1H), 9.19 (dd, *J* = 2.1, 0.9 Hz, 1H), 8.84 – 8.81 (m, 1H), 8.56 (dt, *J* = 8.1, 2.2 Hz, 1H), 8.30 (dt, *J* = 8.2, 1.1 Hz, 1H), 8.19 – 8.11 (m, 2H), 7.83 – 7.80 (m, 1H), 7.76 (s, 1H), 7.33 (td, *J* = 7.8, 3.2 Hz, 2H), 7.04 – 6.98 (m, 2H), 4.86 (s, 1H), 3.94 (s, 3H), 2.03 – 2.01 (m, 6H).

*Synthesis of methyl (S)-6-((3'-(5-(((1-hydroxypropan-2-yl) amino)methyl)picolinamido)-2,2'-dimethyl-[1,1'-biphenyl]-3-yl)carbamoyl)nicotinate* ***(1.10_13)***

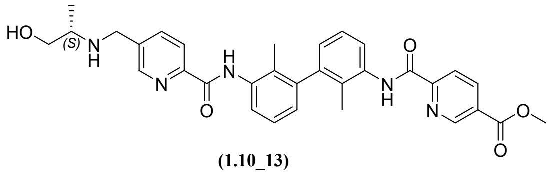

**(1.10_13)**

**(1.9_13)** (0.17 g, 0.3 mmol, 1 equiv.) was dissolved in ACN (3.4 ml), then $K_2CO_3$ (0.123 g, 0.89 mmol, 3 equiv.), KI (0.049 g, 0.3 mmol, 1 equiv.) and (S)-(+)-2-Amino-1-propanol (0.045 ml, 0.59 mmol, 2 equiv.) were added. Mixture was left for stirring at 50$^o$C for 1 h (no SM). Next, water was added and reaction was extracted with DCM. The organic layer was washed with brine, dried over $Na_2SO_4$ and evaporated to give **(1.10_13)** (0.064 g, 36% yield) as yellowish foam. **$^1$H NMR** (600 MHz, DMSO) δ 10.49 (s, 1H), 10.34 (s, 1H), 9.19 (dd, *J* = 2.1, 0.9 Hz, 1H), 8.68 (d, *J* = 2.1 Hz, 1H), 8.56 (dd, *J* = 8.2, 2.1 Hz, 1H), 8.31 (dd, *J* = 8.1, 0.9 Hz, 1H), 8.15 – 8.11 (m, 1H), 8.03 (dd, *J* = 8.0, 2.1 Hz, 1H), 7.89 (d, *J* = 8.0 Hz, 1H), 7.76 (dd, *J* = 8.1, 1.3 Hz, 1H), 7.33 (td, *J* = 7.8, 3.9 Hz, 2H), 7.01 (ddd, *J* = 21.2, 7.5, 1.3 Hz, 2H), 4.55 (dt, *J* = 15.0, 5.5 Hz, 1H), 3.94 (s, 3H), 3.87 (d, *J* = 22.9 Hz, 1H), 3.51 – 3.47 (m, 1H), 3.41 (dd, *J* = 5.7, 4.8 Hz, 1H), 3.28 (d, *J* = 5.6 Hz, 1H), 2.02 (d, *J* = 7.6 Hz, 6H), 0.95 (d, *J* = 6.3 Hz, 3H).

*Synthesis of methyl (S)-6-((3'-(5-(((1-((tert-butyldimethylsilyl)oxy)propan-2-yl)amino)methyl) picolinamido)-2,2'-dimethyl-[1,1'-biphenyl]-3-yl)carbamoyl)nicotinate* ***(1.11_13)***

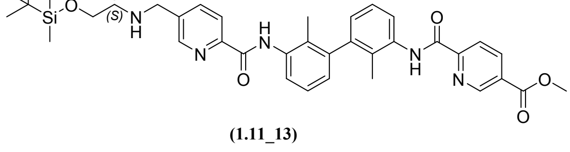

**(1.11_13)**

**(1.10_13)** (0.064 g, 0.11 mmol, 1 equiv.) was dissolved in DMF (0.96 ml) and cooled on an ice bath. Imidazole (0.019 g, 0.28 mmol, 2.5 equiv.) was then added slowly (over ˜10 minutes). A solution of TBDMS-Cl (0.021 g, 0.14 mmol, 1.5 equiv.) in DMF (0.11 ml, 5 equiv.) was added (over ˜2 minutes). The ice bath was removed and the solution was stirred at 50$^o$C overnight. Then water was added and reaction was extracted with EtOAc. The organic layer was washed with brine, dried over $Na_2SO_4$ and evaporated. Crude was purified by flash chromatography using Hex:EtOAc (0-50%) to give **(1.11_13)** (2.1 g, 77% yield) as white solid.

**$^1$H NMR** (600 MHz, DMSO) δ 10.49 (s, 1H), 10.33 (s, 1H), 9.19 (dd, *J* = 2.1, 0.9 Hz, 1H), 8.74 – 8.64 (m, 1H), 8.56 (dd, *J* = 8.1, 2.1 Hz, 1H), 8.31 (dd, *J* = 8.1, 0.9 Hz, 1H), 8.15 – 8.10 (m, 1H), 8.01 (dd, *J* = 8.0, 2.1 Hz, 1H), 7.95 (s, 1H), 7.93 – 7.87 (m, 1H), 7.76 (dd, *J* = 8.0, 1.3 Hz, 1H), 7.33 (td, *J* = 7.8, 3.8 Hz, 2H), 7.03 (dd, *J* = 7.6, 1.3 Hz, 1H), 6.99 (dd, *J* = 7.6, 1.3 Hz, 1H), 3.94 (s, 3H), 3.92 (d, *J* = 14.8 Hz, 1H), 3.85 (d, *J* = 14.7 Hz, 1H), 3.53 – 3.48 (m, 1H), 3.46 (dd, *J* = 5.6, 4.5 Hz, 1H), 3.42 – 3.38 (m, 1H), 2.01 (d, *J* = 5.9 Hz, 6H), 0.98 (d, *J* = 6.4 Hz, 3H), 0.85 (s, 8H), 0.02 (s, 5H).

*Synthesis of (S)-5-(((1-((tert-butyldimethylsilyl)oxy) propan-2-yl)amino)methyl)-N-(3'-(5-(hydroxymethyl)picolinamido)-2,2'-dimethyl-[1,1'-biphenyl]-3-yl)picolinamide* ***(1.12_13)***

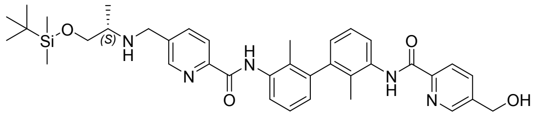


(1.12_13)

**(1.11_13)** (0.07 g, 0.1 mmol, 1 equiv.) was dissolved in THF/MeOH (10:5, 0.7 ml : 0.35 ml) and cooled to 0°C. Then solution of 4M $LiBH_4$ in THF (0.077 ml, 0.31 mmol, 3 equiv.) was added dropwise and reaction was stirred at this temperature for 15 min and then left to warm to RT. Reaction was monitored by TLC. After full consumption of SM (15 min) water was added and solvent was evaporated. To the residue DCM was added and reaction was extracted with DCM/water. The organic layer was washed with brine, dried over $Na_2SO_4$ and evaporated to give **(1.12_13)** (0.07 g of crude) as yellowish oil. **$^1$H NMR** (600 MHz, DMSO) δ 10.30 (d, *J* = 5.7 Hz, 2H), 8.68 – 8.61 (m, 1H), 8.15 – 8.07 (m, 2H), 7.97 (ddd, *J* = 10.0, 8.0, 2.1 Hz, 2H), 7.88 – 7.83 (m, 2H), 7.29 (dd, *J* = 9.1, 6.5 Hz, 2H), 6.98 – 6.94 (m, 2H), 5.48 (t, *J* = 5.7 Hz, 1H), 4.63 (d, *J* = 5.6 Hz, 2H), 3.88 (s, 0H), 3.81 (s, 0H), 3.65 – 3.63 (m, 1H), 3.48 – 3.45 (m, 1H), 3.43 (dd, *J* = 5.7, 4.5 Hz, 1H), 1.98 (d, *J* = 4.4 Hz, 6H), 0.94 (d, *J* = 6.3 Hz, 3H), 0.82 (d, *J* = 3.3 Hz, 9H), -0.00 (s, 6H).

*Synthesis of (S)-5-(((1-((tert-butyldimethylsilyl)oxy) propan-2-yl)amino)methyl)-N-(3'-(5-(chloromethyl)picolinamido)-2,2'-dimethyl-[1,1'-biphenyl]-3-yl)picolinamide* ***(1.13_13)***

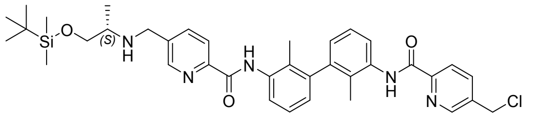


(1.13_13)

**(1.12_13)** (0.07 g, 0.11 mmol, 1 equiv.) was dissolved in DCM (1.4 ml) and cooled to 0°C. Then TEA (0.045 ml, 0.32 mmol, 3 equiv.) and methanesulfonic chloride (0.012 ml, 0.16 mmol, 1.5 equiv.) was added and reaction was stirred for 30 min (no SM). Then water was added and reaction was extracted with DCM, washed with brine, dried over $Na_2SO_4$ and evaporated to give **(1.13_13)** (0.07 g, 97% yield) as yellowish oil.

*Synthesis of 5-((((S)-1-((tert-butyldimethylsilyl)oxy) propan-2-yl)amino)methyl)-N-(3'-(2-(5-((((1R,2R)-2-hydroxycyclopentyl)amino)methyl) pyridin-2-yl)-2-oxoethyl)-2,2'-dimethyl-[1,1'-biphenyl]-3-yl)picolinamide* ***(1.14_13)***

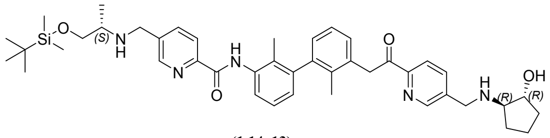

**(1.14_13)**

**(1.13_13)** (0.07 g, 0.1 mmol, 1 equiv.) was dissolved in ACN (1.4 ml), then $K_2CO_3$ (0.086 g, 0.62 mmol, 6 equiv.), KI (0.017 g, 0.1 mmol, 1 equiv.) and (1R,2R)-trans-2-Aminocyclopentanol hydrochloride (0.043 g, 0.31 mmol, 3 equiv.) were added. Mixture was left for stirring at 50$^{o}$C for 1 h (no SM). Next, water was added and reaction was extracted with DCM. The organic layer was washed with brine, dried over $Na_2SO_4$ and evaporated to give **(1.14_13)** (0.016 g, 21% yield) as yellowish foam. **$^1$H NMR** (600 MHz, MeOD) δ 8.72 – 8.67 (m, 2H), 8.20 (dt, *J* = 8.0, 0.9 Hz, 2H), 8.03 (ddd, *J* = 12.8, 8.0, 2.2 Hz, 2H), 7.92 (dd, *J* = 8.2, 1.4 Hz, 2H), 7.34 (t, *J* = 7.8 Hz, 2H), 7.05 (dd, *J* = 7.5, 1.3 Hz, 2H), 4.02 (d, *J* = 14.0 Hz, 1H), 3.96 (d, *J* = 2.3 Hz, 3H), 3.91 (d, *J* = 14.0 Hz, 1H), 3.69 – 3.66 (m, 1H), 3.64 – 3.61 (m, 1H), 3.57 – 3.55 (m, 1H), 3.52 (dd, *J* = 10.1, 7.1 Hz, 1H), 2.90 (td, *J* = 7.4, 5.0 Hz, 1H), 2.83 – 2.79 (m, 1H), 2.08 – 2.01 (m, 1H), 2.00 – 1.94 (m, 1H), 1.75 – 1.68 (m, 2H), 1.60 – 1.54 (m, 1H), 1.46 – 1.37 (m, 1H), 1.09 (d, *J* = 6.4 Hz, 2H), 0.90 (s, 9H), 0.07 (d, *J* = 4.0 Hz, 5H).

*Synthesis of 5-((((1R,2R)-2-hydroxycyclopentyl)amino) methyl)-N-(3'-(5-((((S)-1-hydroxypropan-2-yl)amino)methyl)picolinamido)-2,2'-dimethyl -[1,1'-biphenyl]-3-yl) picolinamide* ***(13)***

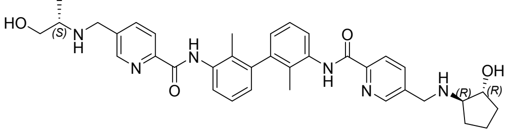

**(13)**

**(1.14_13)** (0.016 g, 0.02 mmol, 1 equiv.) was dissolved in DCM (0.48 ml) then 6M HCl in i-PrOH (0.036 ml, 0.22 mmol, 10 equiv.) was added and reaction was left for stirring at RT overnight. Then, solvent was evaporated, residue was extracted with DCM/MeOH/water. To water phase was added KOH water solution (to obtain pH~8) and extracted with DCM/MeOH. to give **(13)** (0.00161 g, 12% yield) as colorless solid. **$^1$H NMR** (600 MHz, MeOD) δ 8.71 (ddd, *J* = 6.3, 2.2, 0.8 Hz, 2H), 8.21 (ddd, *J* = 8.0, 5.5, 0.8 Hz, 2H), 8.05 (ddd, *J* = 8.0, 6.8, 2.2 Hz, 2H), 7.92 (dt, *J* = 8.1, 1.4 Hz, 2H), 7.34 (t, *J* = 7.8 Hz, 2H), 7.06 (dt, *J* = 7.6, 1.2 Hz, 2H), 4.05 (d, *J* = 13.9 Hz, 1H), 3.97 (d, *J* = 15.2 Hz, 4H), 3.58 (dd, *J* = 11.0, 4.7 Hz, 1H), 3.47 (dd, *J* = 11.0, 6.8 Hz, 1H), 2.92 (td, *J* = 7.5, 5.1 Hz, 1H), 2.88

(q, $J$ = 6.0 Hz, 1H), 2.10 (s, 6H), 2.07 – 2.03 (m, 1H), 1.98 (ddd, $J$ = 13.1, 6.5, 1.4 Hz, 1H), 1.76 – 1.68 (m, 2H), 1.61 – 1.55 (m, 1H), 1.48 – 1.41 (m, 1H), 1.13 (d, $J$ = 6.5 Hz, 3H). **$^{13}$C NMR** (101 MHz, METHANOL-D4) δ 164.54, 150.11, 150.03, 149.91, 143.77, 140.34, 140.21, 139.06, 137.21, 130.22, 127.97, 127.13, 123.79, 123.14, 123.09, 78.85, 66.88, 66.43, 55.26, 50.02, 49.64, 49.43, 49.21, 49.00, 48.79, 48.57, 48.36, 34.13, 31.11, 25.25, 21.88, 16.52, 14.90. **LC-MS** (DAD/ESI): $t_R$= 3.83 min, Calcd for $C_{36}H_{42}N_6O_4$ (m/z): $[M+H]^+$ 623.33; found, $[M+H]^+$ 623.41, purity: 95%.

**Compound (15)**

**N-(2,2'-dimethyl-3'-(5-((4-(methylsulfonyl)piperazin-1-yl)methyl)picolinamido)-[1,1'-biphenyl]-3-yl)-4-(((2-hydroxyethyl)amino)methyl)picolinamide**

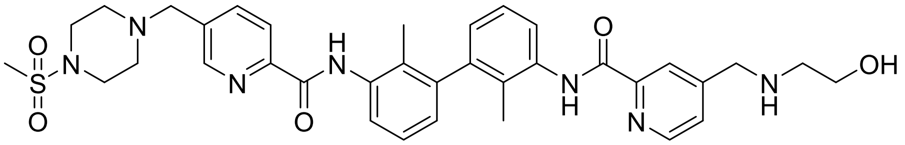

*Synthesis of tert-butyl (2,2'-dimethyl-3'-(5-(piperazin-1-ylmethyl)picolinamido)-[1,1'-biphenyl]-3-yl)carbamate **(1.7_15)***

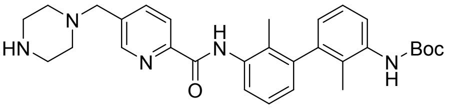

(1.7_15)

**(1.6_2)** (0.5 g, 0.98 mmol, 1 equiv.) was dissolved in ACN (10 ml), then $K_2CO_3$ (0.406 g, 2.94 mmol, 3 equiv.), KI (0.163 g, 0.98 mmol, 1 equiv.) and piperazine (0.169 g, 1.96 mmol, 2 equiv.) were added. Mixture was left for stirring at 50°C for 1 h (no SM). Next, water was added and reaction was extracted with DCM. The organic layer was washed with brine, dried over $Na_2SO_4$ and evaporated to give **(1.7_15)** (0.5 g, 99% yield) as yellowish solid. **$^1$H NMR** (600 MHz, DMSO) δ 10.32 (s, 1H), 8.65 – 8.59 (m, 2H), 8.14 (dd, J = 7.9, 0.9 Hz, 1H), 7.98 (dd, J = 8.0, 2.1 Hz, 1H), 7.85 – 7.81 (m, 1H), 7.31 (dt, J = 15.5, 7.9 Hz, 2H), 7.21 (t, J = 7.8 Hz, 1H), 6.97 – 6.91 (m, 1H), 6.90 (dd, J = 7.5, 1.4 Hz, 1H), 3.59 (s, 2H), 3.35 (s, 1H), 2.73 (t, J = 4.9 Hz, 4H), 2.34 (s, 4H), 1.97 (d, J = 3.0 Hz, 3H), 1.89 (s, 3H), 1.46 (s, 9H). **LC-MS** (DAD/ESI): $t_R$= 6.14 min, Calcd for $C_{30}H_{37}N_5O_3$ (m/z): $[M+H]^+$ 516.30; found, $[M+H]^+$ 516.34, purity: 92%.

*Synthesis of tert-butyl (2,2'-dimethyl-3'-(5-((4-(methylsulfonyl)piperazin-1-yl)methyl) picolinamido)-[1,1'-biphenyl]-3-yl)carbamate **(1.8_15)***

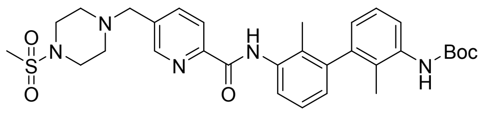

(1.8_15)

Solution of **(1.7_15)** (0.5g, 0.97 mmol, 1 equiv.) in DCM (10 ml) was cooled to 0°C and then TEA (0.276 ml, 1.98 mmol, 1.7 equiv.) was added. Next, methanesulfonyl chloride (0.09 ml, 1.16 ml, 1.2 equiv.) was added and reaction was stirred this temperature for 10 min and then left for overnight stirring at RT. After that time, solid was filtered off and filtrate was extracted with water. The organic layer was concentrated in vacuo to give **(1.8_15)** (0.33 g, 57% yield) as yellowish oil. **$^1$H NMR** (600 MHz, DMSO) δ 10.33 (s, 1H), 8.70 – 8.64 (m, 1H), 8.61 (s, 1H), 8.20 – 8.13 (m, 1H), 8.00 (d, J = 2.1 Hz, 1H), 7.83 (d, J = 7.8 Hz, 1H), 7.31 (dt, J = 15.5, 7.9 Hz, 2H), 7.21 (t, J = 7.7 Hz, 1H), 6.94 (dd, J = 7.6, 1.3 Hz, 1H), 6.90 (dd, J = 7.5, 1.3 Hz, 1H), 3.69 (s, 2H), 3.15 – 3.10 (m, 4H), 2.84 – 2.88 (m, 4H), 1.99 (s, 3H), 1.97 (s, 3H), 1.89 (s, 3H), 1.46 (s, 9H). **LC-MS** (DAD/ESI): $t_R$= 6.82 min, Calcd for $C_{31}H_{39}N_5O_5S$ (m/z): $[M+H]^+$ 594.28; found, $[M+H]^+$ 594.36, purity: 90%.

*Synthesis of N-(3'-amino-2,2'-dimethyl-[1,1'-biphenyl]-3-yl)-5-((4-(methylsulfonyl)piperazin-1-yl)methyl)picolinamide **(1.9_15)***

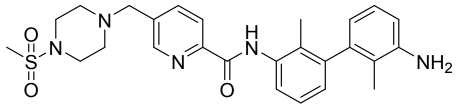

(1.9_15)

**(1.8_15)** (0.33 g, 0.56 mmol, 1 equiv.) was dissolved in DCM (9.9 ml) then 6M HCl in i-PrOH (0.926 ml, 10 equiv.) was added and reaction was left for stirring at RT overnight. Then, solvent was evaporated, residue was extracted with DCM/MeOH/water. To water phase was added KOH water solution (to obtain pH~8) and extracted with DCM/MeOH. to give **(1.9_15)** (0.24 g, 87% yield) as yellowish solid. **$^1$H NMR** (600 MHz, DMSO) δ 10.44 (s, 1H), 8.95 (s, 1H), 8.37 – 8.28 (m, 1H), 8.25 (d, J = 8.0 Hz, 1H), 7.77 (dd, J = 8.1, 1.3 Hz, 1H), 7.41 (s, 1H), 7.35 (dt, J = 11.4, 7.7 Hz, 2H), 7.11 (d, J = 7.3 Hz, 1H), 6.98 (dd, J = 7.6, 1.3 Hz, 1H), 4.53 (s, 2H), 3.37 (s, 2H), 3.23 – 3.27 (m, 4H), 3.22 – 3.17 (m, 4H), 3.00 (s, 3H), 2.03 (s, 3H), 1.96 (s, 3H). **LC-MS** (DAD/ESI): $t_R$= 4.49 min, Calcd for $C_{26}H_{31}N_5O_3S$ (m/z): $[M+H]^+$ 494.22; found, $[M+H]^+$ 494.27, purity: 91%.

*Synthesis of methyl 2-((2,2'-dimethyl-3'-(5-((4-(methylsulfonyl)piperazin-1-yl)methyl)picolinamido)-[1,1'-biphenyl]-3-yl)carbamoyl) isonicotinate **(1.10_15)***

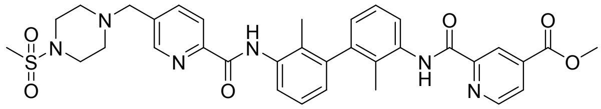

**(1.10_15)**

To a solution of 5-(Methoxycarbonyl)picolinic acid (0.088 g, 0.49 mmol, 1 equiv.) in DMF (7.2 ml) was added TEA (0.203 ml, 1.46 mmol, 3 equiv.). The mixture was cooled to 0°C and treated with HATU (0.203 g, 0.53 mmol, 1.1 equiv.), and the **(1.9_15)** (0.24 g, 0.49 mmol, 1 equiv.). The reaction was stirred at RT overnight. Then water was added and reaction was extracted with DCM. The organic layer was washed with brine, dried over $Na_2SO_4$ and evaporated. To crude was added MTBE and formed solid was filtered off to give **(1.10_15)** (0.33 g) as yellowish solid. **LC-MS** (DAD/ESI): $t_R$= 6.89 min, Calcd for $C_{34}H_{36}N_6O_6S$ (m/z): $[M+H]^+$ 657.25; found, $[M+H]^+$ 657.30, purity: 95%.

*Synthesis of N-(2,2'-dimethyl-3'-(5-((4-(methylsulfonyl) piperazin-1-yl)methyl)picolinamido)-[1,1'-biphenyl]-3-yl)-4-(hydroxymethyl) picolinamide **(1.11_15)***

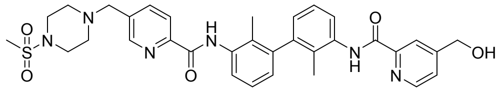

(1.11_15)

**(1.10_15)** (0.33 g, 0.5 mmol, 1 equiv.) was dissolved in THF/MeOH (10:5, 3.3 ml : 1.65 ml) and cooled to 0°C. Then solution of 4M $LiBH_4$ in THF (0.628 ml, 2.51 mmol, 5 equiv.) was added dropwise and reaction was stirred at this temperature for 15 min and then left to warm to RT. Reaction was monitored by TLC. After full consumption of SM (15 min) water was added and solvent was evaporated. To the residue DCM was added and reaction was extracted with DCM/water. The organic layer was washed with brine, dried over $Na_2SO_4$ and evaporated to give **(1.11_15)** (0.3 g) as yellowish oil. **LC-MS** (DAD/ESI): $t_R$= 5.59 min, Calcd for $C_{33}H_{36}N_6O_5S$ (m/z): $[M+H]^+$ 629.25; found, $[M+H]^+$ 629.32, purity: 98%.

*Synthesis of 4-(chloromethyl)-N-(2,2'-dimethyl-3'-(5-((4-(methylsulfonyl) piperazin-1-yl)methyl)picolinamido)-[1,1'-biphenyl]-3-yl)picolinamide **(1.12_15)***

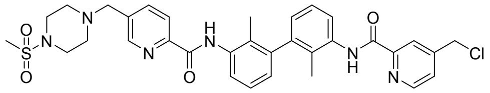

(1.12_15)

Solution of **(1.11_15)** (0.3 g, 0.48 mmol, 1 equiv.) in DCM (6 ml) was cooled to 0°C and then TEA (0.16 ml, 1.15 mmol, 2 equiv.) was added. Next, methanesulfonyl chloride (0.044 ml, 0.57 mmol, 1.2 equiv.) was added and reaction was stirred this temperature for 10 min and then left for overnight stirring at RT. After that time, solid was filtered off and filtrate was extracted with water. The organic layer was concentrated in vacuo to give **(1.12_15)** (0.27 g, 87% yield) as yellowish oil.

*Synthesis of N-(2,2'-dimethyl-3'-(5-((4-(methylsulfonyl) piperazin-1-yl)methyl)picolinamido)-[1,1'-biphenyl]-3-yl)-4-(((2-hydroxyethyl)amino) methyl)picolinamide* ***(15)***

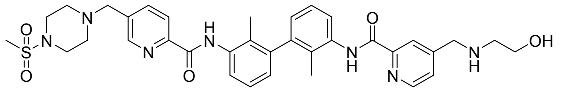

(15)

**(1.12_15)** (0.27 g, 0.42 mmol, 1 equiv.) was dissolved in ACN (5.4 ml), then $K_2CO_3$ (0.173 g, 1.25 mmol, 3 equiv.), KI (0.069 g, 0.42 mmol, 1 equiv.) and ethanolamine (0.05 ml, 0.83 mmol, 2 equiv.) were added. Mixture was left for stirring at 50°C for 3 h (no SM). Next, water was aded and reaction was extracted with DCM. The organic layer was washed with brine, dried over $Na_2SO_4$ and evaporated to give **(15)** (0.053 g, 19% yield) as yellowish solid. **$^1$H NMR** (600 MHz, DMSO) δ 10.34 (d, J = 3.6 Hz, 2H), 8.67 (ddd, J = 6.1, 2.1, 0.8 Hz, 2H), 8.16 (dd, J = 7.9, 0.8 Hz, 1H), 8.13 (dd, J = 8.0, 0.8 Hz, 1H), 8.01 (td, J = 8.0, 2.1 Hz, 2H), 7.88 (td, J = 8.3, 1.3 Hz, 2H), 7.33 (t, J = 7.8 Hz, 2H), 6.99 (ddd, J = 7.6, 3.5, 1.3 Hz, 2H), 4.50 (t, J = 5.4 Hz, 1H), 3.85 (s, 2H), 3.69 (s, 2H), 3.47 (q, J = 5.7 Hz, 2H), 3.32 – 3.28 (m, 5H), 3.18 – 3.13 (m, 4H), 2.88 (s, 3H), 2.58 (t, J = 5.8 Hz, 2H), 2.02 (s, 6H). **LC-MS** (DAD/ESI): $t_R$= 4.44 min, Calcd for $C_{35}H_{41}N_7O_5S$ (m/z): $[M+H]^+$ 672.30; found, $[M+H]^+$ 672.39, purity: 100%.

**Compound (17)**

**N-(2,2'-dimethyl-3'-(5-(((3-(methylsulfonamido)propyl)amino)methyl)picolinamido)-[1,1'-biphenyl]-3-yl)-5-(((2-hydroxyethyl)amino)methyl)picolinamide**

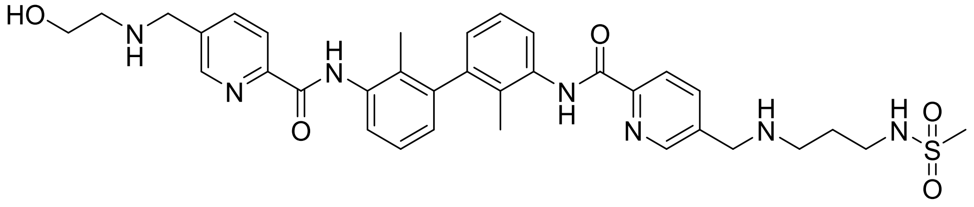

*Synthesis of tert-butyl (3-aminopropyl)carbamate* ***(1.2_17)***

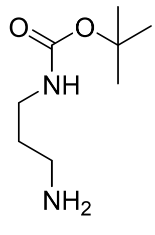

**(1.2_17)**

A solution of di-tert-butyl dicarbonate (2.65 g, 12.14 mmol, 0.2 equiv.) in DCM (45 ml) was added dropwise to a solution of 1,3-Diaminopropane **(1.1_17)** (4.5 g, 60.7 mmol, 1 equiv.) in DCM (45 ml) at 0°C with vigorous stirring. Stirring was continued for a further 24 h at room temperature. After concentration to an oily residue, the reaction mixture was dissolved in aqueous 2M sodium carbonate and extracted with dichloromethane. The organic layer was washed

with 2M sodium carbonate and dried over anhydrous $MgSO_4$. The solvent was evaporated under reduced pressure to give **(1.2_17)** (3.4 g of crude as white solid. **$^1$H NMR** (600 MHz, DMSO) δ 6.78 (t, *J* = 5.7 Hz, 1H), 2.95 (q, *J* = 6.5 Hz, 2H), 2.52 (d, *J* = 7.1 Hz, 2H), 1.43 (t, *J* = 6.8 Hz, 2H), 1.37 (s, 9H).

*Synthesis of tert-butyl (3-(methylsulfonamido)propyl) carbamate **(1.3_17)***

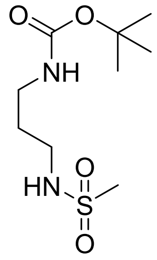


**(1.3_17)**

To a stirred solution of **(1.2_17)** (3.4 g, 19.15 mmol, 1 equiv.) and TEA (5.44 ml, 39.03 mmol, 3 equiv.) in DCM (68 ml) was added methanesulfonyl chloride (1.664 ml, 21.46 mmol, 1.1 equiv.) at 0° C and reaction was stirred at this temperature for 10 min and then left for stirring at RT for 4 h. After that time, water was added and mixture was extracted with DCM. The organic layer was washed with brine, dried over $Na_2SO_4$ and concentrated in vacuo to give **(1.3_17)** (2.72 g, crude) as yellowish oil. **$^1$H NMR** (600 MHz, DMSO) δ 6.91 (t, *J* = 5.9 Hz, 1H), 6.80 (t, *J* = 5.8 Hz, 1H), 2.96 – 2.90 (m, 2H), 2.87 (s, 3H), 1.56 (p, *J* = 7.0 Hz, 2H), 1.37 (d, *J* = 3.3 Hz, 9H), 1.08 (t, *J* = 7.3 Hz, 2H).

*Synthesis of N-(3-aminopropyl)methanesulfonamide **(1.4_17)***

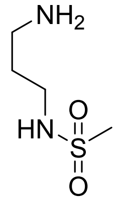


**(1.4_17)**

**(1.3_17)** (2.72 g, 10.78 mmol, 1 equiv.) was dissolved in DCM (81.30 ml) then 6M HCl in i-PrOH (17.966 ml, 10 equiv.) was added and reaction was left for stirring at RT overnight. Then, solvent was evaporated, residue was extracted with DCM/MeOH/water. Water phase was evaporated to give **(1.4_17)** (1.92 g, crude) as yellowish oil. **$^1$H NMR** (600 MHz, DMSO) δ 8.12 (s, 1H), 7.97 (s, 3H), 7.15 (t, *J* = 6.0 Hz, 1H), 3.90 – 3.70 (m, 3H), 3.09 – 2.97 (m, 4H), 2.90 (s, 3H), 2.81 (ddd, *J* = 8.9, 7.5, 5.8 Hz, 2H), 1.75 (dq, *J* = 8.8, 7.0 Hz, 2H).

*Synthesis of tert-butyl (2-((tert-butyldimethylsilyl)oxy) ethyl)((6-((2,2'-dimethyl-3'-(5-(((3-(methylsulfonamido)propyl)amino)methyl) picolinamido)-[1,1'-biphenyl]-3-yl)carbamoyl) pyridin-3-yl)methyl)carbamate **(1.15_17)***

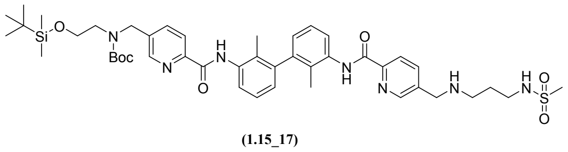

(1.15_17)

**(1.14_17)** (0.29 g, 0.38 mmol, 1 equiv.) was dissolved in ACN (5.8 ml), then $K_2CO_3$ (0.317 g, 2.29 mmol, 6 equiv.), KI (0.063 g, 0.38 mmol,

1equiv.) and **(1.4_17)** (0.175 g, 1.15 mmol, 3 equiv.) were added. Mixture was left for stirring at 50°C for 1 h (no SM). Next, water was aded and reaction was extracted with DCM. The organic layer was washed with brine, dried over $Na_2SO_4$ and evaporated. Crude was purified by flash chromatography using DCM:MeOH (0-30%) to give **(1.15_17)** (0.2 g, 34% yield) as yellowish solid.

*Synthesis of N-(2,2'-dimethyl-3'-(5-(((3-(methylsulfonamido) propyl)amino)methyl) picolinamido)-[1,1'-biphenyl]-3-yl)-5-(((2-hydroxyethyl)amino) methyl)picolinamide **(17)***

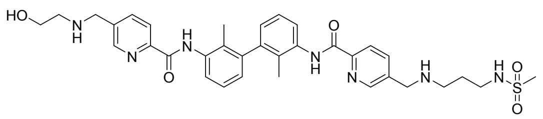

(17)

**(1.15_17)** (0.18 g, 0.21 mmol, 1 equiv.) was dissolved in DCM (5.4 ml) then 6M HCl in i-PrOH (0.343 g, 2.06 mmol, 10 equiv.) was added and reaction was left for stirring at RT overnight. Then solvent was evaporated and crude was initially purified by flash chromatagraphy using DCM:MeOH (0-50%). Then to final fraction was added small emount of MeOH and then MTBE. Formed solid was filtered off to give **(17)** as yellowish solid. **$^1$H NMR** (600 MHz, MeOD) δ 8.87 – 8.81 (m, 2H), 8.33 (ddd, J = 8.1, 1.7, 0.9 Hz, 2H), 8.19 (ddd, J = 8.1, 3.6, 2.2 Hz, 2H), 7.87 (d, J = 8.0 Hz, 2H), 7.35 (t, J = 7.8 Hz, 2H), 7.08 (dd, J = 7.6, 1.3 Hz, 2H), 4.44 (s, 2H), 4.42 (s, 2H), 3.88 – 3.84 (m, 2H), 3.28 – 3.22 (m, 6H), 2.96 (s, 3H), 2.10 (s, 6H), 2.07 (d, J = 5.7 Hz, 1H), 2.01 – 1.95 (m, 2H). **$^{13}$C NMR** (151 MHz, MeOD) δ 163.97, 151.93, 151.89, 151.27, 151.25, 143.75, 140.95, 140.92, 137.05, 131.99, 131.93, 130.54, 128.21, 127.17, 124.09, 123.47, 123.45, 57.78, 50.67, 46.65, 40.88, 39.67, 28.08, 14.92. **LC-MS** (DAD/ESI): $t_R$= 4.47 min, Calcd for $C_{34}H_{41}N_7O_5S$ (m/z): $[M-H]^-$ 658.28; found, $[M-H]^-$ 658.24, purity: 95%. **HRMS** (ESI): Calcd for $C_{34}H_{41}N_7O_5S$ (m/z): $[M+H]^+$ 660.2968; found, $[M+H]^+$ 660.3022, purity: 95%.

**Compound (17a)**

**N-(2,2'-dimethyl-3'-(5-(((3-(methylsulfonamido)propyl)amino)methyl)picolinamido)-[1,1'-biphenyl]-3-yl)-5-(((2-hydroxyethyl)amino)methyl)picolinamide tetrahydrochloride**

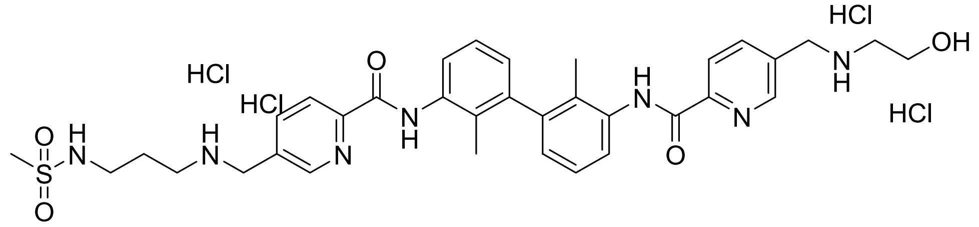

*Synthesis:*

**(17)** (0.028 g, 0.04 mmol, 1 equiv.) was dissolved in DCM (0.84 ml) then 6M HCl in i-PrOH (0.071 ml, 10 equiv.) was added and reaction was left for stirring at RT overnight. Then solvent was evaporated to give **(17a)** as yellowish solid. **$^{1}$H NMR** (600 MHz, DMSO) δ 10.41 (d, *J* = 1.8 Hz, 2H), 9.30 (d, *J* = 30.4 Hz, 4H), 8.87 (dt, *J* = 7.3, 1.5 Hz, 2H), 8.27 – 8.21 (m, 3H), 7.80 (dd, *J* = 8.0, 3.2 Hz, 2H), 7.34 (t, *J* = 7.8 Hz, 2H), 7.16 (t, *J* = 6.1 Hz, 1H), 7.02 (dd, *J* = 7.6, 1.3 Hz, 2H), 4.34 (dt, *J* = 9.4, 5.5 Hz, 4H), 3.70 (t, *J* = 5.3 Hz, 2H), 3.03 (p, *J* = 7.2, 6.8 Hz, 6H), 2.91 (s, 3H), 2.01 (s, 6H), 1.86 (p, *J* = 6.9 Hz, 2H). **$^{13}$C NMR** (151 MHz, DMSO) δ 161.86, 161.76, 150.24, 149.76, 141.85, 140.14, 136.15, 136.03, 129.29, 129.08, 126.40, 125.85, 123.18, 121.89, 56.34, 56.31, 48.84, 48.77, 47.00, 46.92, 44.41, 44.34, 39.93, 39.80, 39.66, 39.52, 39.38, 39.24, 39.10, 14.64, 14.60. **LC-MS** (DAD/ESI): $t_R$= 3.78 min, Calcd for $C_{34}H_{41}N_7O_5S$ (m/z): [M-H]$^-$ 658.28; found, [M-H]$^-$ 658.43, purity: 95%. **HRMS** (ESI): Calcd for $C_{34}H_{41}N_7O_5S$ (m/z): [M+H]$^+$ 660.2968; found, [M+H]$^+$ 660.2923, purity: 95%.

**Compound (6)**

**methyl ((6-((2,2'-dimethyl-3'-(5-(pyrrolidin-1-ylmethyl)picolinamido)-[1,1'-biphenyl]-3-yl)carbamoyl)pyridin-3-yl)methyl)alaninate**

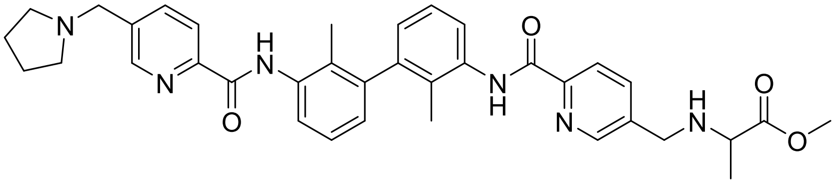

*Synthesis of N-(3'-amino-2,2'-dimethyl-[1,1'-biphenyl]-3-yl)-5-(chloromethyl)picolinamide* ***(1.8_6)***

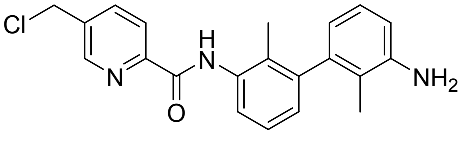

**(1.7_6)**

**(1.6_2)** (0.28 g, 0.81 mmol, 1 equiv.) was dissolved in DCM (2.8 ml) and then thionyl chloride (0.292 ml, 4.03 mmol, 5 equiv.) was added. Reaction was stirred at RT overweekend. Next, water was added and reaction was extracted with DCM. The organic layer was washed with brine and evaporated to give **(1.7_6)** (0.36 g) as yellowish solid. **$^{1}$H NMR** (600 MHz, DMSO) δ 10.32 (d, *J* = 8.3 Hz, 1H), 8.84 – 8.80 (m, 1H), 8.19 (dt, *J* = 8.0, 1.1 Hz, 1H), 8.14 (dt, *J* = 8.1, 1.9 Hz, 1H), 7.82 – 7.75 (m, 1H), 7.26 (t, *J* = 7.7 Hz, 1H), 7.01 – 6.92 (m, 2H), 6.65 (dd, *J* =

8.0, 1.3 Hz, 1H), 6.31 (dd, *J* = 7.4, 1.3 Hz, 1H), 4.94 (s, 2H), 4.92 (s, 2H), 1.99 (d, *J* = 3.0 Hz, 3H), 1.74 (s, 3H). **LC-MS** (DAD/ESI): $t_R$= 6.39 min, Calcd for $C_{21}H_{20}ClN_3O$ (m/z): $[M+H]^+$ 366.14; found, $[M+H]^+$ 366.28, purity: 93%.

*Synthesis of methyl 6-(chlorocarbonyl)nicotinate **(1.8_6)***

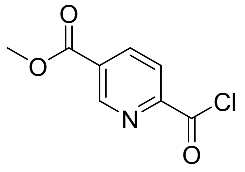

**(1.8_6)**

5-(Methoxycarbonyl)picolinic acid **(1.8.1_6)** (0.5 g, 2.76 mmol, 1 equiv.) was dissolved in toluene (2.5 ml, 5 equiv.) and thionyl chloride (0.801 ml, 11.04 mmol, 10 equiv.) was added and reaction wa stirred at 90°C overnight. After that reaction was cooled to RT and excess of chloride was evaporated to give (**1.8_6)** (0.57 g) as yellowish solid.

*Synthesis of methyl 6-((3'-(5-(chloromethyl)picolinamido)-2,2'-dimethyl-[1,1'-biphenyl]-3-yl)carbamoyl)nicotinate **(1.9_6)***

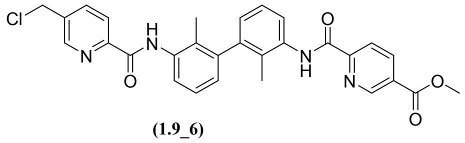

**(1.9_6)**

**(1.7_6)** (0.35 g, 0.96 mmol, 1 equiv.) was dissolved in THF (10.5 ml) and **(1.8_6)** (0.191 g, 0.96 mmol, 1 equiv.) was added followed the TEA (0.333 ml, 2.39 mmol, 2.5 equiv.) at 0°C. Next, reaction was stirred at RT overnight. Next water was added and reaction was extracted with EtOAc. The organic layer was washed with brine, dried over $Na_2SO_4$ and evaporated. Crude was purified by flash chromatography using Hex:EtOAc (0-100%) to give **(1.9_6)** (0.092 g,18% yield) as yellowish solid. **LC-MS** (DAD/ESI): $t_R$= 8.50 min, Calcd for $C_{29}H_{25}ClN_4O_4$ (m/z): $[M+H]^+$ 529.16; found, $[M+H]^+$ 529.30, purity: 90%.

*Synthesis of 5-(chloromethyl)-N-(3'-(5-(hydroxymethyl) picolinamido)-2,2'-dimethyl-[1,1'-biphenyl]-3-yl)picolinamide **(1.10_6)***

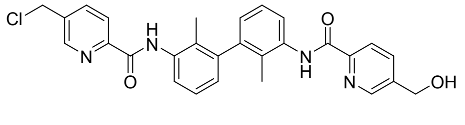

**(1.10_6)**

**(1.9_6)** (0.092 g, 0.17 mmol, 1 equiv.) was dissolved in THF/MeOH (30:10, 2.76 ml : 0.92 ml) and cooled to 0°C. Then 4 M solution of $LiBH_4$ in THF (0.13 ml, 0.52 mmol, 3 equiv.) was added and reaction was stirred for 15 min at this temperature. Next reaction was left to warm to RT and stirred for additional 1 h (no SM). After that time water was added and reaction was extracted with EtOAc. The organic layer was washed with brine, dried over $Na_2SO_4$ and evaporated to give **(1.10_6)** (0.065 g, 75% yield) as yellowish solid. **$^1$H NMR** (300

MHz, DMSO) δ 10.34 (d, J = 5.2 Hz, 2H), 8.88 – 8.77 (m, 1H), 8.66 (dd, J = 2.2, 0.9 Hz, 1H), 8.16 (ddd, J = 7.9, 4.9, 0.9 Hz, 3H), 8.00 (dd, J = 8.0, 2.0 Hz, 1H), 7.93 – 7.84 (m, 2H), 7.34 (d, J = 7.7 Hz, 2H), 7.00 (t, J = 6.2 Hz, 2H), 5.50 (t, J = 5.7 Hz, 1H), 4.94 (s, 2H), 4.66 (d, J = 5.7 Hz, 2H), 2.02 (s, 6H). **LC-MS** (DAD/ESI): $t_R$= 7.20 min, Calcd for $C_{28}H_{25}ClN_4O_3$ (m/z): $[M+H]^+$ 501.17; found, $[M+H]^+$ 501.32, purity: 90%.

*Synthesis of N-(2,2'-dimethyl-3'-(5-(pyrrolidin-1-ylmethyl)picolinamido)-[1,1'-biphenyl]-3-yl)-5-(hydroxymethyl)picolinamide* ***(1.11_6)***

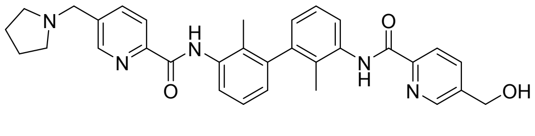


**(1.11_6)**

**(1.10_6)** (0.065 g, 0.13 mmol, 1 equiv.) was dissolved in THF (1.95 ml) and pyrrolidine (0.013 ml, 0.16 mmol, 1.2 equiv.) was added followed the TEA (0.054 ml, 0.39 mmol, 3 equiv.) at 0°C. Next, reaction was stirred at RT overnight. Next water was added and reaction was extracted with EtOAc to give **(1.11_6)** (0.065 g, 94% yield) as yellowish solid. **$^1$H NMR** (600 MHz, DMSO) δ 10.34 (d, J = 3.0 Hz, 2H), 8.69 – 8.64 (m, 2H), 8.14 (d, J = 7.6 Hz, 2H), 7.99 (ddd, J = 8.1, 4.2, 2.1 Hz, 2H), 7.88 (ddd, J = 8.0, 2.8, 1.3 Hz, 2H), 7.33 (t, J = 7.8 Hz, 2H), 6.99 (dt, J = 7.8, 2.1 Hz, 2H), 5.51 (t, J = 5.7 Hz, 1H), 4.66 (d, J = 5.5 Hz, 2H), 3.72 (s, 2H), 2.48 – 2.45 (m, 3H), 2.02 (d, J = 1.8 Hz, 6H), 1.71 (p, J = 3.0 Hz, 3H), 1.65 (ddd, J = 7.9, 5.2, 2.7 Hz, 2H). **LC-MS** (DAD/ESI): $t_R$= 4.67 min, Calcd for $C_{32}H_{33}N_5O_3$ (m/z): $[M+H]^+$ 536.27; found, $[M+H]^+$ 536.34, purity: 95%.

*Synthesis of N-(2,2'-dimethyl-3'-(5-(pyrrolidin-1-ylmethyl)picolinamido)-[1,1'-biphenyl]-3-yl)-5-formylpicolinamide* ***(1.12_6)***

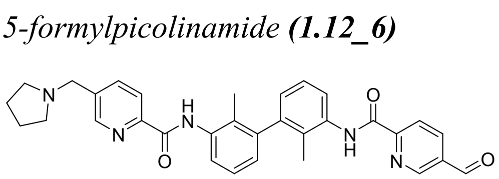


**(1.12_6)**

**(1.11_6)** (0.065 g, 0.12 mmol, 1 equiv.) was dissolved in DCM (1.95 ml) and cooled to 0°C. Then, Dess-Martin periodinane (0.103 g, 0.24 mol, 2 equiv.) and $NaHCO_3$ (0.051 g, 0.61 mmol, 5 equiv.) were added and reaction was stirred for 0.5 h at 0°C. Next reaction was left warm to RT and stirred for 30 min (no SM). After that time $NaHCO_3$ was added and reaction was extracted with water. The organic layer was washed with brine, dried over $Na_2SO_4$ and evaporated to give **(1.12_6)** (0.026 g, 40% yield) as yellowish solid. **$^1$H NMR** (600 MHz, DMSO) δ 10.52 (s, 1H), 10.35 (s, 1H), 10.24 (s, 1H), 9.23 (dd, J = 2.1, 0.9 Hz, 1H), 8.66 (d, J = 2.1 Hz, 1H), 8.52 (dd, J = 8.0, 2.0 Hz, 1H), 8.37 (d, J = 8.0 Hz, 1H), 8.16 (s, 1H), 7.87 (ddd, J = 17.0, 8.0, 1.1 Hz, 2H), 7.82 – 7.77 (m, 1H), 7.73 – 7.70 (m, 1H), 7.34 (q,

J = 7.4 Hz, 2H), 7.04 (dd, J = 7.6, 1.3 Hz, 1H), 3.73 (s, 2H), 2.52 – 2.48 (m, 4H), 2.03 (d, J = 1.7 Hz, 6H), 1.74 – 1.69 (m, 4H). **LC-MS** (DAD/ESI): $t_R$= 5.31 min, Calcd for $C_{32}H_{31}N_5O_3$ (m/z): $[M-H]^-$ 532.23; found, $[M-H]^-$ 532.16, purity: 90%.

*Synthesis of methyl alaninate dihydrochloride **(1.13_6)***

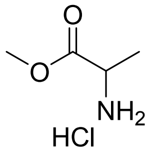


**(1.13_6)**

DL-Alanine **(1.13.1_6)** (4 g, 44.9 mmol, 3 equiv.) was dissolved in MeOH (80 ml, 20 equiv.) and cooled using ice-bath to 0°C. Then, slowly was added thionyl chloride (16.285 ml, 224.49 ml, 5 equiv.) and reaction was left for stirring at this temperature for 15 min. Then ice-bath was removed and reaction was left for stirring at reflux overnight. After the reaction completed, solvent was distilled off with an excess of thionyl chloride to give **(1.13_6)** (6.37 g, 81% yield) as brown solid. $^{1}$**H NMR** (600 MHz, DMSO) δ 8.56 (s, 3H), 4.07 (d, *J* = 7.8 Hz, 1H), 3.74 (s, 3H), 2.50 (p, *J* = 1.8 Hz, 3H).

*Synthesis of methyl ((6-((2,2'-dimethyl-3'-(5-(pyrrolidin-1-ylmethyl)picolinamido)-[1,1'-biphenyl]-3-yl)carbamoyl)pyridin-3-yl)methyl)alaninate **(6)***

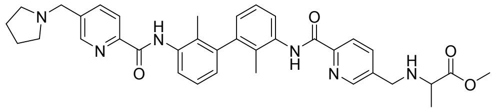

**(6)**

**(1.12_6)** (0.026 g, 0.05 mmol, 1 equiv.), **(1.13_6)** (0.026 g, 0.15 mmol, 3 equiv.), TEA (0.02 ml, 0.15 mmol, 3 equiv.) and few drops of AcOH were dissolved in a mixture of DCE/MeOH/DMF (20:10:20, 0.52 ml : 0.26 ml : 0.26 ml) and left for stirring for 2 h. Then $NaBH_3CN$ (0.015 g, 0.24 mmol, 5 equiv.) was added and reaction was left for overnight stirring. After that, water was added and reaction was extracted with EtOAc. The organic layer was washed with brine, dried over $Na_2SO_4$ and evaporated. Crude was purified by flash chromatography using EtOAc:MeOH (8:2) to give **(6)**. **LC-MS** (DAD/ESI): $t_R$= 4.19 min, Calcd for $C_{36}H_{40}N_6O_4$ (m/z): $[M+H]^+$ 621.32; found, $[M+H]^+$ 621.41, purity: 100%.

## SOLUBILITY OF THE COMPOUNDS

For solubility studies, samples were prepared by dissolving 1-2 mg of an inhibitor in 100-300 µL DMSO to give a stock of 50 mM of the compound. 100-fold dilutions of the compounds were prepared in medium (RPMI1640, 1% FBS, 0.5% DMSO) (i.e., the concentration of the

compound was 500 nM). The dilutions were then visually evaluated according to the following scheme

+++ clear solution, no precipitation

++ slight turbidity, no precipitate

+ visible turbidity, no precipitate.

+/- strong turbidity, no precipitation.

- visible precipitation and/or visible precipitation in the original 100% DMSO solution.

The dilutions prepared in this way were applied to a 96-well plate and the absorbance measured at a wavelength of 600 nm. The results averaged from two measurements are summarized in **Table S2**, Blank of 10 Measurements. For very poorly soluble compounds, the absorbance measurement (shown in parentheses) is rather unreliable because it cannot account for the precipitate to such an extent.

**Table S2.** Solubility of the compounds

| ID number | Visual evaluation | A600 measurement (averaged from 2 reps) |
|---|---|---|
| 1 | +/- | 0.51375 |
| 2 | +/- | 0.40235 |
| 2a | ++ | 0.1517 |
| 3 | +/- | 0.27855 |
| 4 | +/- | 0.2271 |
| 5 | ++ | 0.04735 |
| 6 | - | (0.0627) |
| 7 | +/- | 0.3466 |
| 8 | +/- | 0.459 |
| 9 | + | 0.09395 |
| 10 | +/- | 0.1308 |
| 11 | - | (0.1117) |
| 12 | +++ | 0.0476 |
| 13 | - | (0.1827) |
| 14 | +++ | 0.05315 |
| 15 | +/- | 0.45095 |
| 16 | +++ | 0.0494 |
| 17 | +/- | 0.2217 |
| 17a | ++ | 0.2848 |

| | | |
|---|---|---|
| Blank | N/A | 0.0472 |

**The HTRF test:**

Samples were prepared by dissolving 1-2 mg of inhibitor in 100-300 µL DMSO to give a stock of 50 mM of the compound. Samples were then diluted to 50 nM and used for measurements. No precipitation of compounds was observed during the preparation of the stock for the HTRF assay.
